# Metal thiosemicarbazonates as dual mR2 RNR and colchicine-site tubulin inhibitors: from biochemical inhibition to mitotic arrest in MCF-7 breast cancer cells

**DOI:** 10.1039/d6qi01591c

**Published:** 2026-09-03

**Authors:** Oleg Palamarciuc, Bianca-Iulia Ciubotaru, Alexandru-Constantin Stoica, Michaela Hejl, Marta Hammerstad, Mihaela Balan-Porcarasu, Sergiu Shova, Gerda T. Gátszegi, Ana Popović Bijelić, Jóhannes Reynisson, Michael A. Jakupec, Michal Zalibera, Peter Rapta, Éva A. Enyedy, Dragos Peptanariu, Mihaela Dascalu, Maria Cazacu, Ernest Hamel, Vladimir B. Arion

**Affiliations:** a Department of Inorganic Polymers, “Petru Poni” Institute of Macromolecular Chemistry, Romanian Academy Aleea Gr. Ghica Voda 41 A Iasi 700487 Romania; b Physics of Semiconductors and Devices Laboratory, Faculty of Physics and Engineering and Institute of Applied Physics, Moldova State University MD-2009 Chişinău Republic of Moldova; c Department of Biomedical Sciences, Faculty of Medical Bioengineering, Grigore T. Popa University of Medicine and Pharmacy 11-13 Kogalniceanu Street 700454 Iasi Romania; d Institute of Inorganic Chemistry, Faculty of Chemistry, University of Vienna Währinger Strasse 42 A-1090 Vienna Austria vladimir.arion@univie.ac.at; e Section for Biochemistry and Molecular Biology, Department of Biosciences, University of Oslo P.O. Box 1066 Blindern NO-0316 Oslo Norway; f Department of Polycondensation and Thermostable Polymers, “Petru Poni” Institute of Macromolecular Chemistry, Romanian Academy Aleea Gr. Ghica Voda 41 A Iasi 700487 Romania vladimir.arion@univie.ac.at; g Department of Molecular and Analytical Chemistry, University of Szeged Dóm tér 7-8 H-6720 Szeged Hungary; h Faculty of Physical Chemistry, University of Belgrade 11158 Belgrade Serbia; i School of Allied Health Professions and Pharmacy, Keele University Hornbeam Building Staffordshire ST5 5BG UK; j Institute of Physical Chemistry and Chemical Physics, Faculty of Chemical and Food Technology, Slovak University of Technology in Bratislava SK-81237 Bratislava Slovakia; k Centre of Advanced Research in Bionanoconjugates and Biopolymers, “Petru Poni” Institute of Macromolecular Chemistry, Romanian Academy Grigore Ghica Voda Alley 41 A 700487 Iasi Romania peptanariu.dragos@icmpp.ro; l Molecular Pharmacology Branch, Developmental Therapeutics Program, Division of Cancer Treatment and Diagnosis, National Cancer Institute, Frederick National Laboratory for Cancer Research, National Institutes of Health Frederick Maryland 21702 USA

## Abstract

Cancer cells exploit multiple proliferation pathways, making single-target therapies vulnerable to resistance. Here, a thiosemicarbazone-based strategy is introduced for striking two validated oncotargets, the R2 subunit of ribonucleotide reductase (RNR) and the colchicine site of tubulin, with one agent. Guided by molecular docking, quinoline-8-carboxaldehyde *N*4-(3,4,5-trimethoxyphenyl)thiosemicarbazone (HL) and its metal complexes **[Cu**^**II**^**(L)(Cl**_**2**_**CHCOO)]** (1), **[Cu**^**II**^**(L)Cl]** (2), **[Pd**^**II**^**(L)Cl]** (3), and **[Fe**^**III**^**(L)_2_]Cl** (4) were synthesized and characterized by spectroscopic, spectrometric, electrochemical, and crystallographic techniques. Complexes 1, 2, and 4 effectively quench the R2 tyrosyl radical and inhibit tubulin polymerization in cell-free assays, yet only coordination to Cu(ii) translates this activity into sub- to low-micromolar cytotoxicity toward MCF-7 breast cancer cells, while the parent Schiff base and its Pd(ii) and Fe(iii) complexes remain largely inactive (HL and 3) or show somewhat reduced activity (4). Strikingly, cell cycle profiling at a single treatment time point shows that this activity manifests predominantly as mitotic accumulation mirroring the microtubule poison nocodazole, with no detectable S-phase signature. A time-dependent analysis would be required to determine whether an earlier or transient S-phase perturbation precedes this mitotic phenotype; therefore, the present data do not establish the proposed temporal relationship between RNR inhibition and mitotic arrest. These findings establish Cu(ii) thiosemicarbazonates as a dual-target chemotype, while the relative contribution and temporal sequence of the two cellular activities remain to be resolved.

## Abstract 2

A single quinoline-thiosemicarbazone scaffold is engineered to strike two cancer targets at once, ribonucleotide reductase R2 protein and the colchicine site of tubulin. Its Cu(ii) complexes reach sub-micromolar potency against MCF-7 breast cancer cells, driving them into mitotic arrest.

## Graphical abstract

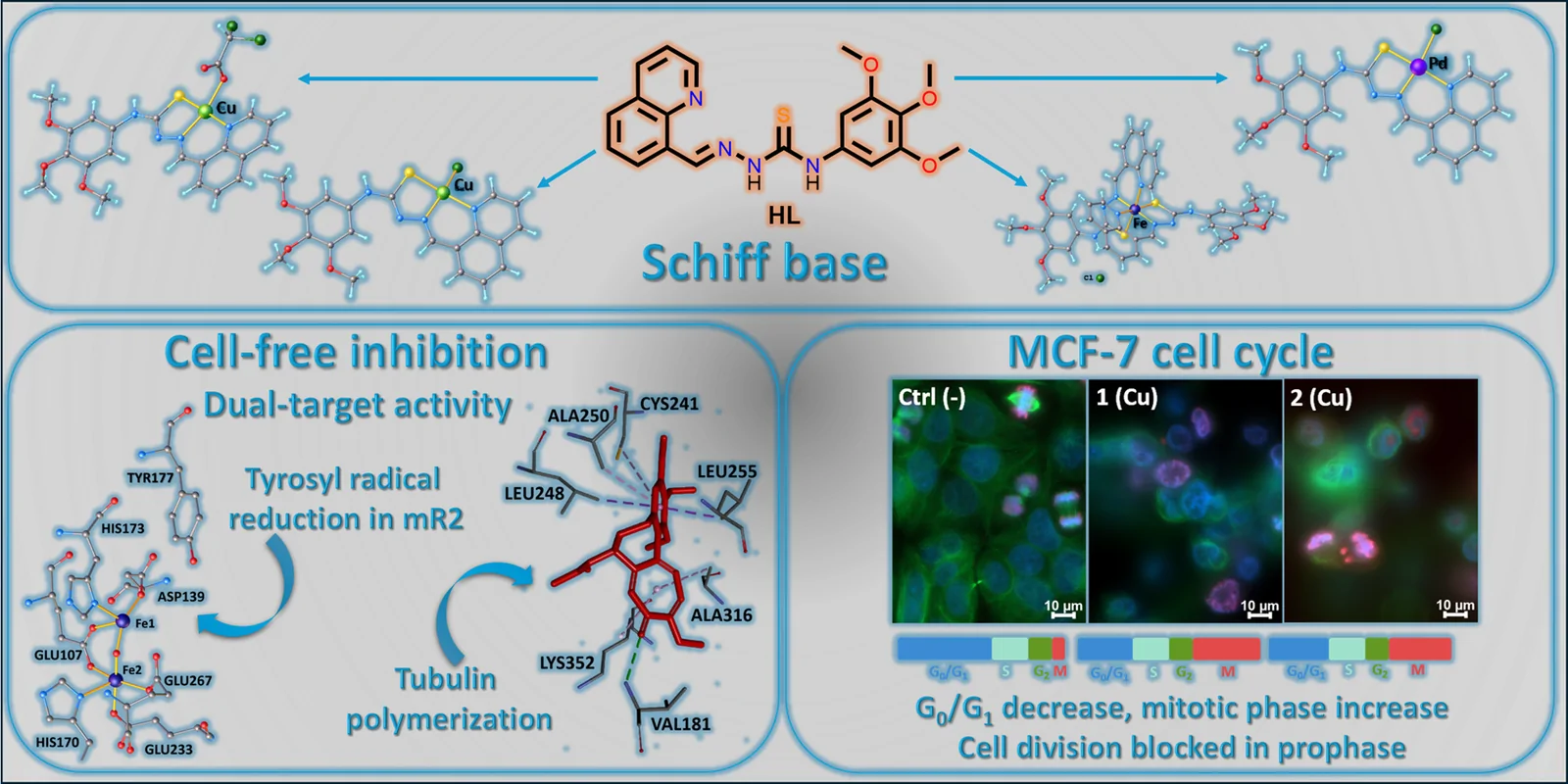

## Introduction

Cancer is a cunning disease that is difficult to treat. Although modern anticancer chemotherapy has made progress, there are still many problems caused by drug resistance and by the low selectivity of anticancer drugs generating serious side effects. Therefore, the search for new chemotherapeutics remains an essential challenge.

Many validated oncotargets are well documented.^1^ Among them the R2 subunit of the ribonucleotide reductase (RNR) enzyme, and tubulin, the repeating subunit of microtubules (MTs), attracted our attention for the development of anticancer drugs, as they play crucial roles in cell proliferation and other vital cellular processes. The R2 protein (Fig. S1 in the SI) contains a diferric tyrosyl radical cofactor (Fe^III^_2_–Y˙) required for the catalytic conversion of ribonucleotides to deoxyribonucleotides, a process that is fundamental to DNA-based life.^2^ Tubulin, one of the most attractive molecular targets for antitumor drugs,^3^ plays crucial roles in cell division,^4^ maintaining cell shape and structure.^5–7^ MTs are also involved in other vital cellular processes, such as cell signaling and intracellular transport.^6,8,9^ Interference with MT dynamics by MT-targeted agents (MTAs) during mitosis perturbs chromosome separation dependent on the mitotic spindle. This disrupts cell cycle progression and usually results in cell death.^10,11^ Structural biology has disclosed multiple binding sites for MTAs, including the taxane and laulimalide sites for MT-stabilizing agents (MSAs) and the colchicine, vinca alkaloid, maytansine, eribulin, pironetin and gatorbulin sites for MT-destabilizing agents (MDAs), opening new possibilities for creating innovative anticancer drugs.^12^

The clinical application of MTAs has advanced significantly. More than 20 new MTA drugs for treatment of cancer and other diseases are available on the market. Nevertheless, the efficacy of using MTAs against cancer is still limited by the development of drug resistance, high systemic toxicity and low aqueous solubility of known inhibitors. The combination of MTAs with other anticancer drugs is a widely recognized strategy to mitigate these drawbacks. In addition, tubulin inhibitors targeting the colchicine site are also candidates for better clinical outcomes. Colchicine site inhibitors (CSIs) target the interface between the *α* and *β* subunits of tubulin heterodimers (Fig. S2). They are less vulnerable to transporter-mediated drug resistance (*e.g.*, overexpression of ABC transporters) and possess potent cytotoxic effects against cancer cells overexpressing β3-tubulin. Tubulin polymerization inhibitors targeting the colchicine binding pocket, which advanced through different phases of clinical trials, include plinabulin (BPI-2358) for cancer therapy and prevention of chemotherapy-induced neutropenia,^13,14^ fosbretabulin (combretastatin A-4 phosphate), which was tested as a vascular-disrupting agent for advanced solid tumors and thyroid cancer,^15^ lexibulin (CYT-997) investigated against solid tumors and multiple myeloma,^16,17^ and azabepintol (MPC-6827) studied in patients with advanced cancers.^18^

R2 RNR inhibitors and MT-targeted agents may lead to disruption of cell cycle progression and cell death *via* cell cycle arrest in S and G_2_/M phases, respectively.

Thiosemicarbazones (TSCs) represent a versatile class of metal-binding compounds with well-established anticancer activity.^19,20^ Although initially developed as iron chelators and inhibitors of ribonucleotide reductase,^21–26^ with triapine as the most relevant example of the anticancer drug candidate tested in more than 30 phase I and II clinical trials so far, and, currently in phase III clinical trials for the treatment of cervical and vaginal cancer,^27,28^ extensive studies on the di-2-pyridylketone thiosemicarbazones Dp44mT and DpC have demonstrated that these compounds possess multiple mechanisms of action, including perturbation of cellular iron homeostasis, formation of redox-active metal complexes, induction of oxidative stress, lysosomal targeting, and modulation of several signaling pathways.^20,29,30^ Recently, the tubulin inhibition potential of TSCs was also disclosed,^31–33^ even though the number of metal complexes with organic ligands as tubulin inhibitors reported so far is scarce.^34–40^

In a long-term project started recently, we aimed to produce more potent colchicine inhibitors with high antiproliferative activity. In addition, we noticed that the potential of Cu(ii) complexes of TSCs as single anticancer drugs with dual action as R2 RNR and tubulin inhibitors has not been explored so far. Our interest in this specific bioactivity of Cu(ii) complexes was fueled by our recent finding that Cu(ii) complexes with morpholine-substituted 2-formylpyridine thiosemicarbazone hybrids^32^ inhibit both R2 RNR and tubulin polymerization in cell-free settings. Further exploration of this discovery would fit with the current practice to combine MTAs with other anticancer drugs to enhance therapeutic results.^41,42^ A single agent with such dual action should induce cell cycle arrest with unexpected advantages.^43^

In the current study, we searched for innovative metal thiosemicarbazonates as inhibitors of both R2 RNR and tubulin polymerization in cell-free settings and the evaluation of their anticancer potential *in vitro*. To achieve this goal, the following work was performed: (i) the preliminary *in silico* screening of thiosemicarbazones derived from substituted *N*4-phenylthiosemicarbazides and quinoline-8-carboxaldehyde and their metal complexes able to associate with the colchicine binding site and selection of the most prospective candidate(s) followed by (ii) synthesis of chosen quinoline-8-carboxaldehyde *N*4-(3,4,5-trimethoxyphenyl)thiosemicarbazone (HL) and Cu(ii), Pd(ii) and Fe(iii) complexes of 1 : 1 and 1 : 2 metal-to-ligand stoichiometry and dichloroacetato (DCA) and/or chlorido anion(s) as co-ligands; (iii) characterization of the compounds prepared by elemental analyses, spectroscopic methods (^1^H and ^13^C NMR for the Schiff base and Pd(ii) complex, EPR, IR, UV-vis for both the potential ligand and metal complexes), Electrospray Ionization Mass Spectrometry (ESI-MS) and single crystal X-ray diffraction (SC-XRD); (iv) determination of their thermodynamic solubility, speciation studies and evaluation of their stability in aqueous media; (v) investigation of their ability to directly reduce the tyrosyl radical (Y˙) in mouse R2 RNR (mR2) protein and inhibit tubulin polymerization; (vi) studies of their antiproliferative activity in the breast cancer cell line MCF-7 by fluorescence microscopy and the CellTiter-Glo luminescence cell viability assay, as well as the cell cycle; and finally (vii) obtaining insights into the underlying mechanism of antiproliferative activity supported by experimental evidence and molecular modeling.

In cell-free assays, our Cu(ii) complexes were found to efficiently inhibit both mouse R2 RNR and tubulin polymerization; however, when evaluated in MCF-7 cells, they predominantly manifest a phenotypic increase in the percentage of mitotic cells corresponding to tubulin inhibition. The apparent disconnect between cell-free mR2 RNR inhibition and the absence of a detectable S-phase arrest in MCF-7 cells cannot be resolved from the present single-time-point analysis. While the observed mitotic phenotype is consistent with tubulin inhibition, a time-dependent study would be required to determine whether RNR inhibition produces an earlier or transient perturbation of S-phase progression that is subsequently obscured by mitotic accumulation. We therefore consider the proposed kinetic relationship between the two activities a hypothesis rather than a demonstrated mechanism.

## Results and discussion

### Preliminary theoretical screening

**Fig. 1:**
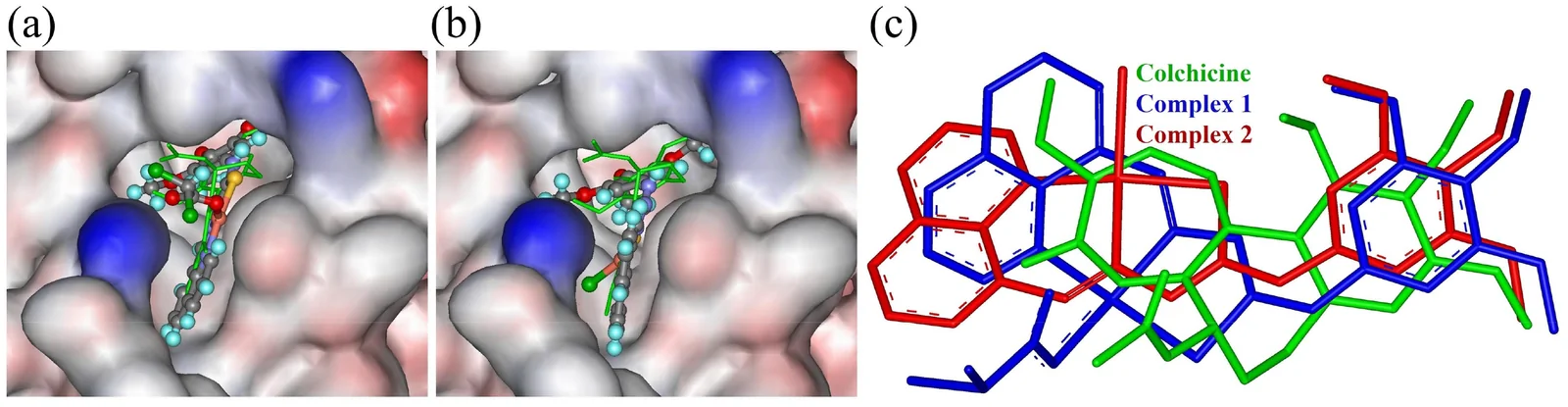
The docked poses of (a) complex 1 and (b) complex 2 (ball-and-stick plot or display style) in the colchicine binding site; the co-crystallized ligand LOC (colchicine) is shown in a capped stick style (green) and the H atoms are omitted for clarity. The protein surface is rendered; blue color depicts regions with a partial positive charge on the surface, red color indicates regions with a partial negative charge and gray shows neutral areas; (c) overlap of 1 and 2 with the colchicine molecule taken as a reference.

*In silico* screening was performed with a small library of twelve designed compounds (four quinoline-8-carboxaldehyde substituted *N*4-phenylthiosemicarbazones decorated with electron withdrawing and donating groups and eight Cu(ii) complexes (see Chart S1, SI)). The compounds were docked to the colchicine binding site of tubulin (see Fig. S2, SI) and to R2 protein (Fig. S1). The results are summarized in Fig. S3–S6 and Fig. S7–S10, respectively. Other modeling details can be found in the Experimental part (SI). The substitution pattern was considered favorable by evaluation of the binding scores for the compounds in Chart S1 and analysis of pharmacokinetic parameters (*e.g.*, aqueous solubility and lipophilicity) and other physicochemical properties related to Lipinski's rule of five, which are presented in Tables S1 and S2, respectively. Furthermore, we will describe in more detail the results related to tubulin, while those on R2 protein are described in the SI. The data show that the compounds fall within the optimal parameters, with the exception of some Cu(ii) complexes with DCA as a co-ligand, which slightly exceeds the relative molecular mass (*M*_r_) of 500. However, at physiological pH, the complexes may undergo aquation with replacement of the DCA co-ligand with a water molecule. Molecular docking calculations predicted good accommodation of the proligands in the protein pocket (Fig. S3, SI) without significant interactions with the functional groups of the amino acids, except by HL^OH^, which is involved in strong hydrogen bonding of the types N–H⋯O and O–H⋯O (Fig. S4); no interactions were found with the backbone of the enzyme. For Cu(ii) complexes, good accommodation in the protein pocket was also observed (Fig. S5) with a tentative coordination of ASN258 to Cu(ii) in complexes with DCA as a co-ligand (Fig. S6). We also noticed a good structural overlap between the molecular structures of compounds 1 and 2 and colchicine taken as a reference (Fig. 1).

**Fig. 2:**
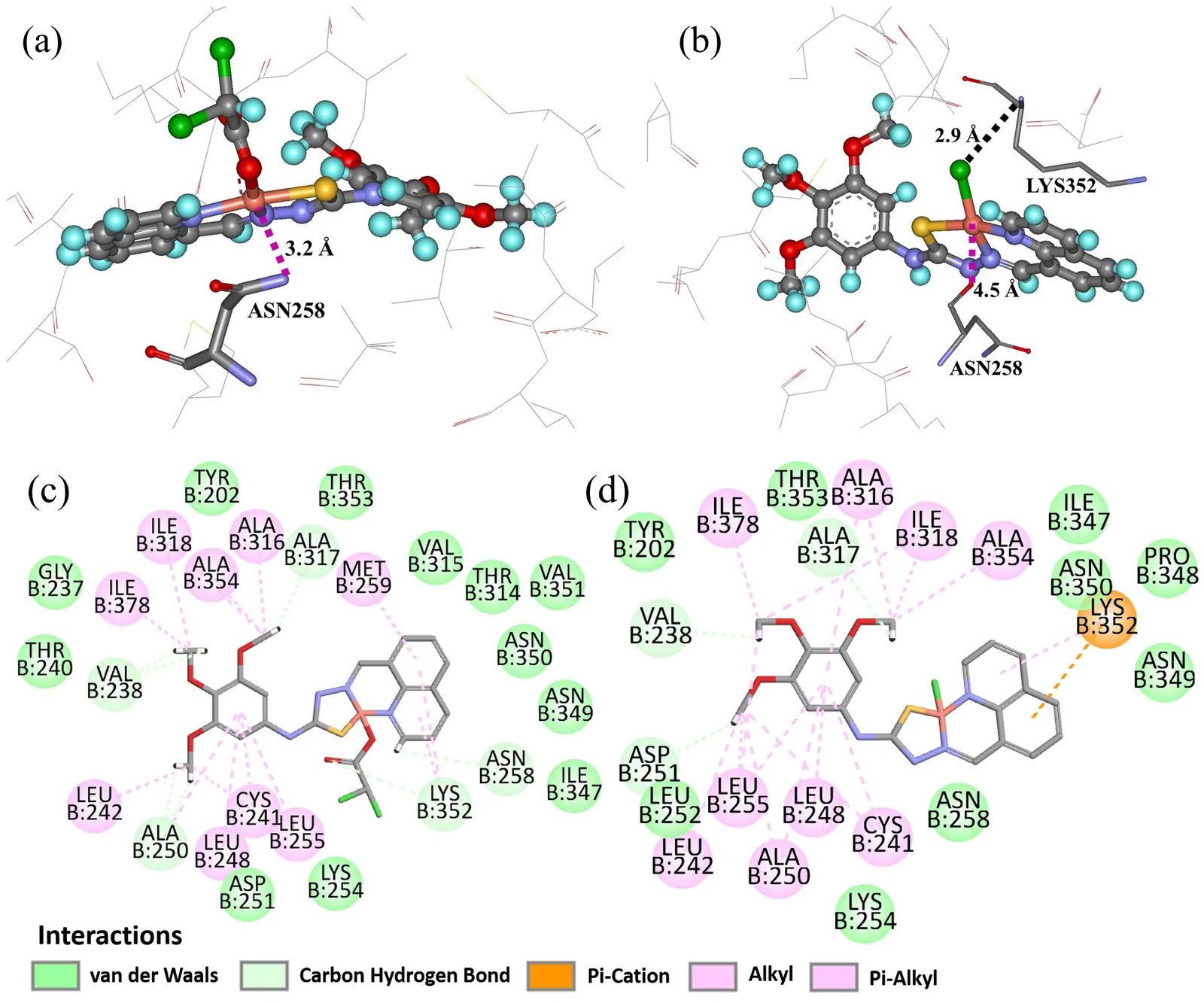
Predicted bindings for (a) complex 1 and (b) complex 2 (ball-and stick plot) with amino acids (capped stick plot) in the colchicine pocket. Potential contacts between ASN258 located within 5 Å and the central ion are represented as magenta dashed lines, H-bonding is shown as a black dashed line, while amino acids that do not establish interactions with Cu(ii) are displayed in a wire frame style. The 2D representation of all the intermolecular non-covalent interactions of the colchicine binding site with (c) complex 1 and with (d) complex 2. Green dashed lines represent hydrogen bonds. Orange and rosa dashed lines represent the π-cation and various types of hydrophobic interactions, respectively. Light green colored circles indicate the residues involved in van der Waals interactions.

Complex 1 is nestled inside the pocket, while the DCA co-ligand is oriented towards the aqueous phase creating premises for a hydrophilic–hydrophobic balance and stabilization of the molecule in the pocket. In contrast, complex 2 is predicted to be buried in the pocket due to the quinoline moiety directed towards the aqueous phase favoring pushing the molecule further inside the pocket. Of note are also the predicted short contact Cu⋯N (ASN258) of 3.2 Å (Fig. 2a) for 1, a markedly longer contact Cu⋯O (ASN258) of 4.5 Å and a H-bond interaction N–H⋯Cl between the LYS352 and the chlorido co-ligand for 2 (Fig. 2b). Apart from contacts Cu⋯N for 1 and Cu⋯O and H-bonding interaction N–H⋯Cl in 2, there is a large number of non-covalent intermolecular interactions between Cu(ii) complexes (1 and 2) and protein residues, which further contribute to their stabilization in the colchicine binding site. These are illustrated in Fig. 2c and d.

### Synthesis

**Chart 1:**
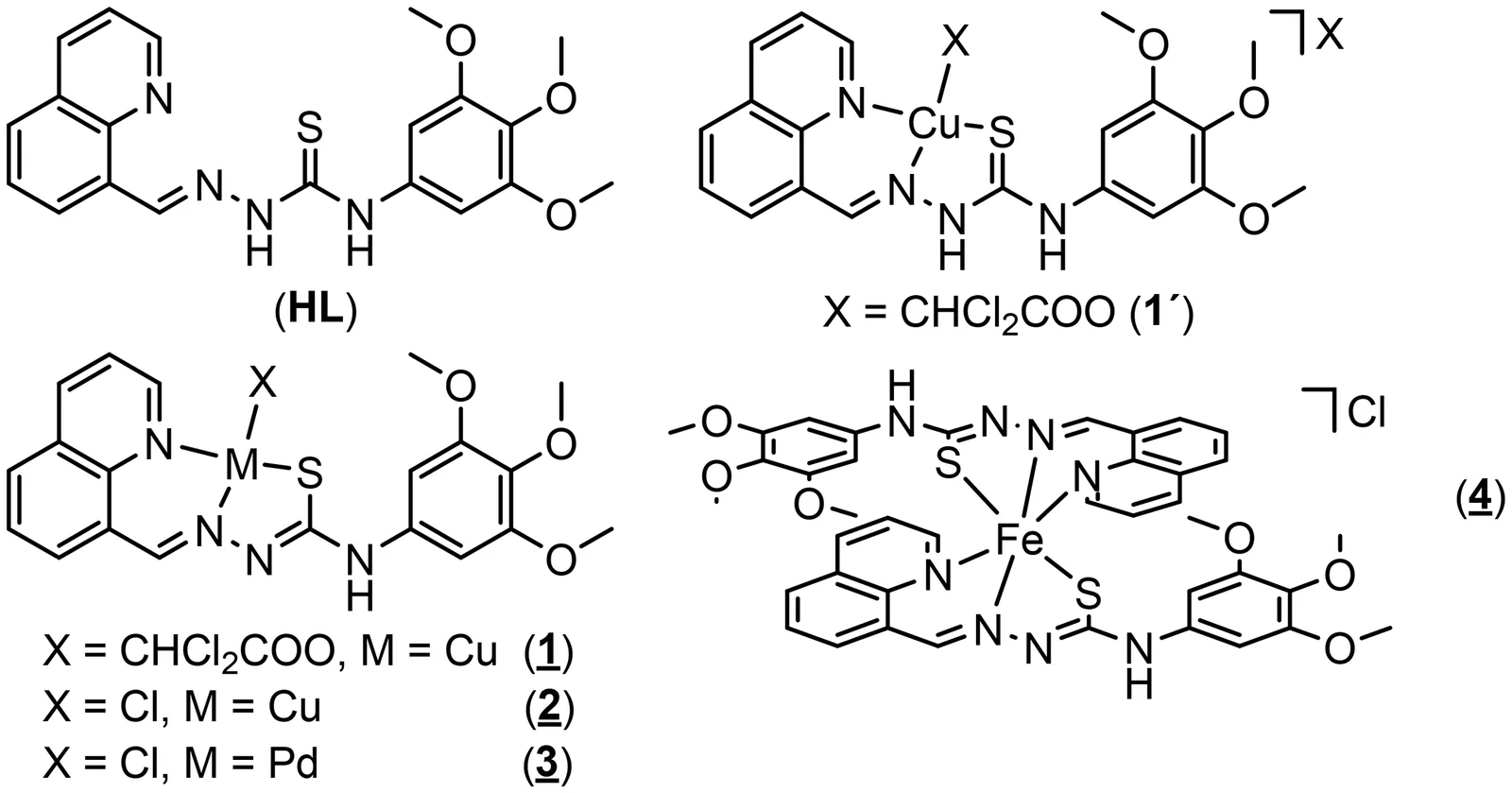
Line drawings of the compounds studied in this work. Underlined numbers indicate compounds studied by single crystal X-ray diffraction (SC-XRD).

Guided by the calculations performed and the predicted interactions, as well as the structural similarity between the colchicine molecule and complexes 1 and 2, we selected the Schiff base quinoline-8-carboxaldehyde *N*4-(3,4,5-trimethoxyphenyl)thiosemicarbazone (HL) and several metal complexes of 1 : 1 and 1 : 2 metal-to-ligand stoichiometry, including the favored complexes 1 and 2, as shown in Chart 1, for the synthesis. The 1 : 2 metal-to-ligand complexes have been included to probe the potential of coordination chemistry to create a roughly spherical and more rigid steric profile to better fit into the 3D colchicine pocket, as reported recently for Co(iii) and Fe(iii) complexes with other thiosemicarbazones,^33^ followed by analytical, spectroscopic, and structural characterization and biological evaluation.

The use of DCA as a co-ligand was prompted by the fact that dichloroacetic acid (HDCA) was reported to induce mitochondrial disfunction by inhibiting pyruvate dehydrogenase and does not have a toxic effect on normal cells.^44^ Recently, HDCA entered phase I clinical trials as a potential agent against brain tumors using oral administration.^45^ The antiproliferative activity of HDCA as a single drug in different types of cancer^46–50^ or in combination with clinically approved drugs^51–55^ is also well documented in the literature. A copper(ii) thiosemicarbazonate complex co-crystallized with DCA was found to be an effective antiproliferative agent against breast cancer cells.^56^

### Synthesis of the Schiff base HL

**Scheme 1:**
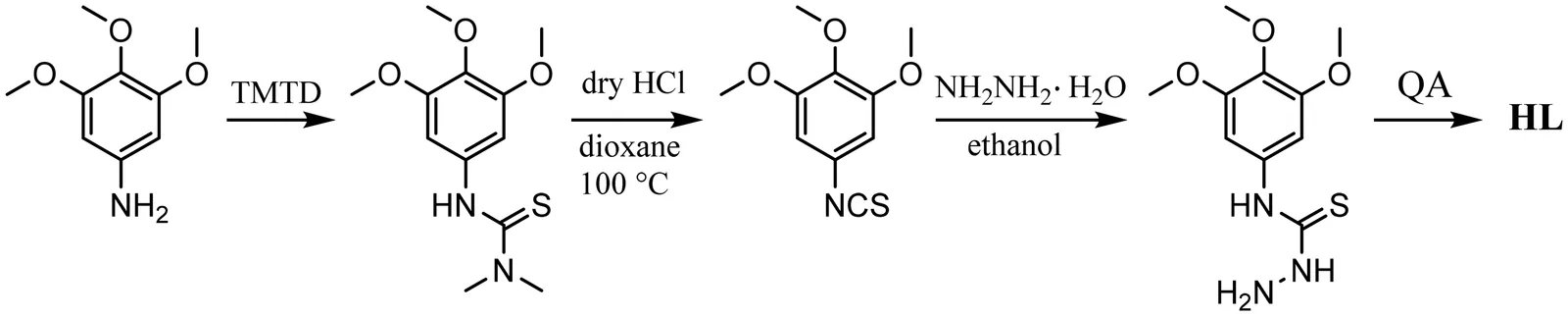
Synthetic route to HL. TMTD = tetramethylthiuram disulfide; QA = quinoline-8-carboxaldehyde.

The synthesis of quinoline-8-carboxaldehyde *N*4-(3,4,5-trimethoxyphenyl)thiosemicarbazone was performed by adapting a literature procedure,^57^ as shown in Scheme 1.

In the first step, the reaction of 3,4,5-trimethoxyaniline with tetramethylthiuram disulfide (TMTD) in DMF at 110 °C afforded *N*1-(3,4,5-trimethoxyphenyl)-*N*2-dimethylthiourea in 74% yield. Bubbling dry hydrogen chloride through a solution of this compound in dry dioxane at 100 °C resulted in the formation of *N*4-(3,4,5-trimethoxyphenyl)isothiocyanate in 78% yield. Further reaction with hydrazine hydrate in ethanol produced a white precipitate of *N*4-(3,4,5-trimethoxyphenyl)thiosemicarbazide in good yield. Finally, the condensation reaction of substituted thiosemicarbazide with quinoline-8-carboxaldehyde led to Schiff base HL in a close to quantitative yield. The formation of the desired thiosemicarbazone was confirmed by high resolution ESI(+) mass spectrometry (Fig. S11) and ^1^H and ^13^C NMR spectroscopy (Fig. S12 and S13). The peaks with *m*/*z* 397.1316 (calcd *m*/*z* 397.1329) and 419.1135 (calcd *m*/*z* 419.1154) were attributed to the ions [M + H^+^]^+^ and [M + Na^+^]^+^, respectively. The number of ^1^H and ^13^C resonances are in accordance with the *C*_1_ molecular symmetry of HL, while the CHNS elemental analyses are within ±0.4% and further confirm its analytical purity (≥95%).

### Synthesis of metal complexes

The reaction of an ethanolic solution of HL and 1 M HDCA with an added dropwise solution of Cu_2_(OH)_2_CO_3_ and 1 M HDCA produced green microcrystals of **[Cu(HL)(DCA)]DCA** (1′) in very good yield (92%). The high resolution ESI(+) mass spectrum of 1′ revealed peaks at *m*/*z* 458.0455 (calcd *m*/*z* 458.0468), 854.1699 (calcd *m*/*z* 854.1824) and 1045.0249 (calcd *m*/*z* 1045.0501), which could be assigned to [Cu^II^L]^+^, [Cu^II^L_2_ + H^+^]^+^ and [(Cu^II^L)_2_(CHCl_2_COO)]^+^, respectively (Fig. S14). Re-crystallization of 1′ in DMSO resulted in deprotonation of the Schiff base and generation of black-brown crystals of X-ray diffraction quality of **[Cu(L)(DCA)]·DMSO·0.5H**_**2**_**O (**1**·DMSO·0.5H**_**2**_**O)**. The composition of the bulk samples of these two complexes was established from elemental analysis, as was also the case for the other metal complexes reported herein. The high resolution ESI(+) mass spectrum of 1 (Fig. S15) showed a strong peak with *m*/*z* 458.0470 (calcd *m*/*z* 458.0468) assigned to [Cu^II^L]^+^. Two signals with higher *m*/*z* values of 1045.0272 (calcd *m*/*z* 1045.0501) and 1632.0078 (calcd *m*/*z* 1632.0429) were attributed to ions [(Cu^II^L)_2_(CHCl_2_COO)]^+^ and [(Cu^II^L)_3_(CHCl_2_COO)_2_]^+^, respectively. Similarly, the reaction of an ethanolic solution of HL and 1 M hydrochloric acid with an added dropwise solution of Cu_2_(OH)_2_CO_3_ and 1 M hydrochloric acid produced green microcrystals. These were re-crystallized in DMSO to give X-ray diffraction quality green crystals of **[Cu(L)Cl]·1.5DMSO·0.75H**_**2**_**O (**2**·1.5DMSO·0.75H**_**2**_**O)**. The ESI(+) mass spectrum of 2 (Fig. S16) showed a peak with *m*/*z* 458.0462 attributed to [Cu^II^L]^+^ (calcd *m*/*z* 458.0468). By reaction of a solution of HL in DMF with a solution of [PdCl_2_(MeCN)_2_] in acetonitrile in a 1 : 1 molar ratio, the Pd(ii) complex **[Pd(L)Cl]·H**_**2**_**O (**3**·H**_**2**_**O)** was prepared in 70% yield. The ESI(+) mass spectrum of 3 (Fig. S17 and S18) showed two peaks with *m*/*z* 542.09 attributed to [Pd^II^L(CH_3_CN)]^+^ (calcd *m*/*z* 542.05) and 1038.99 assigned to [(Pd^II^L)_2_Cl]^+^ (calcd *m*/*z* 1039.02). The formation of the Pd(ii) complex 3 was further confirmed by NMR spectroscopy. In the ^1^H NMR spectrum significant differences in chemical shifts are observed compared to the spectrum of the free ligand HL (see Fig. S19). Upon complex formation, the N3H group is deprotonated, and thus the N3H proton signal is no longer present in the spectrum. The resonances of the quinoline H1 proton and the imine proton H10 show significant shifts, with H1 being deshielded by 1.14 ppm and H10 shielded by 0.62 ppm. The binding to Pd(ii) also causes shifts of the remaining peaks in the spectrum, ranging from 0.5 ppm for the rest of the quinoline protons to 0.02 ppm for the least affected methoxy protons. The ^13^C NMR spectrum also shows shifts of all signals upon complex formation, with values ranging from 10.2 ppm for the amine carbon at position 10 to 0.1 ppm for the methoxy group carbons (see Fig. S13 and S20). The Fe(iii) complex **[Fe(L)**_**2**_**]Cl·3H**_**2**_**O·0.25DMF (**4**·3H**_**2**_**O·0.25DMF)** was synthesized in 65% yield from reaction of a solution of HL in DMF and an ethanolic solution of FeCl_3_ in a 2 : 1 molar ratio. The ESI(+) mass-spectrum of 4 (Fig. S21) showed a peak with *m*/*z* 846.19 attributed to [Fe^III^L_2_]^+^ (calcd *m*/*z* 846.17), while the ESI(−) mass spectrum (Fig. S22) revealed a peak with *m*/*z* 844.17 attributed to [Fe^III^(L − H^+^)_2_]^−^ (calcd *m*/*z* 844.16). The IR spectra of all compounds studied are shown in Fig. S23–S27. The CHNS elemental analyses for all reported metal complexes are within ±0.4%, except that for hydrogen found in complex 2, which is within ±0.52%.

### X-ray crystallography

**Fig. 3:**
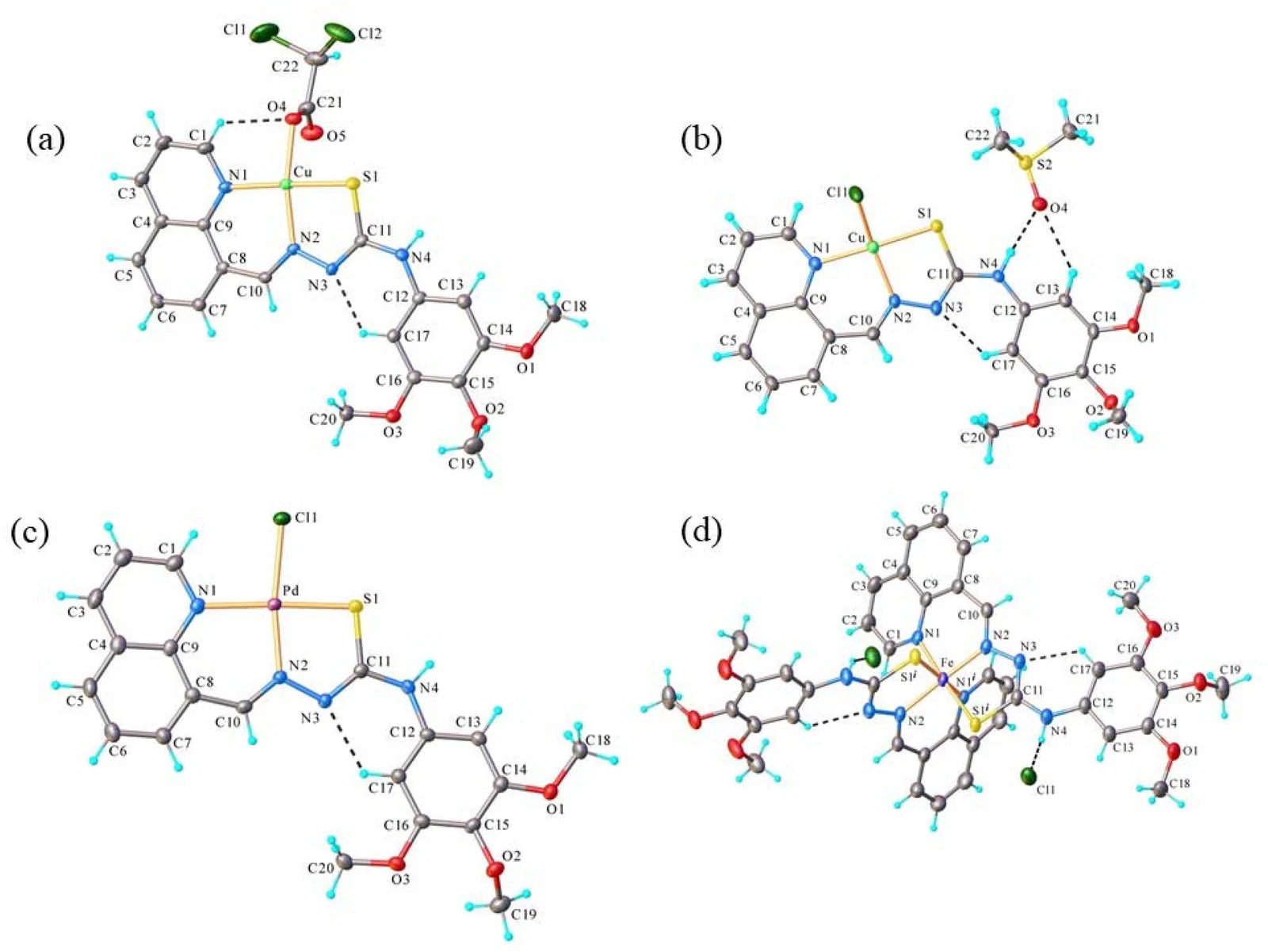
ORTEP views of complexes 1–4 with atom labeling schemes and thermal ellipsoids at the 50% probability level. Selected bond distances (Å) and bond angles (°) in (a) 1 (one of the two crystallographically independent molecules): Cu–N1 2.019(2), Cu–N2 1.969(2), Cu–S1 2.2449(7), Cu–O4 2.0002(18), N1–Cu–N2 93.12(8), N2–Cu–S1 85.14(6), N1–Cu–S1 177.34(6), N2–Cu–O4 165.84(8); in (b) 2: Cu–N1 2.024(2), Cu–N2 1.965(3), Cu–S1 2.2217(8), Cu–Cl1 2.2584(9), N1–Cu–N2 92.74(10), N2–Cu–S1 85.52(7), N1–Cu–S1 164.60(8), N2–Cu–Cl1 154.95(8); in (c) 3 (one of the two crystallographically independent molecules): Pd–N1 2.105(2), Pd–N2 1.990(2), Pd–S1 2.2337(7), Pd–Cl1 2.3489(7), N1–Pd–N2 92.83(9), N2–Pd–S1 84.61(7), N1–Pd–S1 177.21(7), N2–Pd–Cl1 170.46(7); and in (d) 4: Fe–N1 2.0608(18), Fe–N2 1.9468(18), Fe–S1 2.2055(6), N1–Fe–N2 91.78(7), N2–Fe–S1 85.68(5).

Complexes 1–4 have been further characterized by SC-XRD. Their structures are presented in Fig. 3 and details of data collection and refinement are given in Table S3. Selected bond distances and bond angles are quoted in the caption to the figure. Complexes 1–3 exhibit molecular crystal structures, while 4 is an ionic compound, where the asymmetric unit consists of one complex cation **[Fe(L)**_**2**_**]**^**+**^, one **Cl**^−^ as a counteranion, equally disordered between two symmetry-related positions, and one and a half severely disordered interstitial water molecules (Fig. 3d). The asymmetric unit in the crystals of 1**·DMSO·0.5H**_**2**_**O** and 3**·0.5H**_**2**_**O** contains two crystallographically independent, but chemically identical, complexes (see Fig. 3a and c, respectively). In both crystals, two independent complexes are linked into a dimeric associate *via* hydrogen bonds between the co-crystallized water molecule as a proton donor and oxygen atoms of the DCA co-ligand in 1 (Fig. S28) and the 1,2,3-trimethoxyphenyl group in 3 as proton acceptors (Fig. S29). The hydrogen bonding parameters are presented in Table S4. Compound 2**·DMSO** crystallized from dimethyl sulfoxide as a monosolvate. The co-crystallized molecule is associated with the complex through two hydrogen bonds N4–H⋯O4 and C13–H⋯O4 with a DMSO oxygen atom as a proton acceptor (Fig. 3b and Table S4). The essentially planar thiosemicarbazone base L^−^ exhibits the same coordination mode in all the complexes, acting as a tridentate ligand coordinated to the metal through the NNS set of donor atoms.

In 1–3, the distorted square-planar coordination geometry is completed by an oxygen atom of the DCA co-ligand in 1 and a chlorido co-ligand in 2 and 3 (Fig. 3). The distortion degree from the square-planar coordination geometry can be described with the *τ*_4_-parameter^58^ calculated using the equation 360 − (*α* + *β*)/141 = 0.119, 0.287 and 0.087 or the 
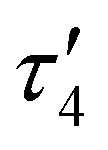
 -parameter^59^ calculated using the equation (*β* − *α*/360 − *θ*) + (180 − *β*/180 − *θ*) = 0.083, 0.255 and 0.066 for complexes 1–3, respectively, where *θ* is 109°, while *α* and *β* are the two largest valence angles about the central atom, and *β* > *α*. Both these parameters are equal to 0 for genuine square-planar complexes and to 1 for ideal tetrahedral metal complexes. In contrast, the Fe(iii) complex 4 is six-coordinate, with a distorted octahedral coordination geometry, which is formed by two tridentate monoanionic ligands L^−^ bound meridionally to the central atom (Fig. 3d).

### Electrochemistry and spectroelectrochemistry

As one of the mechanisms of R2 RNR inhibition implies reduction of Y˙ by potential reductants, the redox properties of HL and 1–4 were investigated by cyclic voltammetry (CV), EPR and UV-vis-NIR spectroelectrochemistry.

**Fig. 4:**
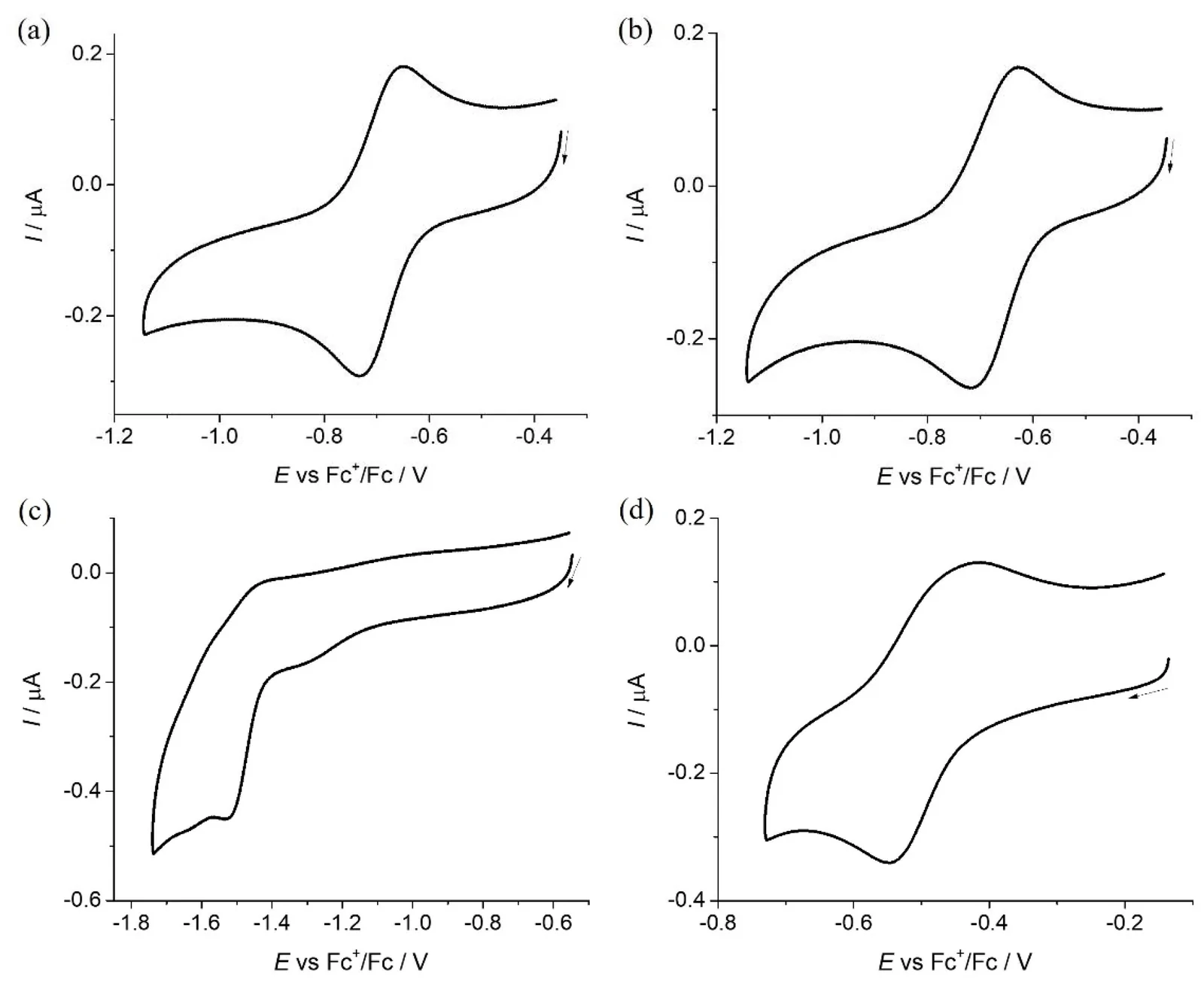
Cyclic voltammograms of 0.5 mM (a) 1, (b) 2, (c) 3 and (d) 4 and in DMSO/*n*-Bu_4_NPF_6_ at a scan rate of 100 mV s^−1^.

Almost reversible one-electron cathodic reduction at biologically accessible potentials (−0.4 to +0.8 V *vs.* NHE)^60,61^ was observed in CVs of Cu(ii) complexes in dimethyl sulfoxide (DMSO)/*n*-Bu_4_NPF_6_ using a platinum (Pt) working electrode at a scan rate of 100 mV s^−1^ (*E*_1/2_ = −0.69 V *vs.* Fc^+^/Fc for 1 [or −0.05 V *vs.* normal hydrogen electrode (NHE)] and *E*_1/2_ = −0.68 V *vs.* Fc^+^/Fc for 2 [or −0.04 V *vs.* NHE]), as shown in Fig. 4a and b.

**Fig. 5:**
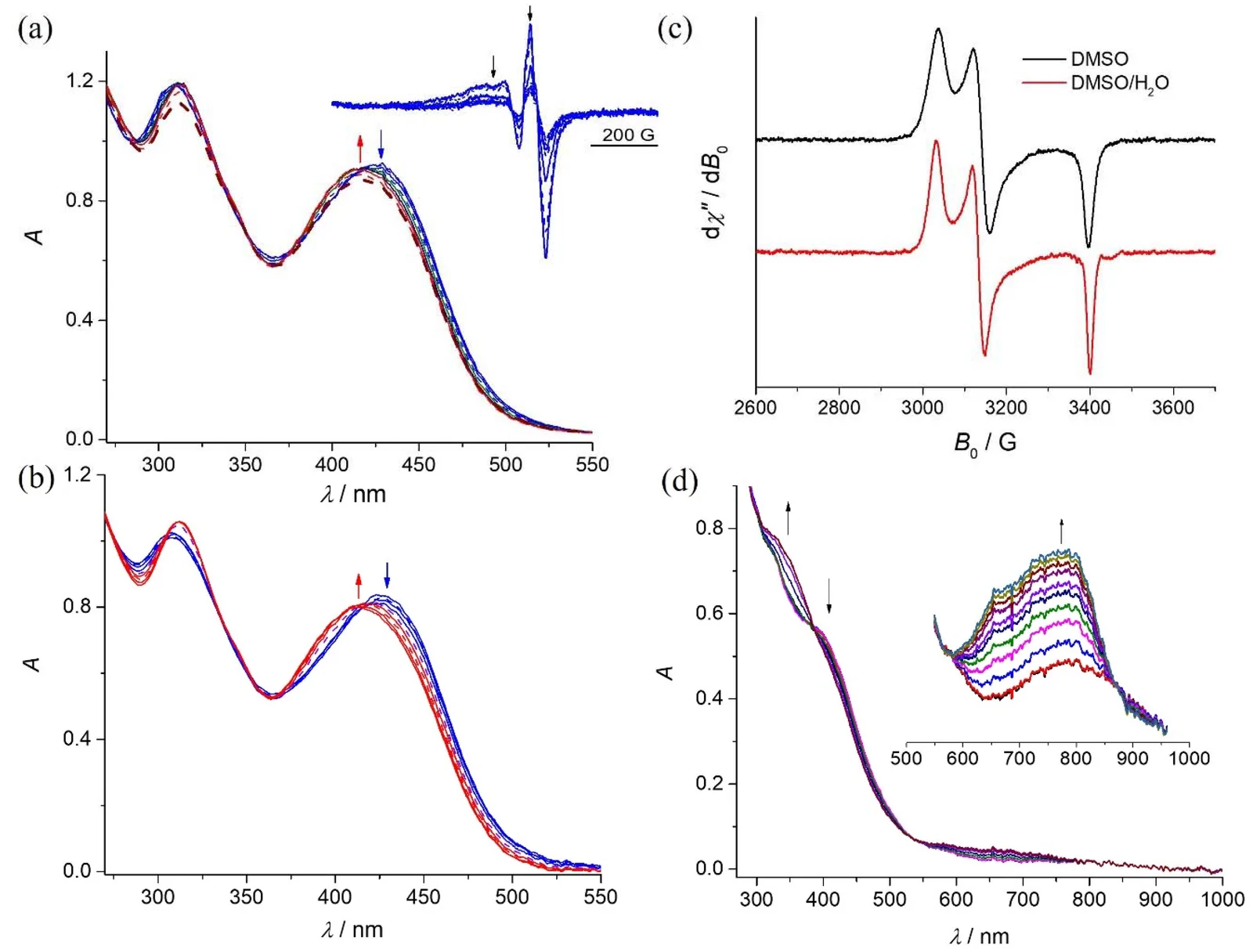
*In situ* UV-vis spectroelectrochemistry for (a) 1 (inset: changes in EPR spectra of 1 in DMSO/*n*-Bu_4_NPF_6_ measured at the first reduction peak using a Pt mesh working electrode) and (b) 2 in DMSO/*n*-Bu_4_NPF_6_ (scan rate 5 mV s^−1^); (c) X-band EPR spectra of 4 in DMSO and in DMSO/H_2_O glass at 105 K; (d) UV-vis-NIR spectroelectrochemistry of 4 in DMSO/*n*-Bu_4_NPF_6_ for the Fe(iii)/Fe(ii) redox couple.

Note that HL is not redox-active in both the cathodic and the anodic regions at biologically accessible potentials (black trace in Fig. S30a). No oxidation processes were observed in the anodic region for 1 (blue trace in Fig. S30a) and 2 (CV not shown). The quasireversible one-electron reduction of 1 and 2 was further studied by *in situ* UV-vis spectroelectrochemistry (Fig. 5a and b). Upon cathodic reduction at the first reduction peak, an isosbestic point at 435 nm (for both 1 and 2) was observed. The changes observed in the S → Cu(ii) charge transfer (CT) bands (∼425 nm) clearly confirmed the reduction of Cu(ii) to Cu(i). Additionally, upon voltammetric reverse scan, reoxidation and a nearly full recovery of the initial absorption bands were observed, attesting the chemical reversibility of the cathodic reduction even at low scan rates (Fig. S31a and b).

Even though a reversible behavior was observed for 1 upon reduction (Fig. S31a), the band attributed to the Cu(i) complex at 414 nm decreased with time of reduction, indicating precipitation of poorly soluble Cu(i) species (see the dashed line trace in Fig. 5a). The involvement of Cu(ii) in the reduction processes was confirmed by *in situ* EPR spectroelectrochemistry of 1 and 2 in *n*-Bu_4_NPF_6_/DMSO. As can be seen for electrochemical reduction of 1 in the region of the first one-electron reduction step, a significant decrease of the EPR signal is in accordance with the formation of a diamagnetic Cu(i) (d^10^, *S* = 0) complex (inset in Fig. 5a). Similar behavior was found for 2 (Fig. S30b).

The reversibility of the reduction Cu(ii)/Cu(i) was significantly affected for DMSO/aqueous solutions of the complexes, implying a more intricate redox mechanism involving the release of the proligand HL as illustrated for 1 in Fig. S30a (red trace). The electrochemical reduction of Pd(ii) complex 3 is fully irreversible (Fig. 4c) and irreversible changes were also observed in UV-vis spectra upon reduction (Fig. S31c). The low-spin electronic configuration of Fe(iii) in 4 (d^5^, *S* = ½) was confirmed by LN_2_ EPR spectroscopy (Fig. 5c). The principal *g*-values [*g*_1_, *g*_2_, *g*_3_] are equal to [1.982, 2.151, 2.224] for this compound in frozen DMSO/H_2_O solution and in accordance with those documented for other low-spin Fe(iii) complexes of thiosemicarbazones.^33^ A quasireversible reduction of 4 in DMSO corresponding to the Fe(iii)/Fe(ii) redox couple (*E*_1/2_ = −0.55 V *vs.* Fc^+^/Fc, [or +0.09 V *vs.* NHE]) is shown in Fig. 4d, while the accompanying changes in UV-vis-NIR spectra are shown in Fig. 5d. A broad absorption band between 600 and 900 nm developed after cathodic reduction of 4 in DMSO/*n*-Bu_4_NPF_6_. Appearance of similar spectral features was reported for other Fe(iii)–TSC complexes.^33^ Additionally, upon voltammetric reverse scan, reoxidation and nearly full recovery of the initial optical bands were observed. However, some difference in behavior deserves to be mentioned. Even though an almost complete recovery of the initial optical bands of the original Fe(iii) complex 4 upon back reoxidation was achieved, we noticed partial release of the proligand upon the cathodic reduction at low scan rates of 5 mV s^−1^ (Fig. S30c, d and S31d). Given the redox activity of 1, 2 and 4, implication of these complexes in generation of reactive oxygen species (ROS) in cancer cells cannot be excluded.

### ROS generation

**Fig. 6:**
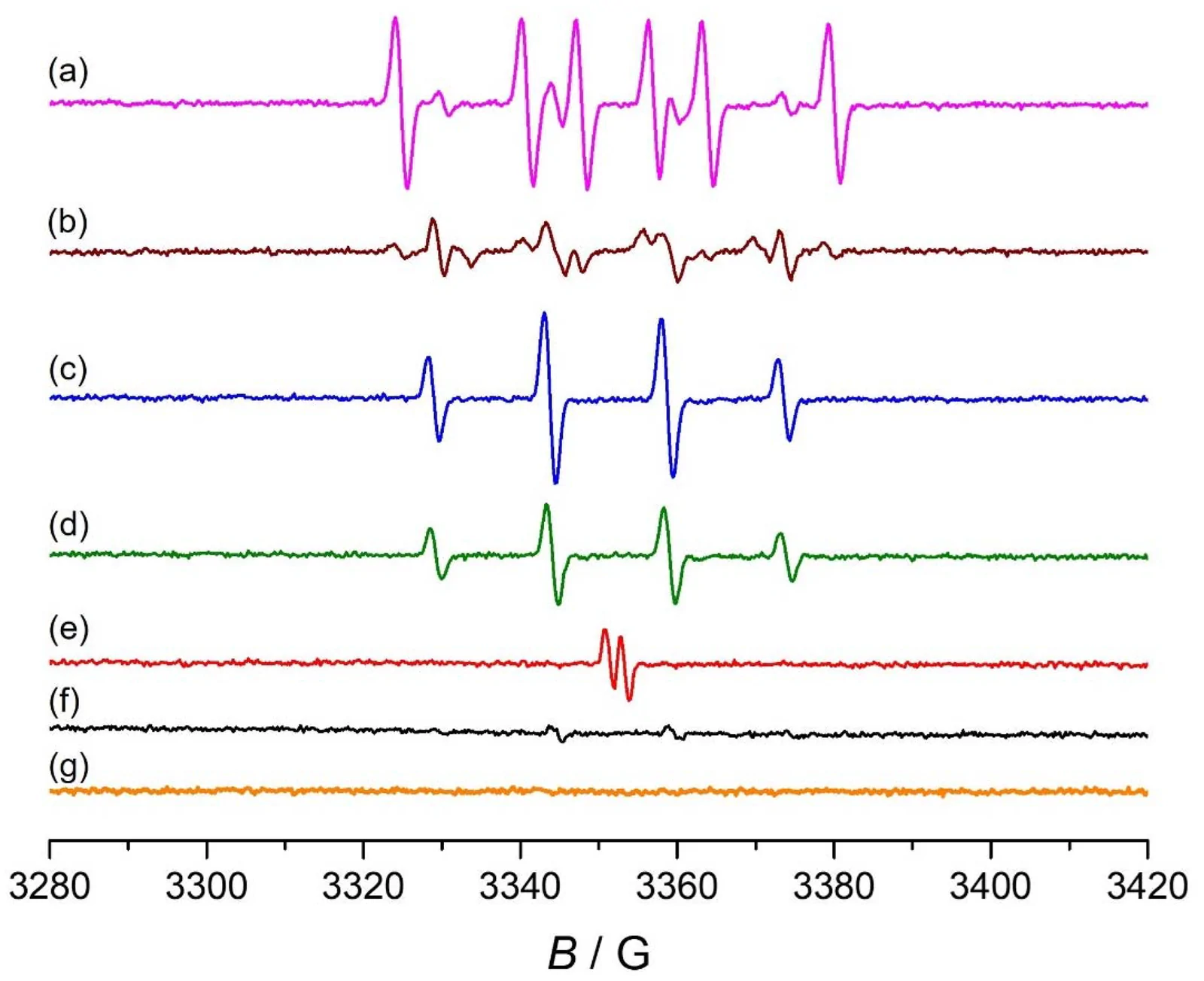
Experimental EPR spectra measured for the systems: (a) 2/DMPO/Asc/H_2_O_2_ in air in a 30% (v/v) DMSO/H_2_O mixture (magenta trace), (b) 4/DMPO/Asc/H_2_O_2_ in air in a 30% (v/v) DMSO/H_2_O mixture (brown trace), (c) 2/DMPO/Asc/H_2_O_2_ in air in water (blue trace), (d) 1/DMPO/Asc/H_2_O_2_ in air in water (green trace), (e) 2/DMPO/Asc in air in water (red trace), (f) 2/DMPO in air in water (black trace), and (g) for the reference system solvent + DMPO (orange trace). Initial concentrations: *c*(1, 2, 4) = 0.08 mM, *c*(DMPO) = 0.04 M, *c*(Asc) = 0.4 mM, and *c*(H_2_O_2_) = 0.1 M.

The ability of compounds 1, 2 and 4 to generate ROS was investigated in cell-free settings by the EPR spin trapping technique. First, we investigated the reactions of 1 and 2 in a 30% (v/v) DMSO/H_2_O mixture (0.08 mM) with ascorbate (Asc) as a natural reducing agent and H_2_O_2_ in the presence of the DMPO spin-trapping agent under air. As seen in Fig. 6a (magenta trace), complex 2 induced strong ROS generation after addition of Asc and H_2_O_2_ along with reactive radical intermediates evidenced by the presence of the DMPO spin adducts.

The dominating six-line EPR signal indicates the formation of carbon centered radicals ·DMPO–CH_3_ originating from reactions of ROS with DMSO. The Fe(iii) complex 4 also produces ROS (Fig. 6b, brown trace), but less efficiently than the Cu(ii) complexes 1 and 2 using the same concentration of the samples. This is at least in part due to its low solubility in water, as expected for complexes with a high molecular mass. The four-line EPR signal observed for 2 is due to the ·DMPO–OH spin adduct,^62^ which fully dominates in an aqueous environment (Fig. 6c, blue trace). The Cu(ii) complex 2 was more effective in producing hydroxyl radicals than 1 in aqueous solution (Fig. 6d, green trace). The red trace (Fig. 6e) represents the EPR spectrum measured immediately after addition of an excess (5 equiv.) of ascorbate to the solution of 2 in the presence of DMPO and can be ascribed to the ascorbyl radical, which is formed by the reduction of 2 with Asc.^63^ No EPR signal of the ascorbyl radical was found in the absence of 2. Only minor amounts of ROS were generated in the absence of Asc and H_2_O_2_ (Fig. 6f, black trace). No EPR signal was observed for the reference system solvent + DMPO (Fig. 6g, orange trace). To confirm that Cu(ii) complexes 1 and 2 significantly increase the formation of ROS in aqueous solutions, a series of experiments was performed with different reference systems (controls): 1. water + DMPO; 2. water + DMPO + ascorbate; 3. water + DMPO + H_2_O_2_; 4. water + DMPO + ascorbate + H_2_O_2_ and buffer HEPES + DMPO + ascorbate + H_2_O_2_. The results are shown in Fig. S32. Only minor amounts of radicals were observed for the control system H_2_O/DMPO/Asc/H_2_O_2_ (magenta trace in Fig. S32) in contrast to the strong signal of the ·DMPO–OH spin adduct in the presence of 2 (violet trace in Fig. S32). Given that hydrogen peroxide is a strong oxidant, a partial oxidation of ascorbate to the ascorbyl radical in the absence of metal complexes but in the presence of H_2_O_2_ was expected indeed (magenta and green traces in Fig. S32). However, the amounts of ascorbyl radicals observed in the presence of 2, but in the absence of H_2_O_2_, were markedly higher, confirming the substantial reduction of 2 by ascorbate. Of note is also that the formation of spin adducts in the presence of HL or Pd(ii) complex 3 was not seen.

To provide more compelling evidence that ROS generation in the presence of 1 and 2 is due to their interaction with biochemical reductants, *e.g.*, dithiothreitol (DTT), with a standard redox potential of −0.33 V,^64^ and natural reducing agents, such as Asc (standard redox potential −0.282 V),^65^ NADH (standard redox potential −0.32 V),^66^ and GSH (standard redox potential −0.24 V),^67^ further studies were performed by UV-vis spectroscopy in a 1.3% (v/v) DMSO/H_2_O mixture.

After mixing complex 2 in a 1.3% (v/v) DMSO/H_2_O mixture and DTT, a significant decrease in *A*_400_ was observed (note that in pure DMSO this band is shifted to 425 nm), while *A*_345_ of the metal-free ligand increased. These changes imply that, after reduction in the presence of excess DTT, the generated Cu(i) complex is not stable in an aqueous environment and liberates the free ligand. This is in line with the voltammetric studies in aqueous solutions. Upon purging the solution with air, the Cu(ii) complex was regenerated (Fig. S33a), suggesting a reversible redox process. Substantial changes in the UV-vis spectrum of 2 occurred after addition of 5 equiv. of Asc, as shown in Fig. S33b, providing further evidence for the reaction of natural reductants with 2. The first recorded spectrum after mixing complex 2 and Asc showed a marked shift of the absorbance band at 400 nm to 464 nm (Fig. S33b). This band then continuously decreased with the reaction time and shifted to 453 nm, while the baseline increased. This indicates the formation of less soluble Cu(i) species, as already observed in voltammetric studies. In pure DMSO, the band at 425 nm substantially decreased after the addition of Asc and the maximum shifted to 414 nm (Fig. S33c) as observed in the spectroelectrochemical experiments in pure DMSO (see Fig. 5b). After addition of GSH to 2 in a 1.3% (v/v) DMSO/H_2_O mixture, a substantial decrease of the band at 400 nm was seen, which again increased with time, suggesting reversible redox processes (Fig. S33d). Analogous behavior was disclosed for Cu(ii) complex 1 (not shown). Negligible changes in the original spectra of 1 and 2 in a 1.3% (v/v) DMSO/H_2_O mixture were observed after addition of NADH (not shown).

### Solution phase studies indicate the poor aqueous solubility of the Schiff base, which is increased upon complex formation with Cu(ii), while it remained low when coordinated to Pd(ii) or Fe(iii)

The thermodynamic solubility (*S*) of HL and complexes 1–4 in water was determined by UV-vis spectroscopy and the data are summarized in Table 1. The collected *S* values clearly indicate a huge increase of aqueous solubility of the proligand upon complex formation with Cu(ii) (complexes 1 and 2), while the solubility of the Pd(ii) and Fe(iii) complexes 3 and 4 increased only slightly when compared to that of HL. The significantly enhanced aqueous solubility of the Cu(ii) complexes can be rationalized by their elevated lability, which allows for partial ligand exchange (most likely replacement of co-ligands (DCA, Cl^−^) by water), which leads to the formation of positively charged species and thus improved hydrophilicity. In contrast, the Pd(ii) and Fe(iii) complexes predominantly exist as neutral species with a more inert coordination environment for Pd(ii) and more stable Fe(iii), resulting in only minor changes in the coordination sphere, and in the solubility compared to the free ligand.

**Table 1:** Thermodynamic solubility (*S*/µM) in water and logarithmic distribution coefficients (log *D*_7.4_) of HL and metal complexes 1–4

	HL	1	2	3	4
*S*/μM	≤1	125.6	98.7	≤1	≤1
log *D*_7.4_	>+2.5	+0.72 ± 0.02	+0.76 ± 0.03	+1.96 ± 0.01	—a

a Not soluble in either phase.

The logarithmic distribution coefficients (log *D*_7.4_) were determined by the shake-flask method at pH 7.4 in an *n*-octanol/water partition study, as presented in Table 1. HL and complex 3 are highly lipophilic, and this correlates with their low aqueous solubility. Complexes 1 and 2 are more hydrophilic than HL, resulting in moderate aqueous solubility. Due to the negligible solubility of 4 in both phases, the partition coefficient for this complex could not be determined.

### Solution equilibrium studies: proton dissociation of HL and its complex formation with Fe(iii) and Cu(ii)

**Fig. 7:**
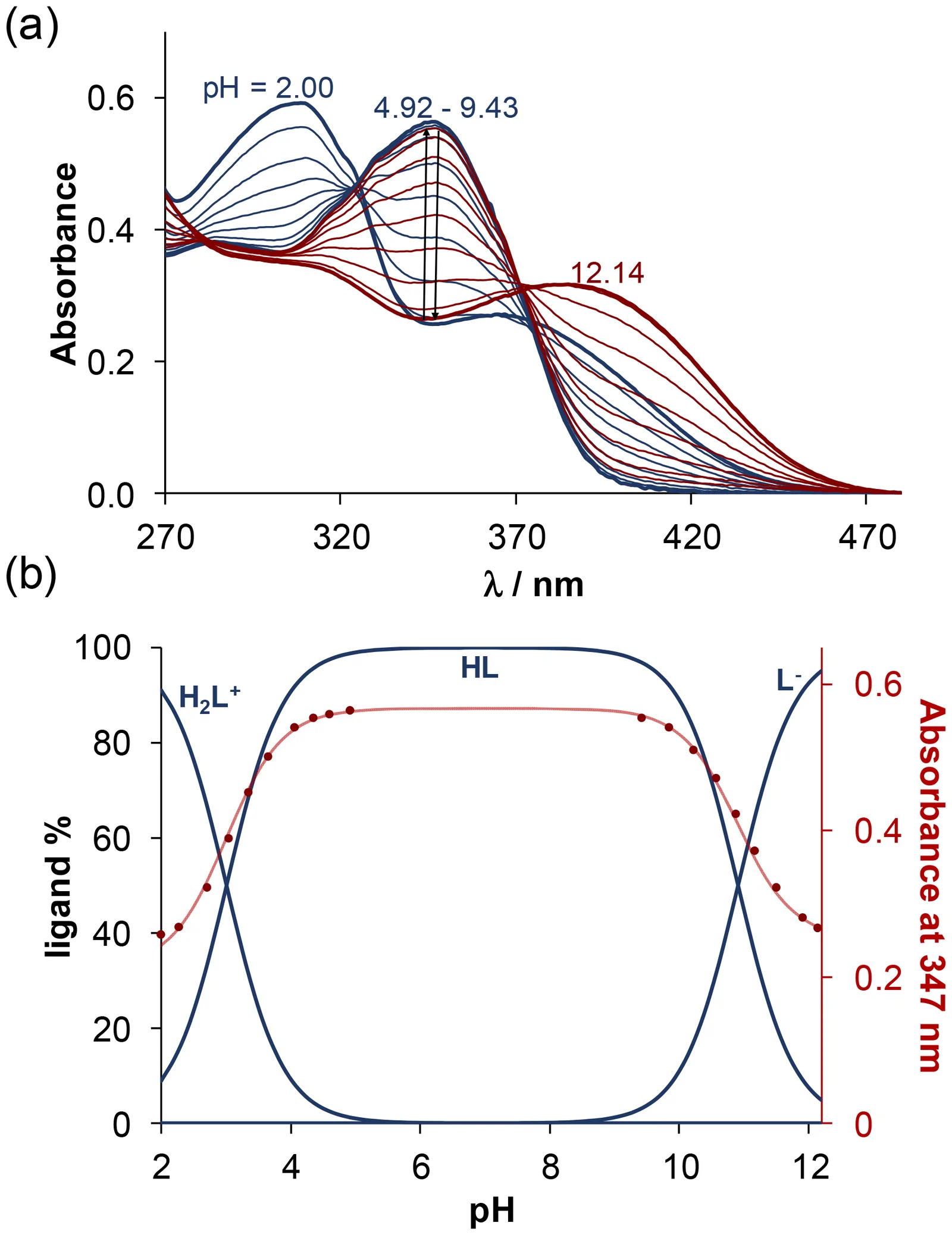
(a) UV-vis spectra of HL recorded in the pH range 2.0–12.1; (b) concentration distribution curves calculated with the determined p*K*_a_ values and *A*_347_ values (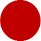) with the fitted line (red solid line); {*c* = 30 μM; 30% (v/v) DMSO/H_2_O; *I* = 0.10 M KCl; *l* = 1 cm; *T* = 25.0 °C}.

Prior to the equilibrium studies, the stability of the compounds was evaluated in an aqueous solution (in 2% (v/v) DMSO/H_2_O for complexes 1–4 and 50% (v/v) DMSO/H_2_O for HL) at pH 7.4 for 24 h (Fig. S34). Slow transformation with no significant changes within the first 2 h was observed for HL and complexes 1, 2, and 4, while complex 3 proved to be stable. Due to the limited aqueous solubility of HL, its proton dissociation constants were determined by UV-vis spectrophotometric titration in a 30% (v/v) DMSO/H_2_O mixture at a low concentration (30 µM). This solvent mixture is a standard used for related TSCs^55^ with similar solubility. The fully protonated form of HL possesses two dissociable groups, namely the quinolinium nitrogen (NH^+^) and the hydrazone nitrogen (NH). Two processes were observed in the spectra (Fig. 7a) over the studied pH range (2–12). The lower p*K*_a_ value is assigned to the quinolinium nitrogen, while the higher p*K*_a_ value is attributed to the hydrazone nitrogen. Based on the obtained p*K*_a_ values (Table 2), concentration distribution curves were computed (Fig. 7b), revealing that the neutral HL form predominates over a wide pH range, including the physiological pH 7.4, as previously observed for related α-*N*-pyridyl TSCs.^68^

**Table 2:** Overall protonation constants (log *β*) and p*K*_a_ values of HL determined by UV-vis titrations in 30% (v/v) DMSO/H_2_O in addition to the *λ*_max_ and molar absorbance (*ε*) values of the ligand species in different protonation states; {*I* = 0.1 M KCl; *T* = 25.0 °C}

	H_2_L^+^	HL	L^−^
log *β*	13.92 ± 0.15	10.91 ± 0.09	
p*K*_a_	3.01	10.91	
*λ*_max_ (nm)/*ε* (M^−1^ cm^−1^)	308/19599	347/18 706	385/19 436

**Fig. 8:**
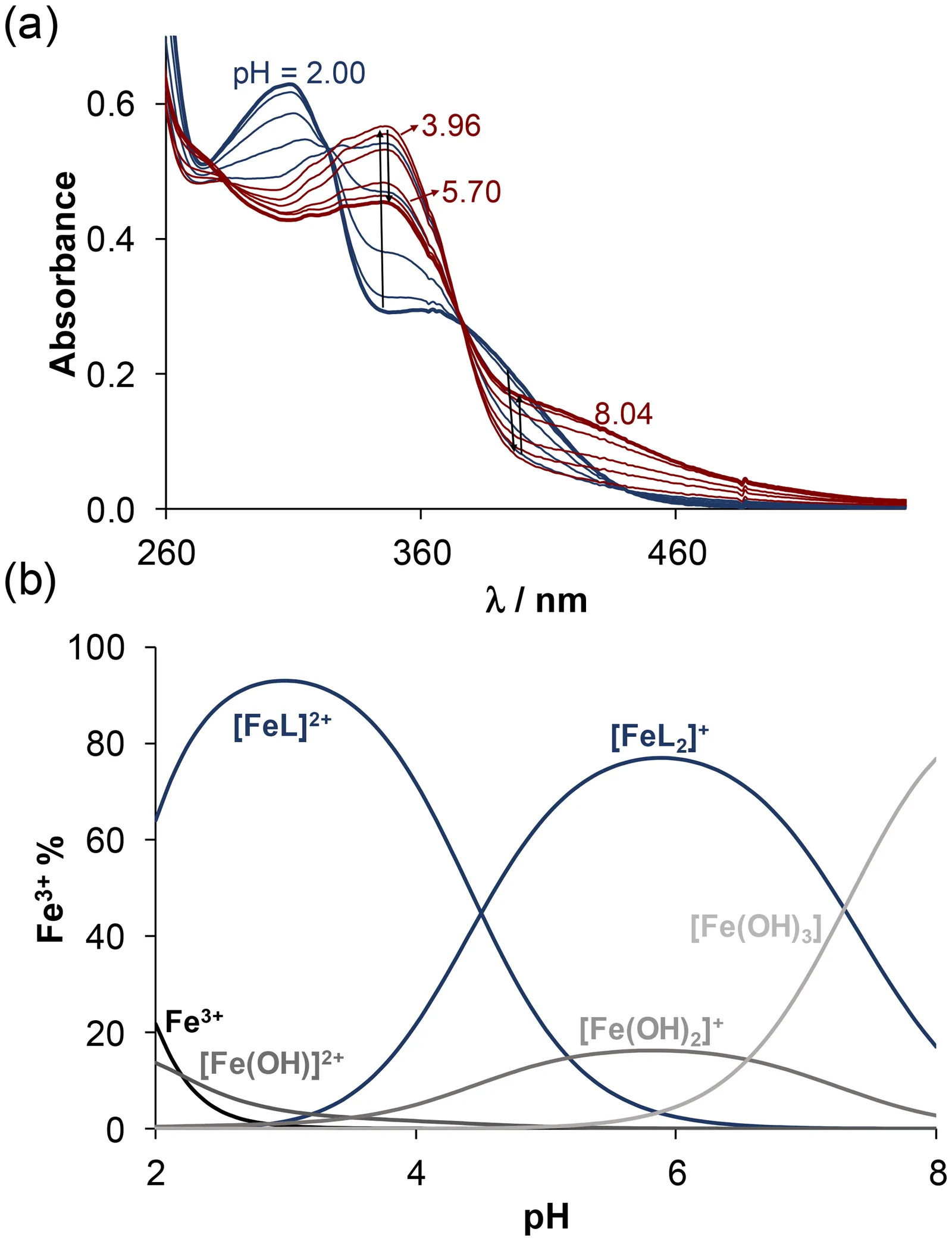
(a) UV-vis spectra recorded for the Fe(iii)–HL (1 : 2) system in the pH range 2.0–8.0; (b) concentration distribution curves calculated for the same system on the basis of the determined equilibrium constants. {*c*_HL_ = 32 μM; *c*_Fe(III)_ = 16 μM; 30% (v/v) DMSO/H_2_O; *I* = 0.10 M KCl; *l* = 1 cm; *T* = 25.0 °C}.

UV-vis spectrophotometric titrations were performed to monitor Fe(iii) complex formation of HL in 30% (v/v) DMSO/H_2_O. Representative spectra from the spectrophotometric titrations are shown in Fig. 8a for the Fe(iii)–HL (1 : 2) system. The spectra could be evaluated only up to pH 8.0. Above this pH, a new band appeared at *λ*_max_ = 750 nm, which is characteristic of Fe(ii) complexes,^69^ suggesting a redox process. Furthermore, the hydrolysis of Fe(iii) also becomes significant above this pH. Based on the absorption spectra, the formation of mono- and bis-ligand complexes ([FeL]^2+^ and [FeL_2_]^+^) was identified, similarly to related α-*N*-pyridyl TSCs.^69^ The determined overall stability constants are as follows: log *β*([FeL]^2+^) = 15.45 ± 0.05, log *β*([FeL_2_]^+^) = 26.93 ± 0.08. The calculated concentration distribution curves (Fig. 8b) at physiological pH (7.4) indicate that the bis-ligand complex is the predominant species. However, it is important to note that significant hydrolysis already occurs at this pH under the applied conditions (∼60%). To ensure comparability with related compounds, predominance curves were calculated for the Fe(iii)–ligand 1–ligand 2 hypothetic systems (at a 1 : 2 : 2 metal-to-ligand-to-ligand ratio) where HL (as ligand 1) paired with FTSC (Fig. S35a) and triapine^69^ (both as ligand 2) (Fig. S35b). At physiological pH, the bis-ligand complex predominates in all cases. The results demonstrate that HL, featuring a 6,5 chelate ring size sequence in an Fe(iii) complex, exhibits a greater affinity for this metal ion than TSCs, such as FTSC and triapine, which form complexes with a 5,5 ring size sequence.

**Fig. 9:**
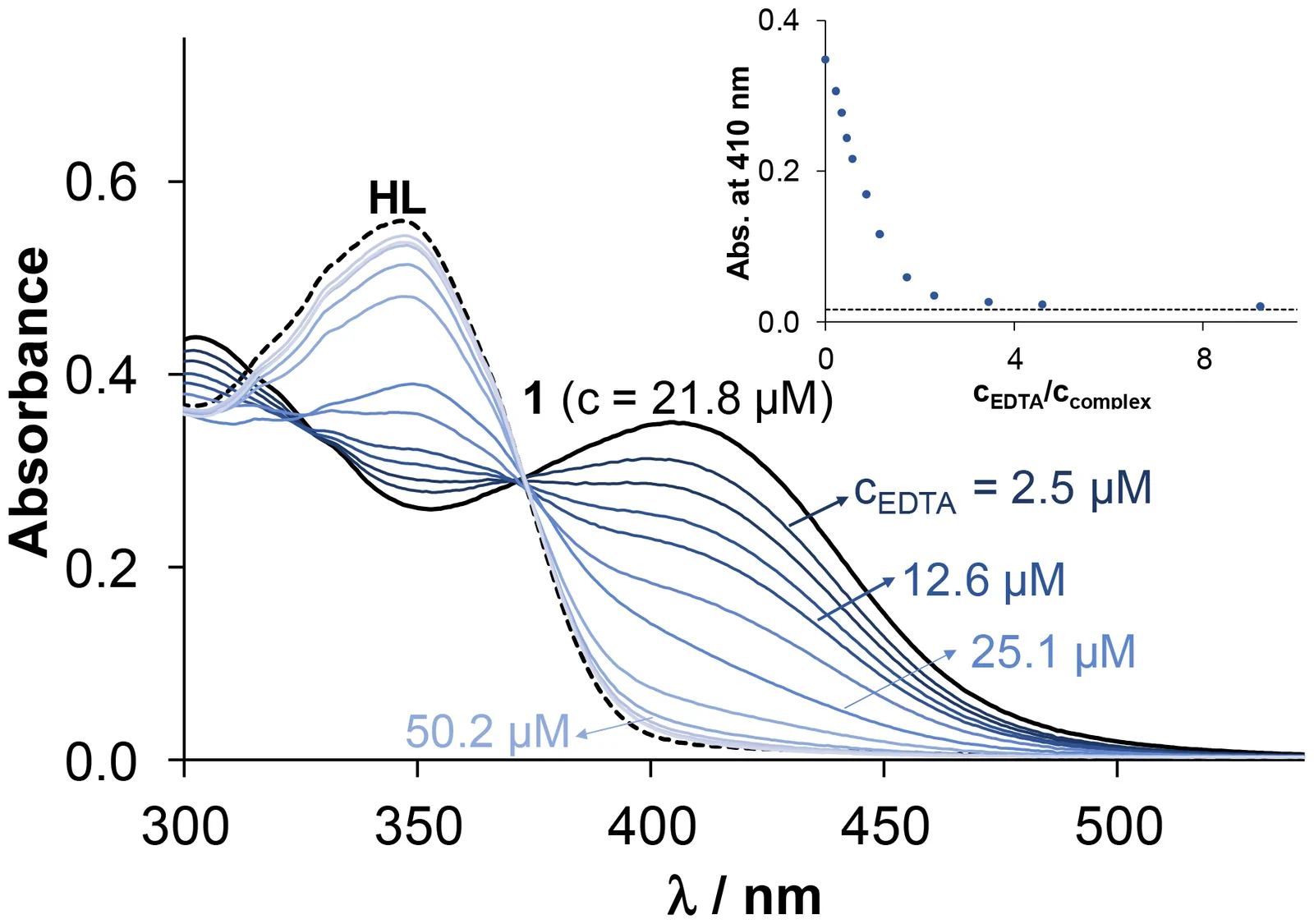
UV-vis spectra of complex 1 in the presence of EDTA at various concentrations. Inserted figure shows the absorbance of the samples at 410 nm as a function of the EDTA excess; {*c*_1_ = 21.8 µM; *c*_EDTA_ = 0–200 µM; pH = 5.90 (50 mM MES); 30% (v/v) DMSO/H_2_O; *I* = 0.10 M (KCl); *l* = 1 cm; *T* = 25.0 °C}.

For related α-*N*-pyridyl TSCs,^55^ the highly stable [CuL]^+^ complex predominates over a wide pH range between 3.5 and 8 at a 1 : 1 metal-to-ligand ratio. Consequently, conditional stability constants are often determined *via* competition experiments with EDTA^70^ at pH 5.9. In the present study, the stability of complexes 1 and 2 was investigated using similar competitive reactions. The measurements were performed at pH 5.9, as the [CuL]^+^ complex is the only species present in the solution under these conditions. A 30% (v/v) DMSO/H_2_O mixture was used to prevent precipitation of the displaced ligand, which is practically insoluble in water. Based on the time-dependent measurements shown in Fig. S36, a 6 h equilibration period was allowed to ensure the reaction was complete. A representative series of spectra for complex 1 is shown in Fig. 9. For the calculation of the conditional stability constants, absorbance data above 370 nm were used, as only the TSC complex and the free TSC ligand exhibit absorption in this region. The conditional stability constants at pH 5.9 
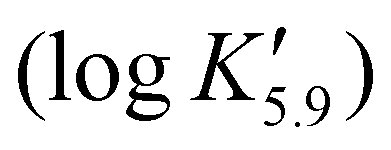
 were determined to be 10.82 ± 0.04 for complex 1 and 10.68 ± 0.07 for complex 2. For comparison, the conditional stability constants for triapine and FTSC^68,71^ were calculated using their p*K*_a_ values and the stability constants (log *β*) of their [CuL]^+^ complexes, as determined in a 30% (v/v) DMSO/H_2_O solvent mixture. The 6,5 chelate ring size sequence enhanced the stability of the Cu(ii) complexes of HL compared to triapine 
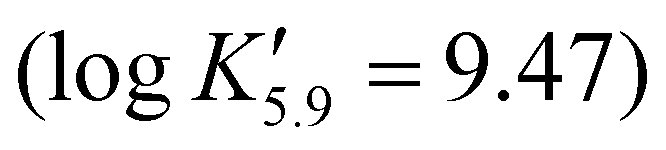
 and FTSC 
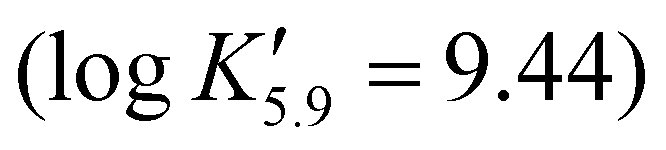
 in DMSO/H_2_O, both with a 5,5 chelate ring sequence.

### Interaction of HL and 1–4 with mR2 RNR in cell-free settings

**Fig. 10:**
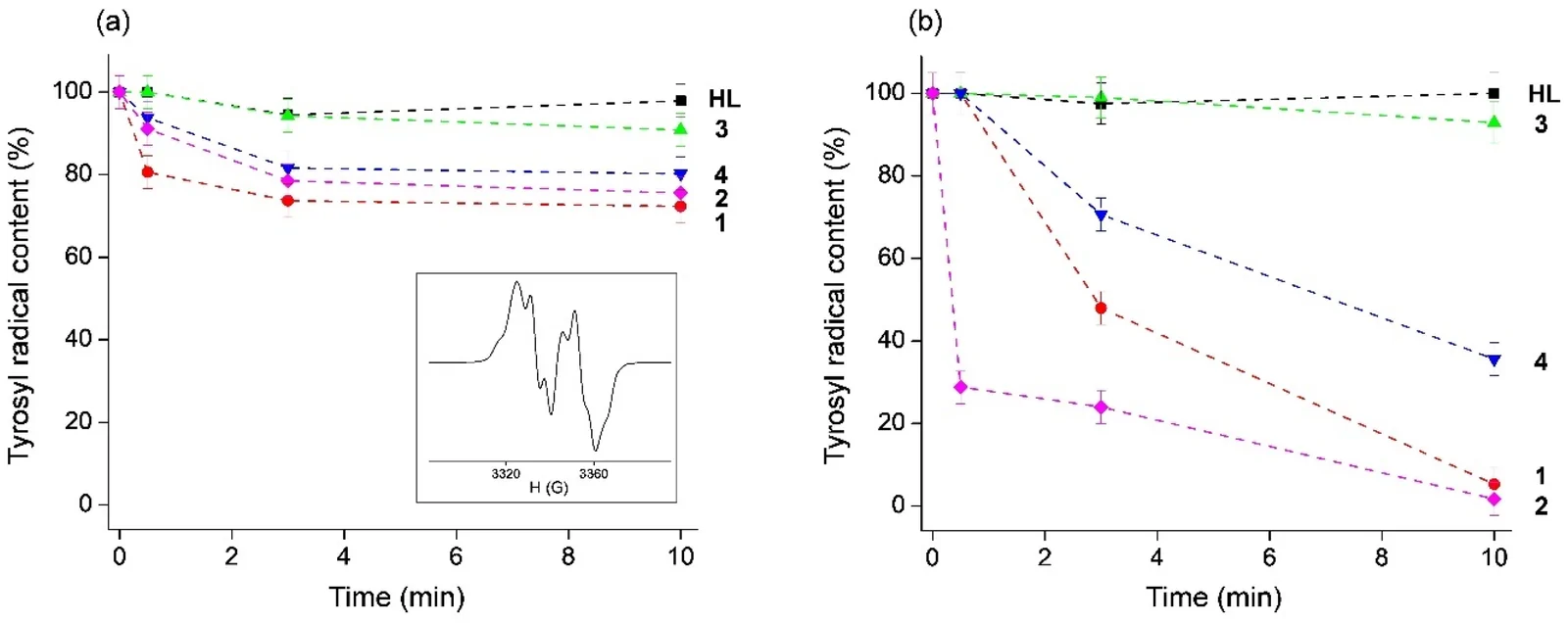
Time-dependent Y˙ reduction in mouse R2 RNR protein by HL and complexes 1–4, at a 1 : 1 protein-to-compound ratio in the (a) absence and (b) presence of DTT. The natural Y˙ decay in the absence and presence of DTT (data shown in Fig. S37, SI) was subtracted for each time point. Inset: 30 K EPR spectrum of the tyrosyl radical in mouse R2 RNR protein.

The kinetics of tyrosyl radical Y˙ reduction in mR2 RNR protein by HL and its metal complexes 1–4 at a 1 : 1 protein-to-compound ratio were investigated by EPR spectroscopy at 30 K (Fig. 10). The investigated compounds do not exhibit marked Y˙ reduction ability in the absence of an external reductant. However, in the presence of 2 mM DTT, both Cu(ii) complexes, 1 and 2, are able to reduce ∼100% Y˙ after 10 min at 298 K. The bis-ligand Fe(iii) complex 4 also displays considerable mR2 inhibition activity, reducing ∼60% Y˙ during this time frame. These results suggest that under reducing conditions, complexes 1, 2, and 4 directly reduce Y˙ in mR2. Of note is that the measured reduction potentials of 1, 2 and 4 are less than 0.2 V *vs.* NHE (−0.05 V, −0.04 V and +0.09 V *vs.* NHE, respectively, Fig. 4a, b and d), indicating that all three reduced complexes upon reoxidation under these conditions should be potent Y˙ reductants.^72^ The Fe(iii) complexes with thiosemicarbazones were reported to be unable to reduce Y˙ if their reduction potential *E*_1/2_ (Fe^III^/Fe^II^) > 0.5 V *vs.* NHE. The observed lower mR2 inhibition by 4, compared to 1 and 2, is likely due to limited accessibility of the large size/steric volume Fe(iii) bis-ligand complex to Y˙. The Schiff base HL was shown not to affect Y˙ in the presence of DTT (Fig. 10b, black symbols), confirming that the proligand is not redox-active and therefore unable to directly reduce Y˙, and moreover, that it does not sequester Fe(ii) from the DTT-reduced mR2 dinuclear iron cluster, which would lead to stoichiometric Y˙ loss.^73^ The lack of inhibitory activity for complex 3 is consistent with its irreversible reduction behavior.

Thus, the observed Y˙ reduction in mR2 RNR protein by HL and 1–4 at a 1 : 1 protein-to-compound ratio in the presence of DTT correlates well with their electrochemical behavior discussed previously, and, in particular with the reduction potentials for Cu(ii)/Cu(i) and Fe(iii)/Fe(ii) in 1, 2 and 4, respectively, irreversible reduction of 3 and redox silence of HL.

### Interference with tubulin polymerization

Inhibition of polymerization of purified tubulin by direct interaction with the Schiff base HL and complexes 1–4 was next studied. As a reference for comparison, combretastatin A-4 (CA-4) was used. As shown in Table 3, the Schiff base was the least active. Among the metal complexes of 1 : 1 metal-to-ligand stoichiometry, the highest inhibitory activity was found for the distorted square-planar Cu(ii) complexes 1 and 2, while the Pd(ii) complex 3 showed remarkable inhibitory activity as well. The bis-ligand Fe(iii) complex 4 showed the highest inhibitory activity in this series of compounds, indicating the importance of metal complex stoichiometry and metal identity for the inhibition of tubulin polymerization in accordance with recently published results with other thiosemicarbazones.^33^

**Table 3:** Inhibition of tubulin polymerization and colchicine binding by HL and 1–4 a

Compound	Inhibition of tubulin^c^	Inhibition of colchicine binding^d^	
	IC_50_ ± SD (µM)	% inhibition ± SD	
		5 µM inhibitor	25 µM inhibitor
CA-4 b	1.0 ± 0.03	96 ± 2	
HL	>20		
1	5.2 ± 0.1		39 ± 0.7
2	6.3 ± 0.4		33 ± 0.5
3	15 ± 2		
4	4.5 ± 0.6		50 ± 3

a Each experiment was performed 3 times, and SDs are presented.

b Combretastatin A-4.

c Inhibition of tubulin polymerization: tubulin was at 10 µM.

d Inhibition of [^3^H]colchicine binding: tubulin, [^3^H]colchicine, and inhibitor were at 0.5, 5.0 and 5.0 (25 µM).

Comparison of the IC_50_ values for cytotoxicity of 1, 2 and 4 in cancer cells (see below) and inhibition of the polymerization of purified tubulin (Table 3) shows that these are mainly in the low micromolar range. The IC_50_ value for direct inhibition of tubulin by 4 is 4.5 µM. The disjunction between antitubulin activity and cytotoxicity of 4 may be caused by different reasons.^74,75^ First, the drug concentrations quoted are almost always the concentrations in the culture medium. The aqueous solubility of Fe(iii) complex 4 is by factors 125 and 100 lower than that of 1 and 2, respectively, while for inhibition assays, solutions in DMSO were used. Neither the volume of the cells nor the proportion of drug that enters the cells are usually known. Second, the drug can be altered in cells to become more active or less active. As also observed recently for other thiosemicarbazones and their Cu(ii) complexes,^32^ complex formation of HL with Fe(iii) and Cu(ii) resulted in significant enhancement of the ability of the proligand to inhibit tubulin assembly. The active metal complexes 1, 2 and 4 were further investigated for their abilities to inhibit the binding of [^3^H]colchicine to tubulin at a concentration of 25 µM, with tubulin and colchicine at 0.5 and 5 µM concentrations, respectively (Table 3).^74,75^ The data obtained indicate that Fe(iii) complex 4 is only slightly superior to complexes 1 and 2 in its ability to inhibit the binding of [^3^H]colchicine to tubulin at 25 µM, but is still 2-fold less potent than CA-4 at a 5 µM concentration (Table 3).

### Cell viability assessment

The cytotoxic and antiproliferative potential of HL and metal complexes 1–4 was evaluated in MCF-7 breast cancer cells using two complementary approaches: (i) fluorescence-based live/dead imaging and (ii) the CellTiter-Glo luminescent cell viability assay. The two methods, measuring related but distinct cellular parameters − a viable cell number *via* image-based counting and metabolic activity *via* ATP quantification,^76^ respectively, provided largely convergent results. Nocodazole was included throughout as a reference mitotic inhibitor^77^ to validate assay performance.

### Qualitative imaging observations

**Fig. 11:**
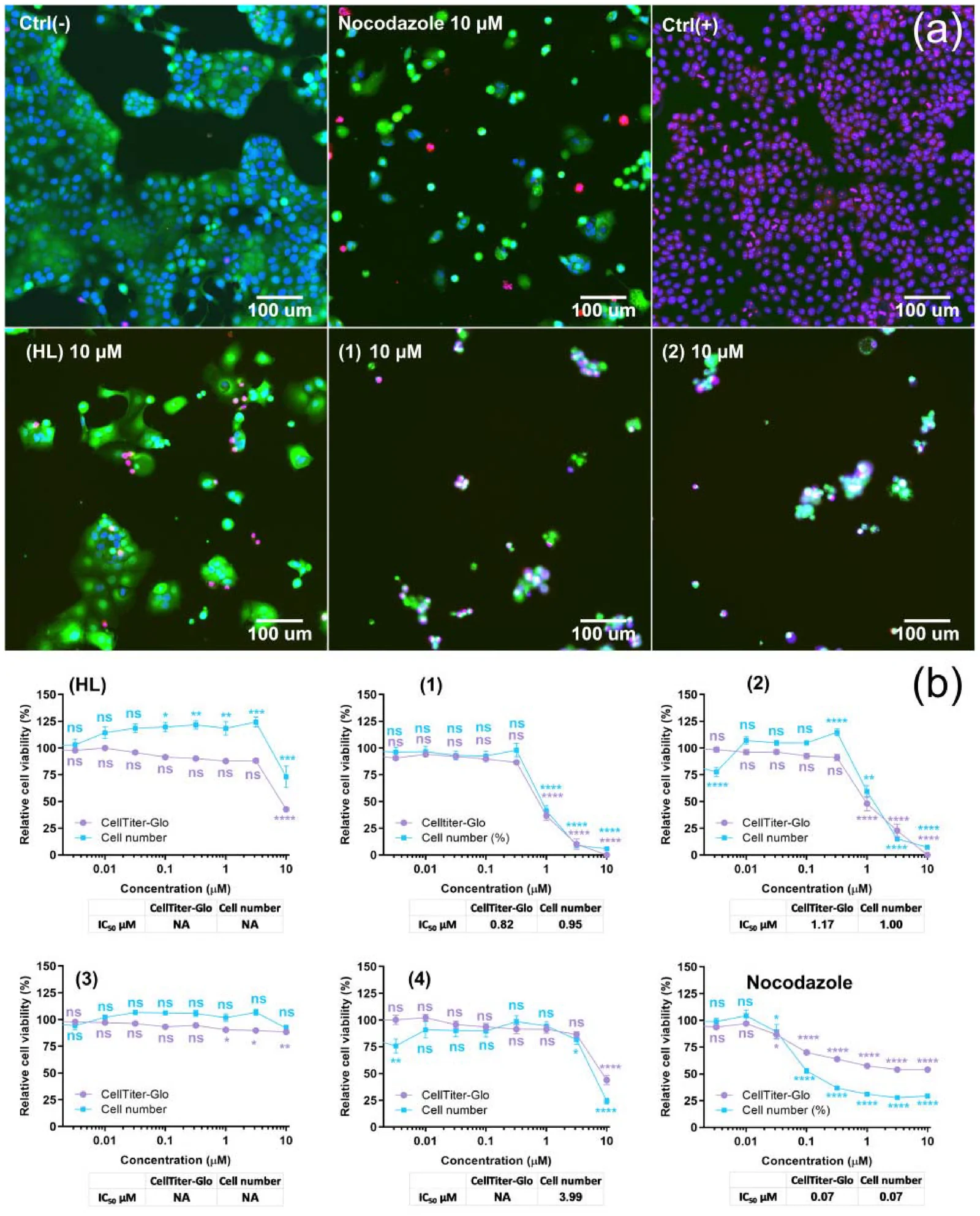
Cytotoxic and antiproliferative effects of HL and metal complexes 1–4 on MCF-7 breast cancer cells after 48 h of treatment: (a) representative fluorescence microscopy images of MCF-7 cells stained with Hoechst 33342 (nuclei, blue), Calcein-AM (viable cells, green), and propidium iodide (PI; dead cells, red/magenta) following 48 h of treatment. Images show untreated cells [Ctrl(−)], nocodazole (10 μM), Triton X-100-treated cells as the dead cell positive control [Ctrl(+)], and HL, 1 and 2 at 10 μM. Scale bars: 100 μm; (b) dose–response curves for HL, 1–4, and nocodazole across a concentration range of 3.18 nM–10 μM. Cell viability was assessed in parallel by two complementary methods: luminescence-based ATP quantification (CellTiter-Glo, purple trace) and image-based viable cell counting (cell number, cyan trace), with both methods normalized to untreated control wells (100%). IC_50_ values derived from each assay are reported below the respective curves (NA: IC_50_ not determinable within the tested concentration range). Data are presented as mean ± SEM from three independent experiments (*N* = 3), with three replicate measurements per experiment for CellTiter-Glo and four replicate measurements per experiment for image-based cell counting. Statistical significance was determined by one-way ANOVA followed by Dunnett's multiple-comparisons test, with each concentration compared with the untreated control (0 μM). Significance is indicated as *ns* (not significant), *p* < 0.05 (*), *p* < 0.01 (**), *p* < 0.001 (***), and *p* < 0.0001 (****).

Representative fluorescence images (Fig. 11a; for the full concentration–response series across all compounds and the nocodazole reference, see Fig. S38, S40, S42, S44, S46, and S48 in the SI) illustrate the differential impact of the tested compounds at the highest concentration (10 μM).

Among the metal complexes, the Cu(ii) complexes 1 and 2 induced the most pronounced reduction in cell number at 10 μM, with the remaining cells frequently appearing rounded and exhibiting co-positivity for Calcein-AM (Calcein Acetoxymethyl ester) and PI (propidium iodide), indicative of late-stage cell death with compromised membrane integrity. This qualitative picture is consistent with potent cytotoxic activity accompanied by marked suppression of cell proliferation. Bright-field morphological images acquired in parallel over the full concentration range (Fig. S39, S41, S43, S45, S47, and S49 in the SI, for HL, 1–4, and nocodazole, respectively) further corroborated these observations. At concentrations of 1 μM and above, cultures treated with Cu(ii) species 1 and 2 displayed increasing fractions of rounded and detached cells, whereas those treated with Pd(ii) complex 3 and iron(iii) complex 4 retained morphologies indistinguishable from the untreated control across the entire concentration range tested.

The Schiff base HL, from which all metal complexes were obtained, at 10 μM (Fig. S38, SI) produced a visibly reduced cell density compared to the untreated control, with surviving cells retaining an elongated, spread morphology and relatively preserved Calcein-AM signal. This observation suggests that HL alone exerts, at most, a weak cytostatic tendency effective only at the upper limit of the tested concentration range, and that its biological activity is substantially lower than that of the Cu(ii) complexes 1 and 2 derived from it.

Quantitative dose–response analysis (Fig. 11b) confirmed and extended these qualitative observations, revealing a clear structure–activity relationship across the compound series. Nocodazole was included throughout as a reference mitotic inhibitor to validate assay performance (Fig. S48 and S49). The free ligand HL did not produce a determinable IC_50_ by CellTiter-Glo across the full concentration range, while a very modest reduction in cell number was observed only at the highest concentration tested (10 μM), reinforcing the conclusion that coordination to a metal center is necessary for potent biological activity.

The Pd(ii) complex 3 showed no significant effect on either readout across the entire concentration range, with IC_50_ values not determinable for both assays, indicating that complex formation with Pd(ii) does not confer cytotoxic activity to HL under the tested conditions. The Fe(iii) complex 4 produced a marked reduction in cell number (IC_50_ ∼4 μM by cell count) and CellTiter-Glo signal only at the highest concentration tested. Further increase of concentration was not possible because of poor solubility of this compound.

In strong contrast to the rest of the series of compounds studied, the Cu(ii) complexes 1 and 2 displayed potent concentration-dependent activity with better defined sigmoidal dose–response curves and sub-micromolar to low-micromolar IC_50_ values. Compound 1 yielded IC_50_ values of 0.82 μM (CellTiter-Glo) and approximately 0.95 μM (cell number), with excellent concordance between the two assays, indicating that the reduction in ATP signal faithfully reflects genuine cell loss rather than a selective impairment of cellular metabolism in otherwise surviving cells. Compound 2 demonstrated a weaker but still comparable potency, with IC_50_ values of 1.17 μM and approximately 1 μM by CellTiter-Glo and cell counting, respectively. The close agreement between the two assay modalities strongly indicates that the biological effects of both compounds are predominantly cytotoxic rather than purely cytostatic, with cell death representing the major outcome at effective concentrations.

Taken together, the data reveal a pronounced and selective enhancement of cytotoxic activity upon coordination of HL to Cu(ii), an effect not observed by complex formation with Pd(ii) or Fe(iii) under the same conditions. This metal-dependent divergence in biological activity is a noteworthy finding and suggests that the Cu(ii) center plays an integral role in the mechanism of cytotoxicity. The near-identical potency of 1 and 2, both Cu(ii) complexes differing in their ancillary co-ligands (DCA and chlorido, respectively), further implies that the Cu(ii) coordination sphere is the primary determinant of activity, while structural modifications at the periphery of the complex have limited impact on cytotoxic potency in this cell line. The inability of Pd(ii) to undergo redox transformations is one potential reason of lower antiproliferative activity of Pd(ii) complex 3 compared to Cu(ii) complexes 1 and 2. Cu(ii) forms more labile complexes that readily exchange ligands and interact with biological targets, while Pd(ii) complexes are too inert to be competitive in this respect with Cu(ii). In addition, Cu(ii), as a borderline Lewis acid, prefers binding to mixed N and O donors, while Pd(ii) as a soft acid prefers binding to soft bases, *e.g.*, sulfur or phosphorus.

These findings highlight Cu(ii) compounds 1 and 2 as the most promising candidates within this series for further mechanistic investigation.

### Cell cycle analysis

**Fig. 12:**
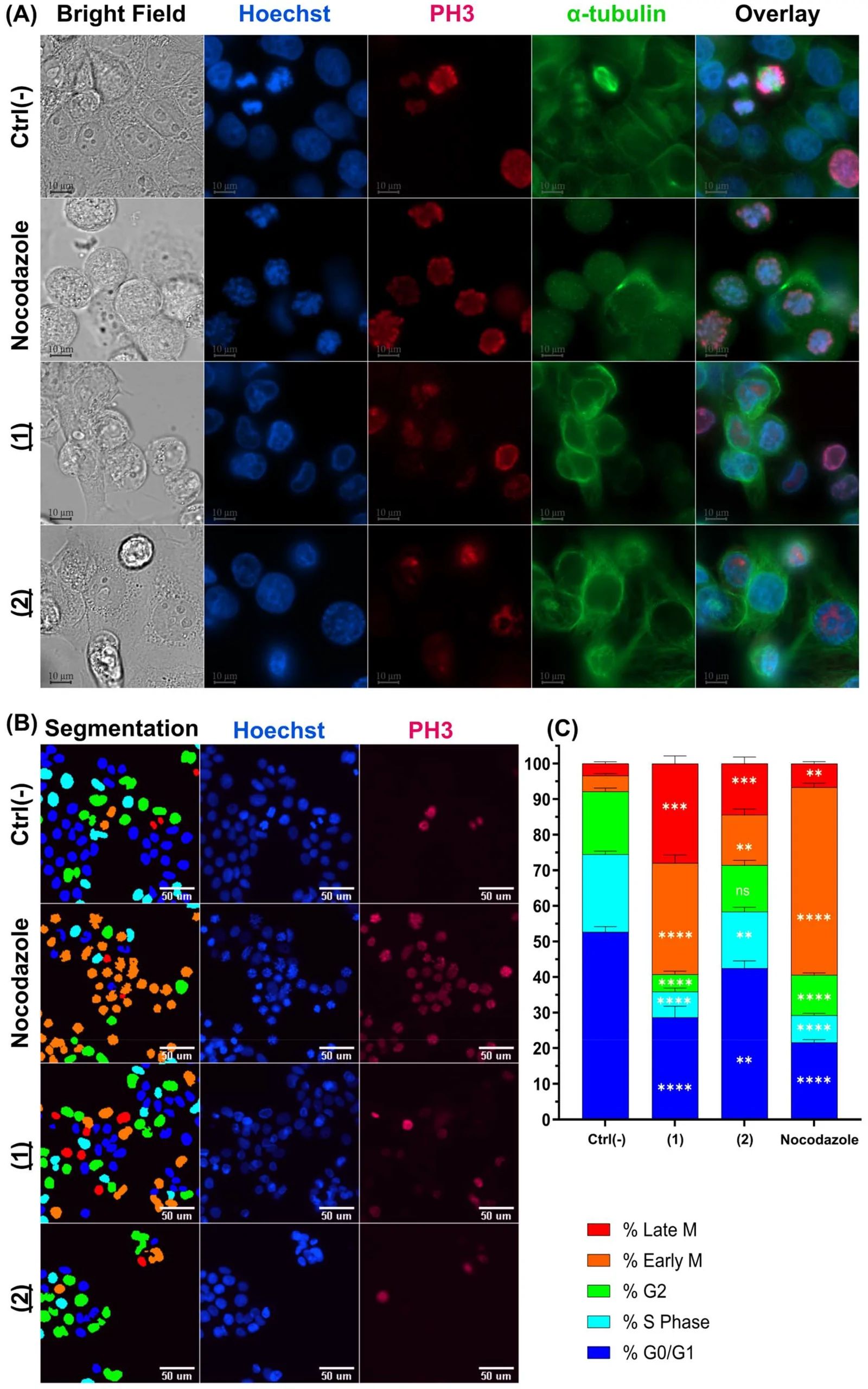
Cell cycle analysis of MCF-7 cells following 21 h of treatment with 1 and 2 at 3.16 μM: (A) representative high-magnification fluorescence images acquired using a Zeiss Axio Observer 7 microscope, showing untreated cells [Ctrl(−)], nocodazole, and compounds 1 and 2. For each condition, five channels are displayed: bright field, Hoechst 33342 (DNA/nuclei, blue), phospho-Histone H3 Ser10 (PH3; mitotic marker, red), α-tubulin (microtubule network, green), and a merged overlay of all fluorescence channels. Scale bars: 10 μm; (B) representative images from quantitative high-content analysis performed using an ImageXpress® Confocal HT.ai system (Molecular Devices), showing automated nuclear segmentation with individual cells pseudo-colored according to their assigned cell cycle phase (G0/G1, blue; S phase, cyan; G2, green; Early M, orange; and Late M, red), alongside the corresponding Hoechst 33342 and PH3 channel images used for phase classification. Scale bars: 50 μm; (C) quantitative cell cycle distribution of MCF-7 cells, expressed as the percentage of cells in each phase: G0/G1 (blue), S phase (cyan), G2 (green), Early M (orange), and Late M (red). Phase assignment was performed using the MetaXpress Cell Cycle module, based on Hoechst 33342 DNA content and PH3 immunoreactivity as a mitosis-specific marker. A number of 24 microscopic fields per condition were analyzed. Data are presented as mean ± SEM from three independent experiments (*N* = 3), with eight replicate measurements per condition in each experiment. Statistical significance was determined by two-way ANOVA followed by Dunnett's multiple-comparisons test, with each cell-cycle phase for each treatment compared with the corresponding phase in the untreated control. Significance is indicated as *ns* (not significant), *p* < 0.05 (*), *p* < 0.01 (**), *p* < 0.001 (***), and *p* < 0.0001 (****).

The immunofluorescence-based cell cycle analysis revealed distinct effects of 1 and 2 on cell cycle distribution in MCF-7 cells, with nocodazole included as a positive control for mitotic arrest (Fig. 12). In untreated cells, the expected distribution of an asynchronously cycling population was observed, with the majority of cells residing in G0/G1, moderate S-phase and G2 fractions, and smaller proportions in mitosis (Early and Late M). The α-tubulin staining in control cells displayed well-organized interphase microtubule networks, and phospho-Histone H3 (PH3) positivity was confined to the rare mitotic cells expected in a normally proliferating culture.

Nocodazole treatment produced a pronounced redistribution of the cell population toward mitotic phases, consistent with its established mechanism of microtubule depolymerization and spindle assembly checkpoint activation.^77^ This was reflected both qualitatively, with the bright field and overlay images showing abundant rounded, mitotic cells with an intense PH3 signal and disrupted α-tubulin architecture, and quantitatively, as the stacked bar chart demonstrated a marked increase in the combined mitosis fractions (especially early mitosis) at the expense of the G0/G1 population.

Complex 1 induced a cell cycle profile that shared notable similarities with the nocodazole-treated group, with a visible increase in both early and late mitotic fractions relative to the untreated control, alongside a reduction in the G0/G1 cell population. Qualitative microscopic examination supported this observation, as cells treated with 1 exhibited morphological features consistent with mitotic arrest, including nuclear condensation detectable *via* Hoechst staining and elevated PH3 immunoreactivity. The retained but altered α-tubulin signal in these cells may suggest interference with microtubule dynamics or spindle organization, though the extent of disruption appeared less severe than that induced by nocodazole. Complex 2 also produced a discernible shift in cell cycle distribution. Specifically, this shift is characterized by a reduction in the G0/G1 fraction with a corresponding increase in the mitotic subpopulations (Early M and Late M), while the S-phase and G2 fractions remained largely comparable to those of the untreated control pointing to interference at the level of mitotic entry or progression rather than at earlier cell cycle checkpoints. The α-tubulin network in 2-treated cells appeared more preserved relative to compound 1, further supporting a mechanistic distinction between the two complexes. Collective evidence suggests that compounds 1 and 2 exert antiproliferative activity in MCF-7 cells through cell cycle perturbation, albeit potentially *via* distinct mechanisms. M phase arrest is a characteristic phenotype of MCF-7 cells incubated with complexes 1 and 2.

Given that complexes 1, 2, and 4 potently reduce Y˙ in mR2 RNR protein in cell-free settings (Fig. 10), a perturbation of S-phase progression might be anticipated in cells, since RNR inhibition restricts dNTP supply and thereby activates the intra-S-phase checkpoint through ATR–CHK1 signaling, producing replication fork stalling and, at sufficient inhibitor concentrations, a global S-phase arrest.^78,79^ However, quantitative cell cycle profiling of MCF-7 cells treated with 1 and 2 at 3.16 μM for 21 h did not reveal an increase in the S-phase population relative to untreated controls (Fig. 12C). Instead, the dominant effect was a marked redistribution toward early and late mitotic phases with a reduction of the G0/G1 fraction, a phenotype consistent with spindle assembly checkpoint (SAC) activation downstream of tubulin-targeted activity. Because the analysis was performed at a single treatment time point, the absence of a detectable S-phase signal cannot distinguish between a genuine lack of cellular RNR-mediated cell-cycle perturbation and a transient or earlier S-phase effect that had resolved by 21 h. The present data therefore support the conclusion that mitotic accumulation is the dominant phenotype at 21 h, but they do not establish the temporal sequence or relative contribution of RNR and tubulin inhibition in cells.

An interesting finding of the present study is the apparent disconnect between the efficient quenching of the mR2 RNR tyrosyl radical by complexes 1, 2, and 4 in cell-free EPR assays (Fig. 10) and the absence of a detectable S-phase arrest phenotype in MCF-7 cells (Fig. 12). This discordance warrants mechanistic consideration, as it touches on the fundamental question of how dual-target inhibitory activity observed in biochemical settings translates − or fails to translate − into the expected cellular phenotype. The critical variable governing the observed cellular phenotype is the operational difference between a transient kinetic delay and a terminal trap.

One possible interpretation is that R2 RNR inhibition produces a partial depletion of intracellular dNTP pools and therefore slows progression through the S phase rather than producing a persistent arrest. Under this model, cells experiencing RNR-mediated replication stress could eventually complete replication and progress into G2 and M, where the tubulin-targeted activity could produce the observed mitotic accumulation. However, this kinetic model is not directly demonstrated by the present data because no time-dependent cell-cycle measurements were performed. It should therefore be regarded as a mechanistic hypothesis that requires temporal validation.

A complementary interpretation is that the downstream tubulin-targeted activity functions as a terminal mitotic block. Upon entering mitosis, cells may encounter disrupted microtubule dynamics that prevents proper spindle assembly and activates the SAC, consistent with the mitotic phenotype observed here. Such a mechanism could, in principle, mask an earlier RNR-dependent S-phase perturbation in a single-time-point population analysis. Nevertheless, without measurements at multiple time points, the present study cannot demonstrate that cells first experience an RNR-mediated S-phase delay and subsequently accumulate in mitosis. The observed mitotic accumulation is supported experimentally, whereas the proposed temporal sequence remains to be tested.

This asymmetry is further accentuated by the specific genetic background of the MCF-7 cell line, which is natively caspase-3 deficient due to a well-documented deletion in exon 3 of the *CASP3* gene.^80,81^ In the absence of functional caspase-3 executioner activity, MCF-7 cells are highly resistant to rapid apoptotic clearance when experiencing replication stress or DNA damage. Instead of being eliminated from the population during DNA synthesis, these cells sustain prolonged checkpoint survival, enabling them to slowly finalize genome duplication and migrate forward into the dominant mitotic block.

## Conclusions

In this study, by *in silico* searching for metal thiosemicarbazonates able to inhibit both R2 RNR and tubulin polymerization, we found that a Schiff base HL, derived from quinoline-8-carboxaldehyde and *N*4-(3,4,5-trimethoxyphenyl)thiosemicarbazide, is a good candidate for the synthesis of metal complexes. We synthesized and comprehensively characterized two distorted square-planar Cu(ii) complexes, **[Cu**^**II**^**(L)(Cl**_**2**_**CHCOO)]** (1) and **[Cu**^**II**^**(L)Cl]** (2), a square-planar Pd(ii) complex, **[Pd**^**II**^**(L)Cl]** (3), and an octahedral Fe(iii) complex, **[Fe**^**III**^**(L)**_**2**_]**Cl** (4). Both Cu(ii) complexes were found to be good inhibitors of mR2 RNR protein and tubulin polymerization. Furthermore, the Cu(ii) complexes 1 and 2 showed prominent antiproliferative activity against MCF-7 human breast adenocarcinoma cells, while HL and complex 3 revealed considerably reduced cytotoxicity within the tested concentration range. As R2 protein and tubulin polymerization inhibitors are expected to affect different phases of the cell cycle, S and G2/M, respectively, it was of particular interest to investigate the effects in cancer cells. Cell cycle analysis showed that 1 and 2 induced redistribution of MCF-7 cells toward the mitotic phase at the 21 h time point, whereas no statistically significant change in the S-phase population was detected. These data establish mitotic accumulation as the dominant phenotype under the conditions tested. However, because cell-cycle analysis was performed at a single time point, the present results cannot determine whether an earlier or transient S-phase perturbation occurs before mitotic accumulation, nor can they establish the temporal or quantitative contribution of cellular R2 RNR inhibition to the antiproliferative phenotype. Given that Cu(ii) and Pd(ii) complexes 2 and 3, respectively, are both square-planar complexes, it appears that the shape of the complex is less important than the electronic structure, which is different for Cu(ii) (d^9^, *S* = ½) and Pd(ii) (d^8^, *S* = 0), for binding to the colchicine site of tubulin. This distinct electronic structure of the central metal ion in 2 and 3 can result in differences in the charge distributions or distribution of hydrophobic and hydrophilic regions on the surface of the Cu(ii) and Pd(ii) complexes, and influence their interaction with the colchicine binding site, as reported recently for di-Co(ii) (d^7^) and di-Fe(ii) (d^6^) helices with distinct biological activities.^40^

## Experimental

All experimental information (materials and methods) and experimental procedures (synthesis of the ligand and metal complexes, and characterization data) are in the SI.

## Author contributions

O. P.: methodology and investigation; B. I. C.: investigation, visualization, and formal analysis; A. C. S.: methodology, software, and investigation; M. H.: methodology, investigation, and validation; M. Ha.: methodology and investigation; M. B. P.: investigation, validation, and formal analysis; S. S.: investigation and writing − original draft; G. T. G.: methodology, investigation, validation, and formal analysis; A. P. B.: investigation, validation, and writing − original draft; J. R.: investigation, validation, software, formal analysis, visualization, and writing − original draft; M. J.: methodology, investigation, and validation; M. Z.: data curation, investigation, validation, and formal analysis; P. R.: investigation, validation, and writing − original draft; É. A. E.: investigation, validation, supervision, and writing − original draft; D. P.: conceptualization, investigation, validation, formal analysis, writing − original draft, and writing − review & editing; M. D.: validation, formal analysis, and project administration; M. C.: investigation, writing − review & editing, and supervision; E. H.: investigation, validation, formal analysis, and data curation; V. B. A.: funding acquisition, supervision, writing − original draft, and writing − review & editing.

## Conflicts of interest

There are no conflicts to declare.

## Supplementary Material

QI-013-D6QI01591C-s001

QI-013-D6QI01591C-s002

QI-013-D6QI01591C-s003

## Data Availability

Supplementary information (SI): all experimental details and characterization data. See DOI: https://doi.org/10.1039/d6qi01591c. CCDC 2540743–2540746 contain the supplementary crystallographic data for this paper.^82*a*–*d*^
