# Supplementary material for: Metal thiosemicarbazonates as dual mR2 RNR and colchicine-site tubulin inhibitors: from biochemical inhibition to mitotic arrest in MCF-7 breast cancer cells
Source: Inorg Chem Front. 2026 Sep 3. Online ahead of print. doi: 10.1039/d6qi01591c (PMC13592152; doi:10.1039/d6qi01591c)
Supplement: Supplementary file 1 [file QI-OLF-D6QI01591C-s001.pdf]

Supplementary Information (SI) for Inorganic Chemistry Frontiers.
This journal is © the Partner Organisations 2026

ELECTRONIC SUPPLEMENTARY INFORMATION

for

Metal Thiosemicarbazonates as Dual mR2 RNR and Colchicine-Site Tubulin Inhibitors: From Biochemical Inhibition to Mitotic Arrest in MCF-7 Breast Cancer Cells

Oleg Palamarciuc,[a,b] Bianca-Iulia Ciubotaru,[a,c] Alexandru-Constantin Stoica,[a] Michaela Hejl,[d] Marta Hammerstad,[e] Mihaela Balan-Porcarasu,[a] Sergiu Shova,[a] Gerda T. Gátszegi,[f] Ana Popović Bijelić,[g] Jóhannes Reynisson,[h] Michael A. Jakupec,[d] Michal Zalibera,[i] Peter Rapta,[i] Éva A. Enyedy,[f] Dragos Peptanariu,[j,*] Mihaela Dascalu,[a] Maria Cazacu,[a] Ernest Hamel,[k] Vladimir B. Arion[a,d,*]

[a]*Department of Inorganic Polymers, "Petru Poni" Institute of Macromolecular Chemistry, Romanian Academy, Aleea Gr. Ghica Voda 41 A, Iasi 700487, Romania*

[b]*Physics of Semiconductors and Devices Laboratory, Faculty of Physics and Engineering and Institute of Applied Physics, Moldova State University, MD-2009 Chișinău, Republic of Moldova*

[c]*Department of Biomedical Sciences, Faculty of Medical Bioengineering, Grigore T. Popa University of Medicine and Pharmacy, 11-13 Kogalniceanu Street, 700454 Iasi, Romania*

[d]*Institute of Inorganic Chemistry, Faculty of Chemistry, University of Vienna, Währinger Strasse 42, A-1090 Vienna, Austria*

[e]*Section for Biochemistry and Molecular Biology, Department of Biosciences, University of Oslo, P.O. Box 1066, Blindern, NO-0316 Oslo, Norway*

[f]*Department of Molecular and Analytical Chemistry, University of Szeged, Dóm tér 7-8, H-6720 Szeged, Hungary*

[g]*Faculty of Physical Chemistry, University of Belgrade, 11158 Belgrade, Serbia*

[h]*School of Allied Health Professions and Pharmacy, Keele University, Hornbeam Building, Staffordshire ST5 5BG, United Kingdom*

[i]*Institute of Physical Chemistry and Chemical Physics, Faculty of Chemical and Food Technology, Slovak University of Technology in Bratislava, SK-81237 Bratislava, Slovakia*

[j]*Centre of Advanced Research in Bionanoconjugates and Biopolymers, "Petru Poni" Institute of Macromolecular Chemistry, Romanian Academy, Grigore Ghica Voda Alley 41 A, 700487 Iasi, Romania*

[k]*Molecular Pharmacology Branch, Developmental Therapeutics Program, Division of Cancer Treatment and Diagnosis, National Cancer Institute, Frederick National Laboratory for Cancer Research, National Institutes of Health, Frederick, Maryland 21702, United States*

*corresponding authors: vladimir.arion@univie.ac.at; peptanariu.dragos@icmpp.ro

Table of Contents

R2 RNR protein and its active site S3

The two subunits of tubulin and colchicine binding site S4

Molecular modeling S4

ESI mass spectra and NMR spectra S15

IR spectra S25

X-ray diffraction data S28

Spectroelectrochemistry S31

Solution studies S35

R2 RNR inhibition S37

Biological data S38

Materials S44

Synthesis of the ligand and metal complexes S44

Methods S47

R2 RNR protein and its active site

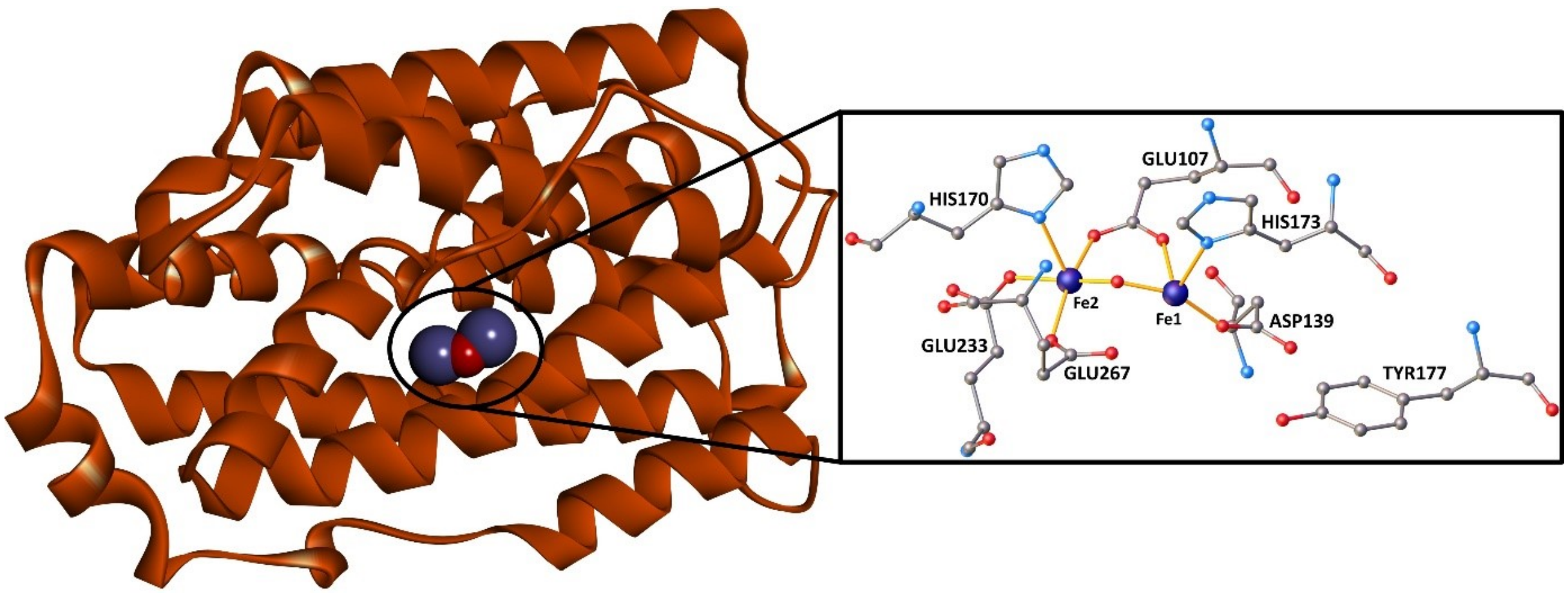

Figure S1. The RNR class Ia mR2 subunit (PDB ID: 1w68) displayed as a monomer (left). The mR2 protein active site with di-iron(III)-μ-oxido cluster and tyrosyl radical (right).

The two subunits of tubulin and colchicine binding site

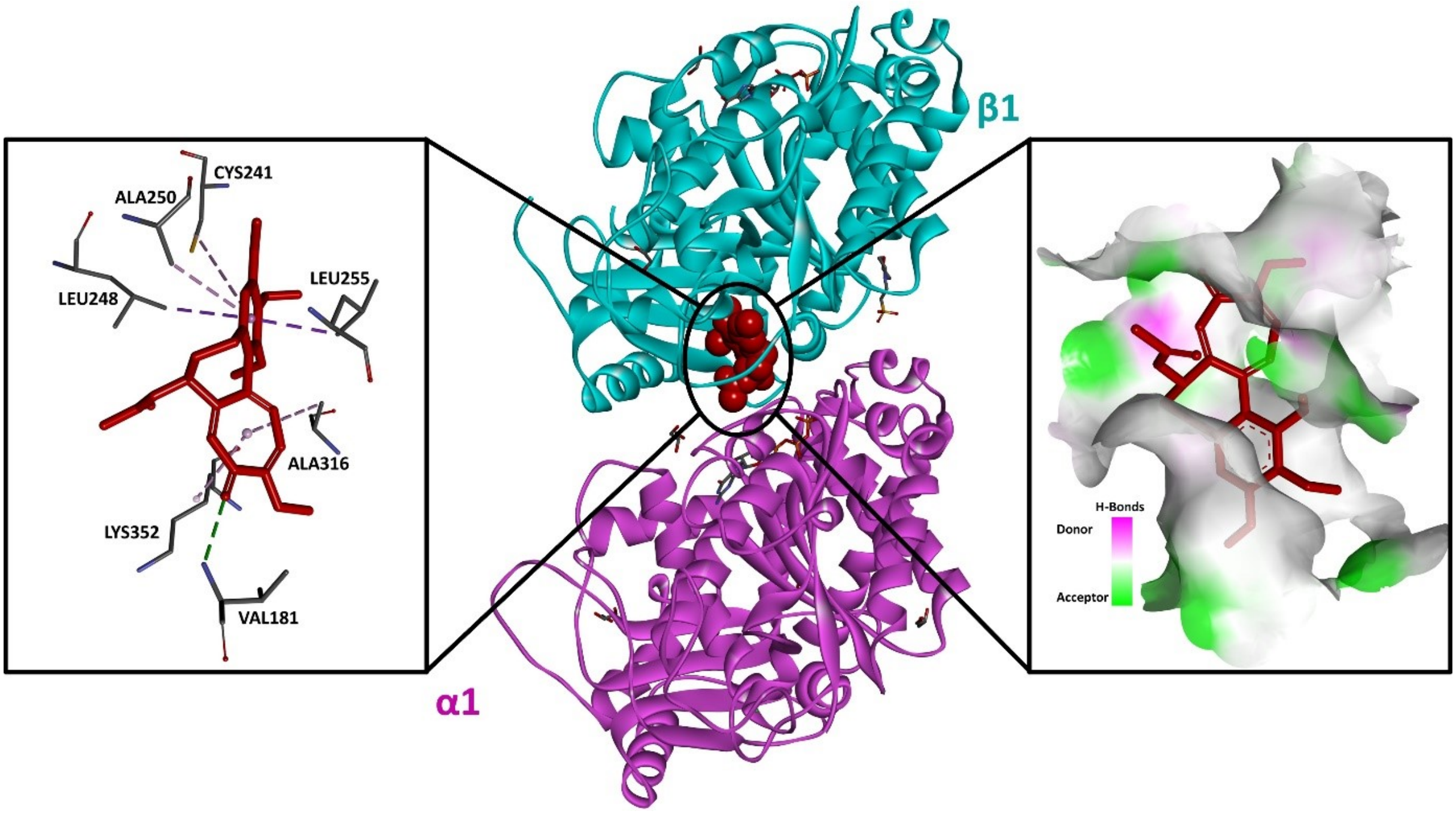

Figure S2. The two subunits of tubulin α1 (magenta) and β1 (cyan) and colchicine binding site of microtubule-targeting agents (MTAs) (PDB ID 4O2B) at the interface of them.

Molecular modeling

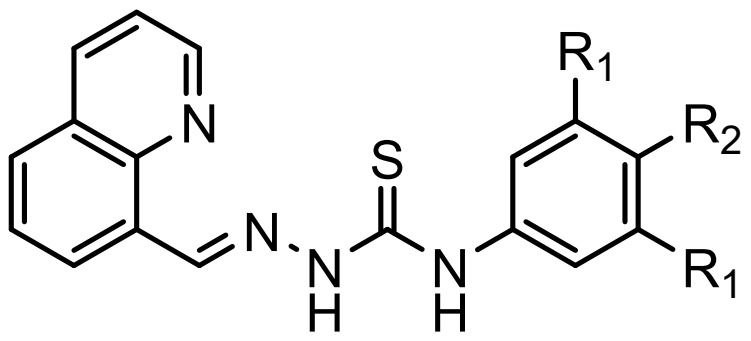

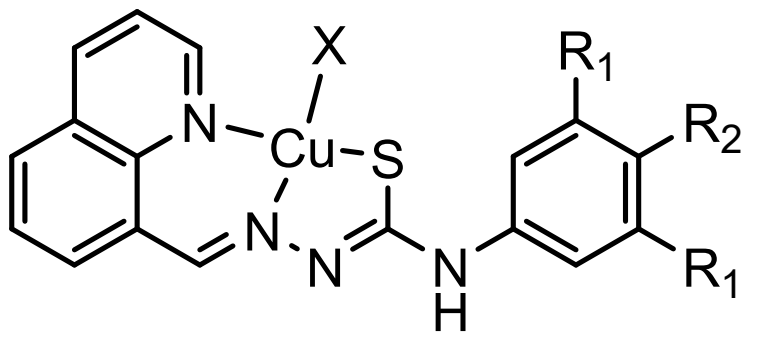

$R_1 = R_2 = H$ ($\mathbf{HL^{H}}$)
$R_1 = R_2 = OH$ ($\mathbf{HL^{OH}}$)
$R_1 = Cl$, $R_2 = H$ ($\mathbf{HL^{Cl,H}}$)
$R_1 = R_2 = OMe$ (**HL**)

$R_1 = R_2 = H$, $X = CHCl_2COO$ **[Cu(L^{H})DCA]**
$R_1 = R_2 = H$, $X = Cl$ **[Cu(L^{H})Cl]**
$R_1 = R_2 = OH$, $X = CHCl_2COO$ **[Cu(L^{OH})(DCA]**
$R_1 = R_2 = OH$, $X = Cl$ **[Cu(L^{OH})Cl]**
$R_1 = Cl$, $R_2 = H$, $X = CHCl_2COO$ **[Cu($L^{Cl,H}$)DCA]**
$R_1 = Cl$, $R_2 = H$, $X = Cl$ **[Cu($L^{Cl,H}$)Cl]**
$R_1 = R_2 = OMe$, $X = CHCl_2COO$ **1**
$R_1 = R_2 = OMe$, $X = Cl$ **2**

Chart S1. Line drawings of the library of 12 compounds used for preliminary molecular docking calculations.

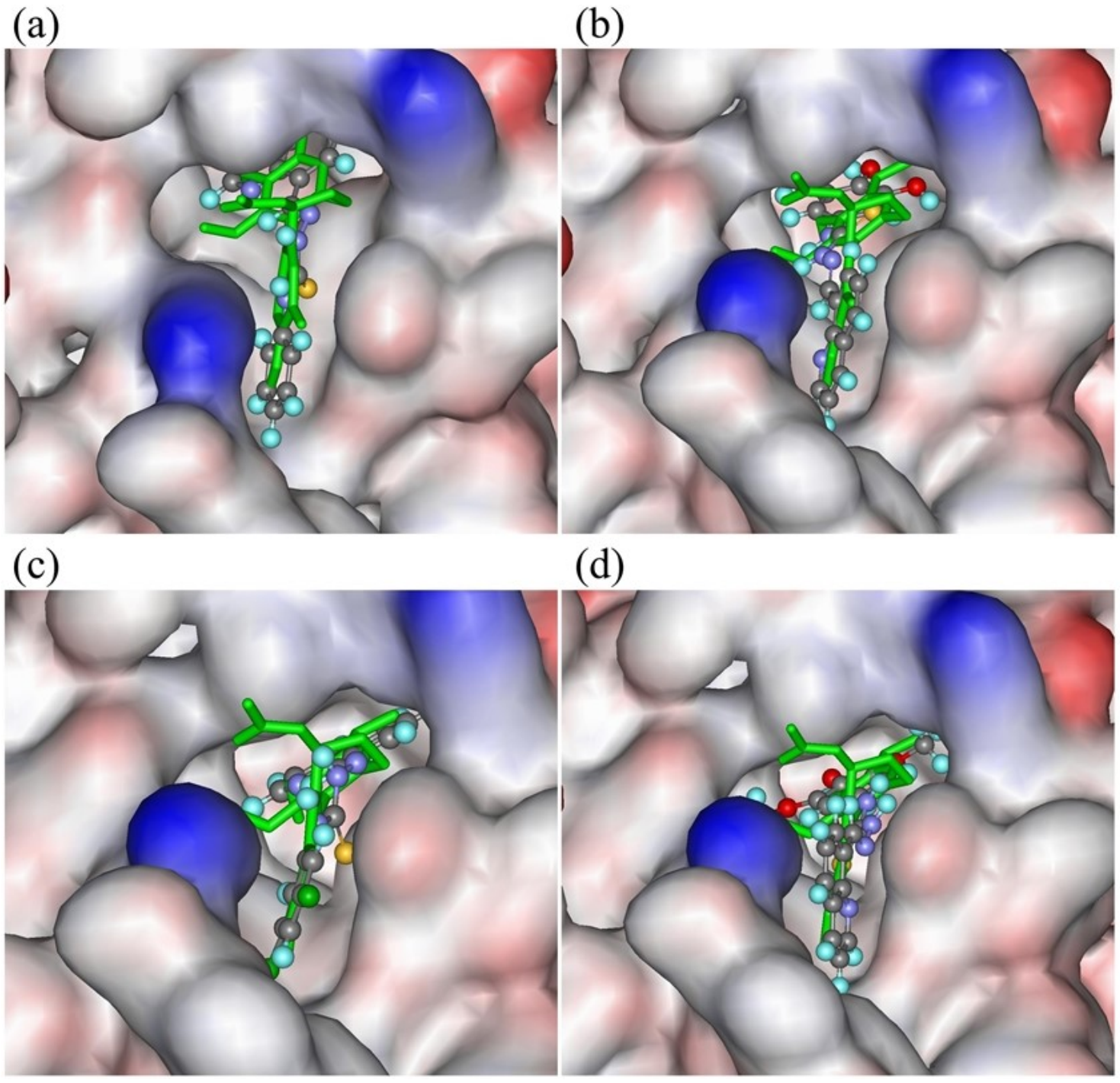

Figure S3. The docked pose of the ligands (a) **HL^{H}**, (b) **HL^{OH}**, (c) **HL^{Cl}** and (d) **HL** (ball-and-stick representation) in colchicine binding site, the co-crystallized ligand LOC (colchicine) is shown in line format (green). The hydrogen atoms were omitted for clarity. The protein surface is rendered; blue color depicts regions with a partial positive charge on the surface, while red color depicts regions with a partial negative charge and grey shows neutral areas;

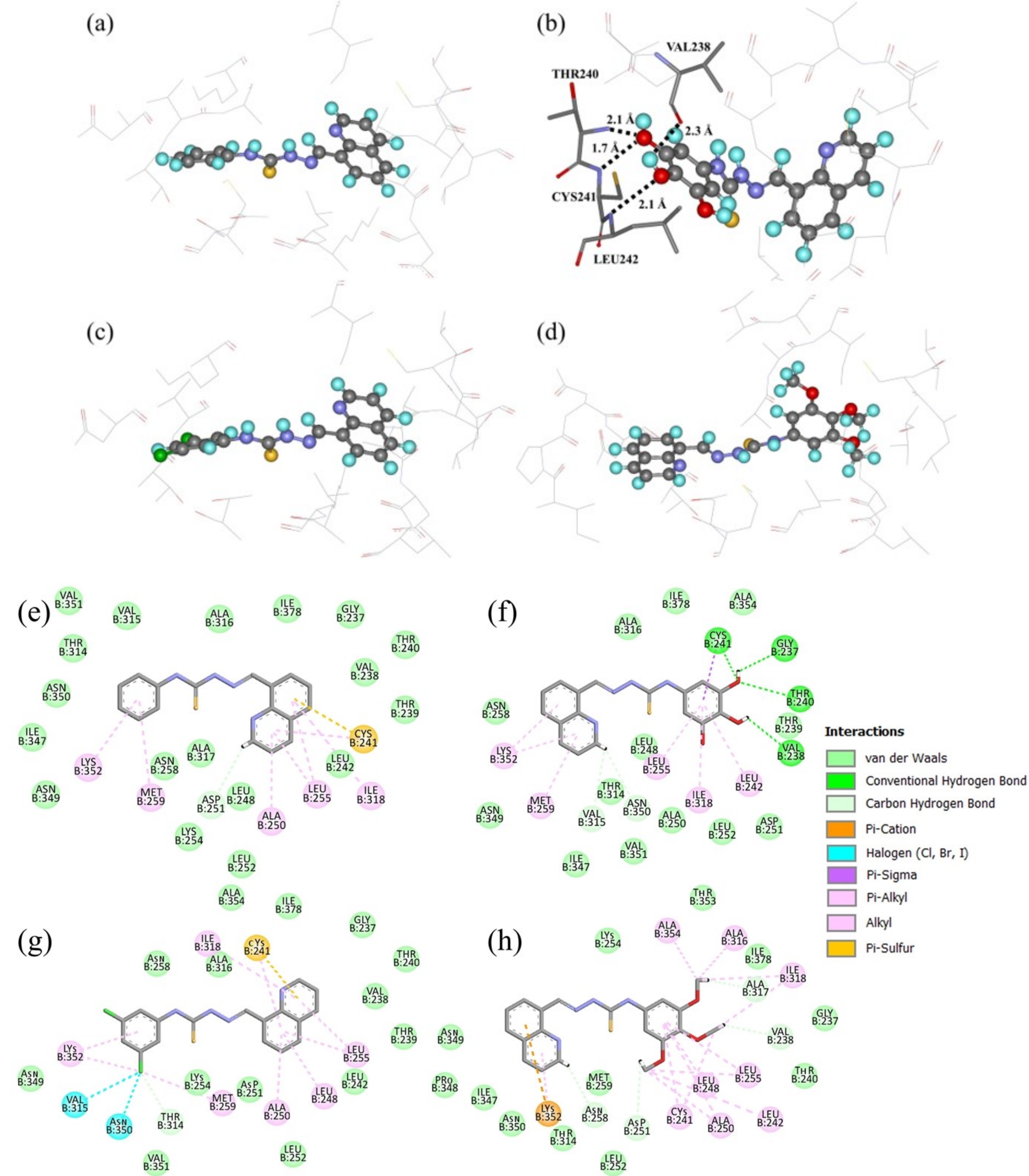

Figure S4. Predicted bindings for (a) **HL^H^**, (b) **HL^OH^**, (c) **HL^Cl^**, and (d) **HL** with amino acids in the protein pocket. Amino acids located within 5 Å distance were selected; amino acids that do not establish interactions are represented in line format, and H-bonding is represented with black dashed line. The 2D representation of all the intermolecular interactions between colchicine binding site and (e) **HL^H^**, (f) **HL^OH^**, (g) **HL^Cl^**, and (h) **HL**. Green dashed lines represent hydrogen bonds. All the other dashed lines represent various types of non-covalent interactions specified in the inset to this part of the figure. Light green colored circles indicate the residues involved in van der Waals interactions.

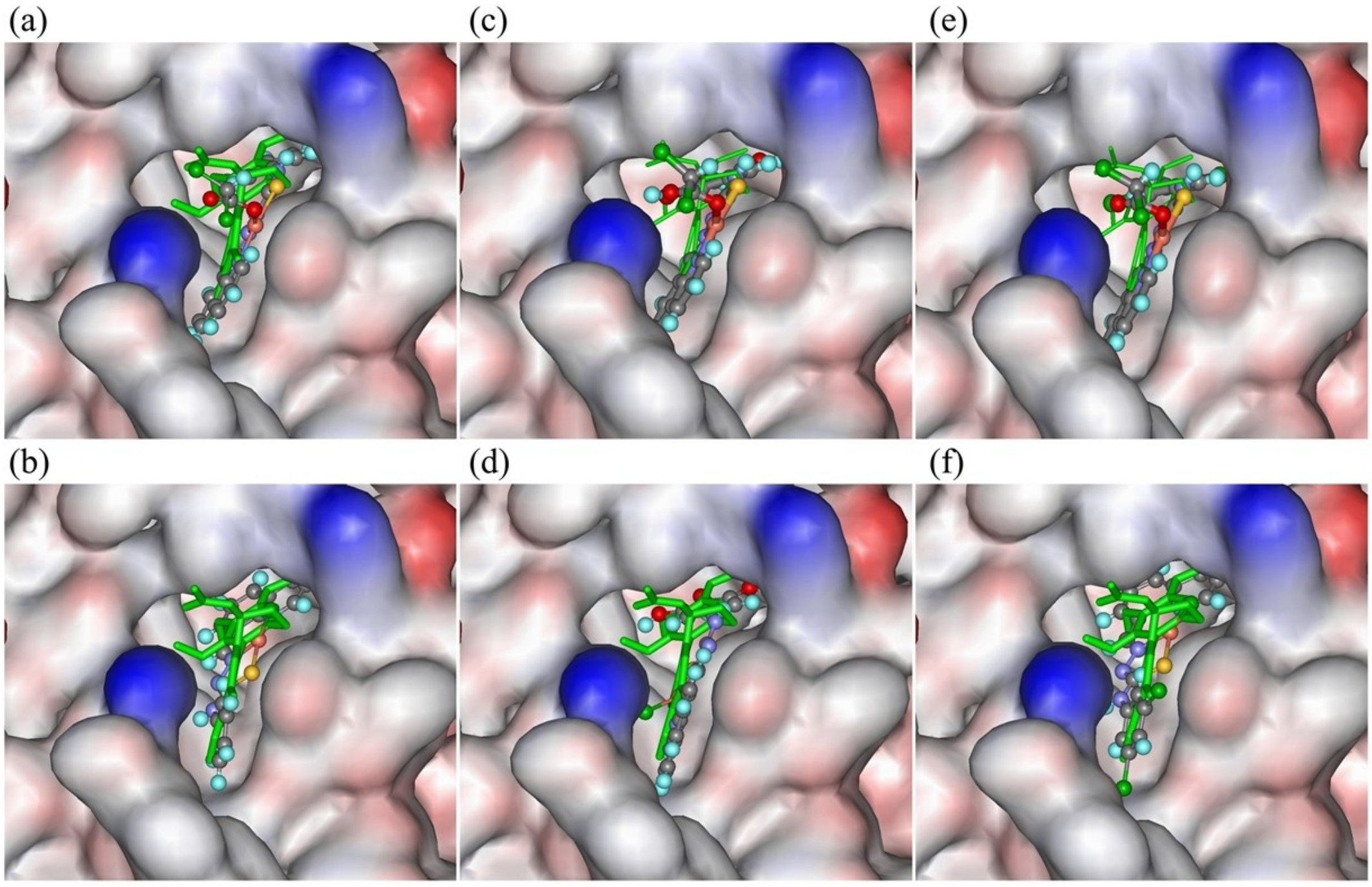

Figure S5. The docked pose of the ligands; (ball-and-stick) in colchicine binding site of tubulin, the co-crystallized ligand LOC (colchicine) is shown in line format (green), its hydrogens are not shown for clarity. The protein surface is rendered; blue depicts regions with a partial positive charge on the surface; red depicts regions with a partial negative charge and gray shows neutral areas; (a) **Cu(L^{H})DCA**, (b) **Cu(L^{H})Cl**, (c) **Cu(L^{OH})DCA**, (d) **Cu(L^{OH})Cl**, (e) **Cu(L^{Cl,H})DCA**, (f) **Cu(L^{Cl,H})Cl**.

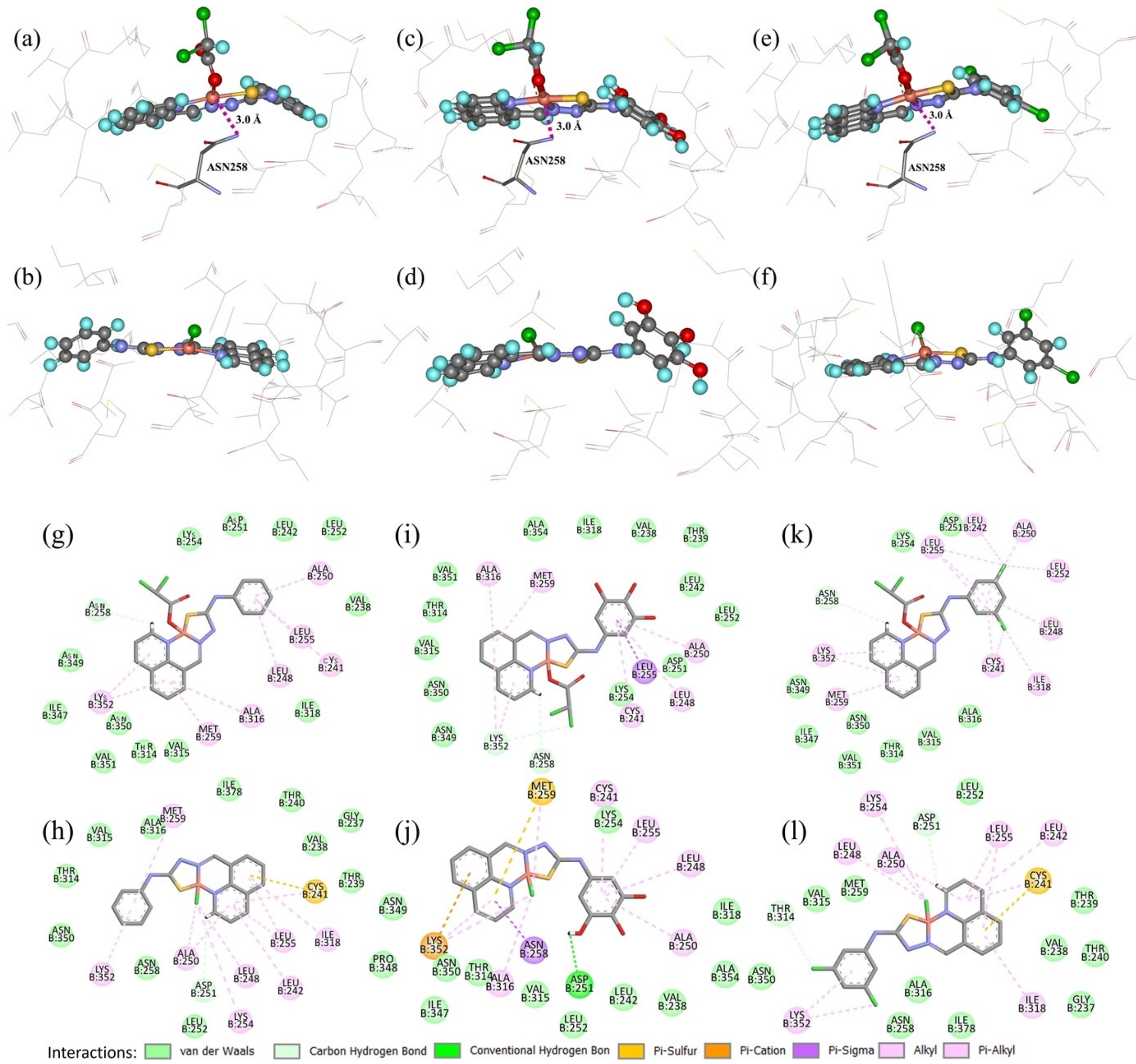

Figure S6. Predicted bindings for (a) **Cu(L^{H})DCA**, (b) **Cu(L^{H})Cl**, (c) **Cu(L^{OH})DCA**, (d) **Cu(L^{OH})Cl**, (e) **Cu(L^{Cl,H})DCA**, and (f) **Cu(L^{Cl,H})Cl** with amino acids in the colchicine pocket. Amino acids located within 5 Å distance were selected; amino acids that do not establish interactions are represented in line format, a potential interaction (magenta dashed line) between the central ion and ASN258. The 2D representation of all the intermolecular interactions between colchicine binding site and (g) **Cu(L^{H})DCA**, (h) **Cu(L^{H})Cl**, (i) **Cu(L^{OH})DCA**, (j) **Cu(L^{OH})Cl**, (k) **Cu(L^{Cl,H})DCA**, and (l) **Cu(L^{Cl,H})Cl**. Green dashed lines represent hydrogen bonds. All the other dashed lines represent various types of π interactions. Light green colored circles indicate the residues involved in van der Waals interactions.

Table S1. The binding affinities as predicted by the scoring functions for the tubulin-colchicine. LOC is the co-crystallized ligand. LOC was re-docked and reproduced the crystal well with low RMSD (Root Mean Square Distance). Physicochemical properties of studied compounds, calculated using swissadme (https://www.swissadme.ch/index.php).

**Compound**	**GS**	**PLP**	**CS**	**ASP**	M_r	**HBA**	**HBD**	**Log** P_c	**ROTB**	**TPSA (Å^2)**
**HL^{H}**	61.9	65.8	30.2	27.4	306.38	2	2	2.45	5	81.4
**HL^{OH}**	61.0	69.1	28.1	30.4	354.38	5	5	0.81	5	142.09
**HL^{Cl,H}**	65.2	65.7	29.3	28.0	375.27	2	2	3.46	5	81.4
**HL**	62.9	67.1	26.0	29.0	396.46	5	2	1.51	8	109.09
**Cu(L^{H})DCA**	65.4	-	-	-	496.86	3	1	2.87	5	93.28
**Cu(L^{H})Cl**	64.6	-	-	-	404.38	1	1	3.09	2	66.98
**Cu(L^{OH})DCA**	70.0	-	-	-	544.86	6	4	1.31	5	153.97
**Cu(L^{OH})Cl**	60.1	-	-	-	452.37	4	4	1.46	2	127.67
**Cu(L^{Cl,H})DCA**	71.5	-	-	-	565.75	3	1	3.84	5	93.28
**Cu(L^{Cl,H})]Cl**	66.6	-	-	-	473.27	1	1	4.09	2	66.98
**1**	77.1	-	-	-	586.93	6	1	1.96	8	120.97
**2**	69.1	-	-	-	494.45	4	1	2.14	5	94.67
**LOC**	**68.9**	**70.3**	**28.3**	**28.5**	399.44	6	1	1.02	6	83.09
RMSD	0.3039	0.2820	0.3186	0.32	-	-	-	-	-	-

Required parameters to fulfill physicochemical properties important for bioavailability as judged appropriate according to Lipinski's Rules: molecular mass (M_r) ≤ 500, partition coefficient (log P_{calc}) ≤ 5, number of hydrogen bond acceptors (HBA) ≤10, number of hydrogen bond donors (HBD) ≤5, number of rotatable bonds ≤10, and topological polar surface area (TPSA) ≤140 Å^2.[1–3]

R2 RNR

Table S2. The binding affinities of Schiff bases as predicted by the scoring functions for the R2 RNR.

**Compound**	**GS**	**PLP**	**CS**	**ASP**
**HL^{H}**	43.2	53.0	29.3	37.4
**HL^{OH}**	48.2	55.3	25.3	37.5
**HL^{Cl,H}**	45.8	56.2	30.4	31.9
**HL**	48.9	60.9	26.5	30.6
**Cu(L^{H})DCA**	55.3	-	-	-
**Cu(L^{H})Cl**	47.7	-	-	-
**Cu(L^{OH})DCA**	54.6	-	-	-
**Cu(L^{OH})Cl**	53.6	-	-	-
**Cu(L^{Cl,H})DCA**	59.4	-	-	-
**Cu(L^{Cl,H})]Cl**	49.3	-	-	-
**1**	52.2	-	-	-
**2**	54.9	-	-	-

Molecular docking in R2 RNR revealed that organic compounds fit well into the protein pockets, Figure S7 highlights the binding mode in the pockets.

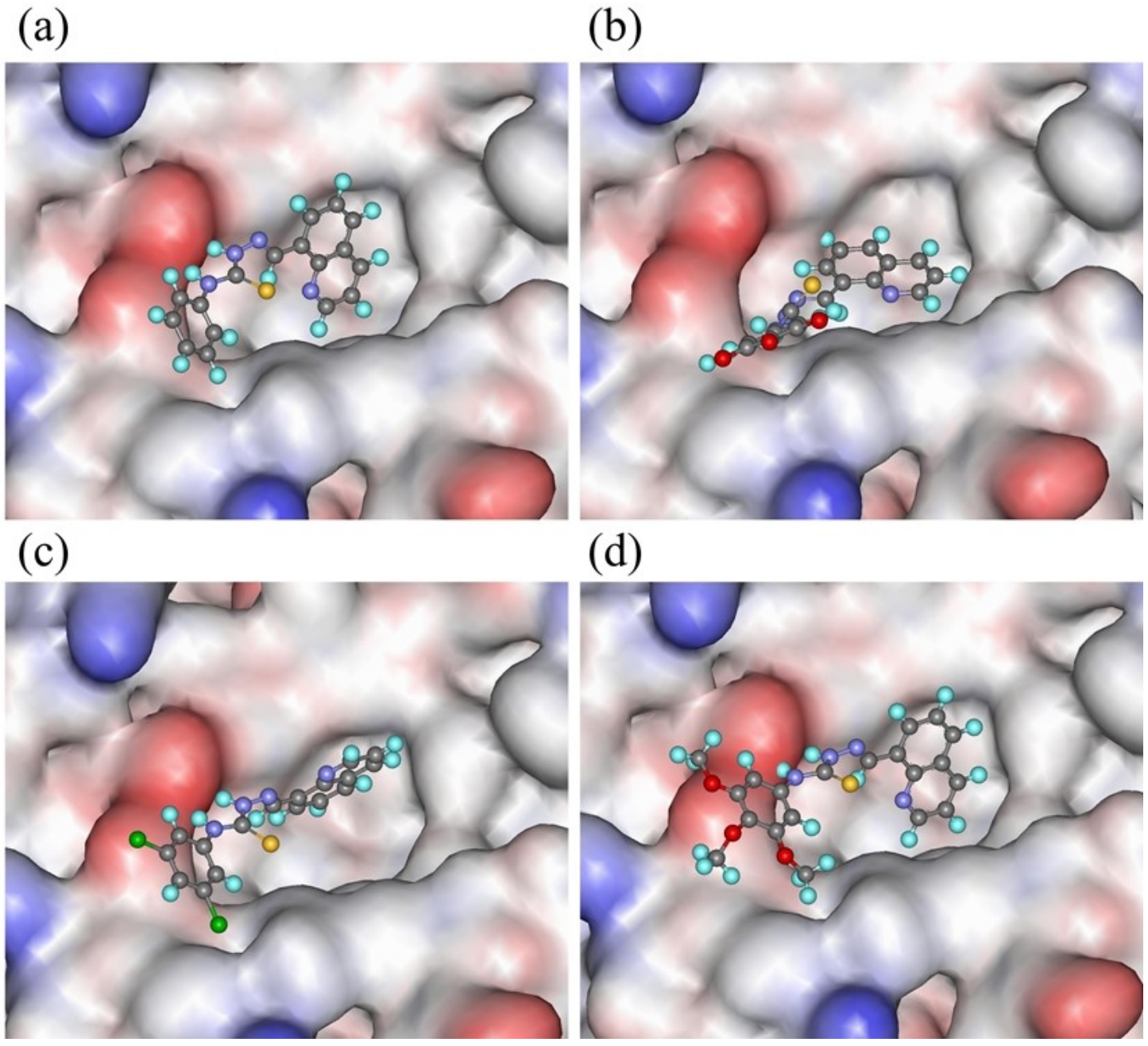

Figure S7. The docked poses of (a) **HL^{H}**, (b) **HL^{OH}**, (c) **HL^{Cl,H}**, and (d) **HL** (ball-and-stick plot). The protein surface is rendered; blue color depicts regions with a partial positive charge on the surface, red color indicates regions with a partial negative charge and grey shows neutral areas.

From the point of view of interactions with amino acids in the composition of proteins, it was predicted that N-H···O hydrogen bonds can be established between the NH groups of the compounds and the amino acid ASP272. The D···A separations are in the range of 1.5–2.3 Å. The exception is the compound **HL^{OH}** which forms a H-bond with ARG331, Figure S8.

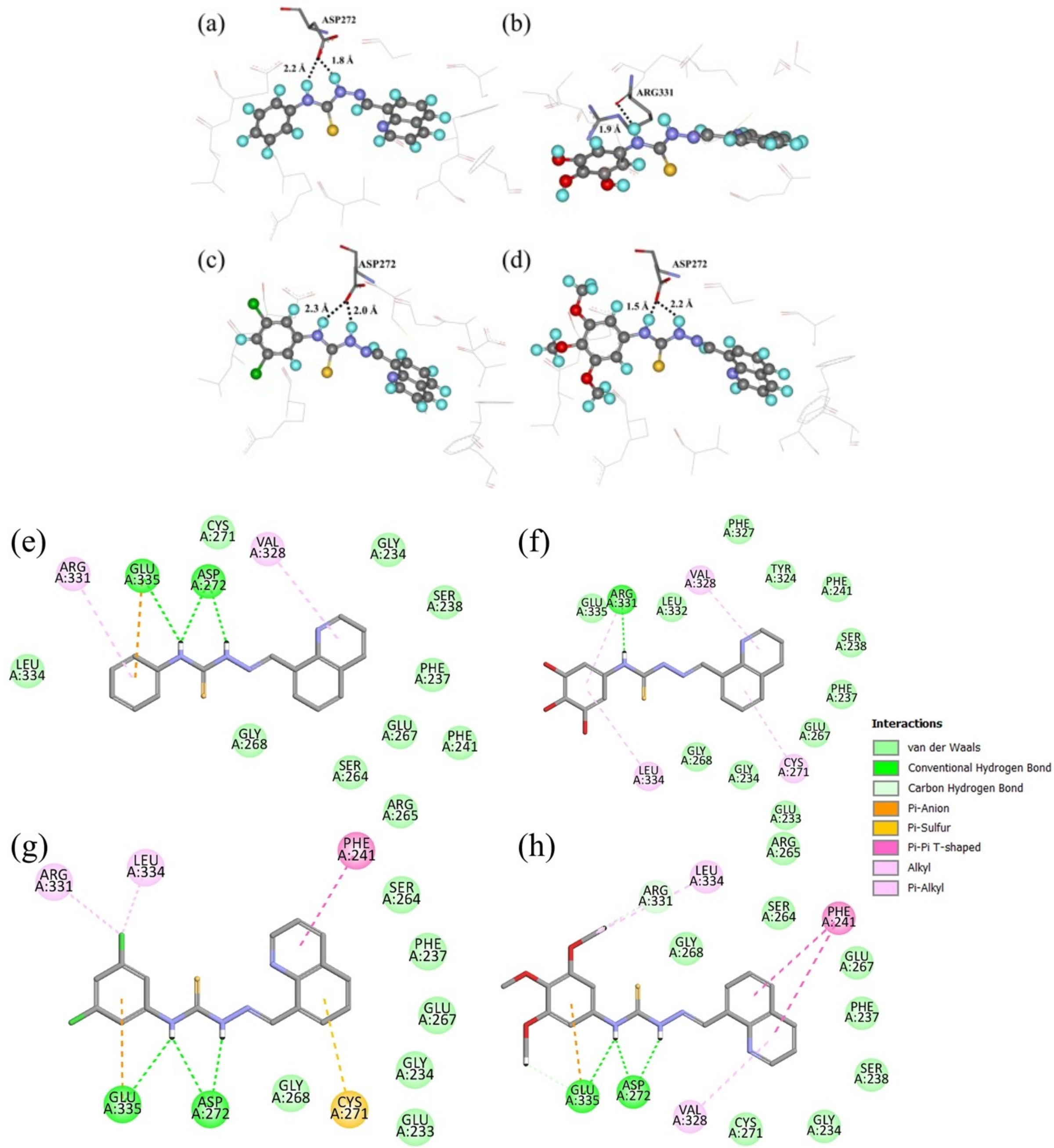

Figure S8. Predicted bindings for (a) **HL^{H}**, (b) **HL^{OH}**, (c) **HL^{Cl,H}**, and (d) **HL** (ball-and stick plot) with amino acids (capped sticks plot) in the R2 protein pocket. H-bonding is represented as black dashed line, while amino acids not involved in intermolecular H-bonding interactions are displayed in wire frame style. The 2D representation of all the intermolecular non-covalent interactions between R2 protein and (e) **HL^{H}**, (f) **HL^{OH}**, (g) **HL^{Cl,H}**, and (h) **HL**. Green dashed lines represent hydrogen bonds. All the other dashed lines represent various types of π interactions specified in the inset to lower panel of the figure. Light green colored circles indicate the residues involved in van der Waals interactions.

In the case of the complexes, good accommodation in the protein pockets was also observed, Figure S9.

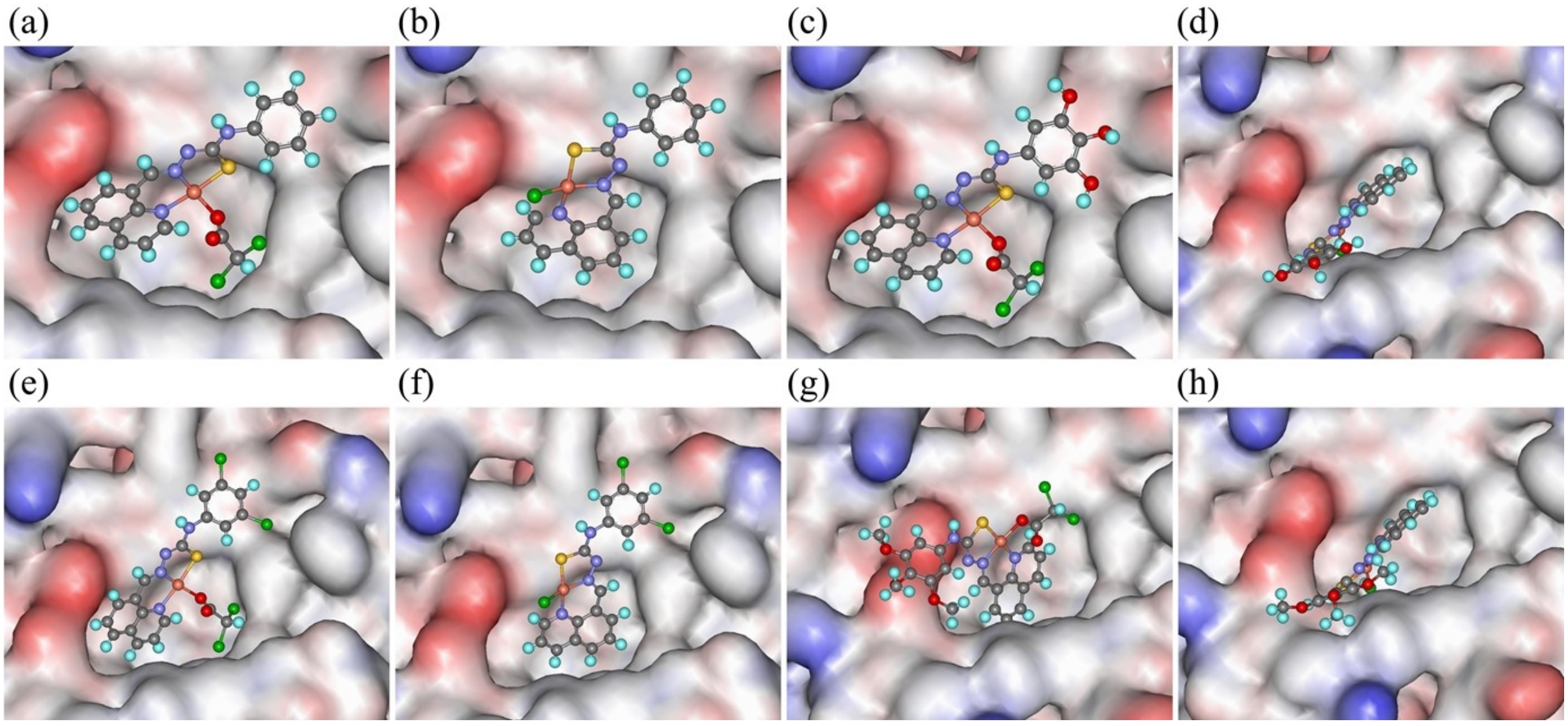

Figure S9. The docked poses of (a) **Cu(L^{H})DCA**, (b) **Cu(L^{H})Cl**, (c) **Cu(L^{OH})DCA**, (d) **Cu(L^{OH})Cl**, (e) **Cu(L^{Cl,H})DCA**, (f) **Cu(L^{Cl,H})]Cl**, (h) **1**, and (h) **2** (ball-and-stick plot). The protein surface is rendered; blue color depicts regions with a partial positive charge on the surface, red color indicates regions with a partial negative charge and grey shows neutral areas.

Regarding the interactions established with the protein, for the complexes N/O···Cu interaction with the amino acid GLY268 was predicted, with a non-bonding contact of 3.7–3.9 Å. It was also predicted that **Cu(L^{OH})Cl** and **2** are not prone to establish interactions with the metal center, in these cases an N–H···O hydrogen bond can be formed with the amino acid ARG331 with D···A separations of 2.1 and 1.8 Å, respectively, Figure S10.

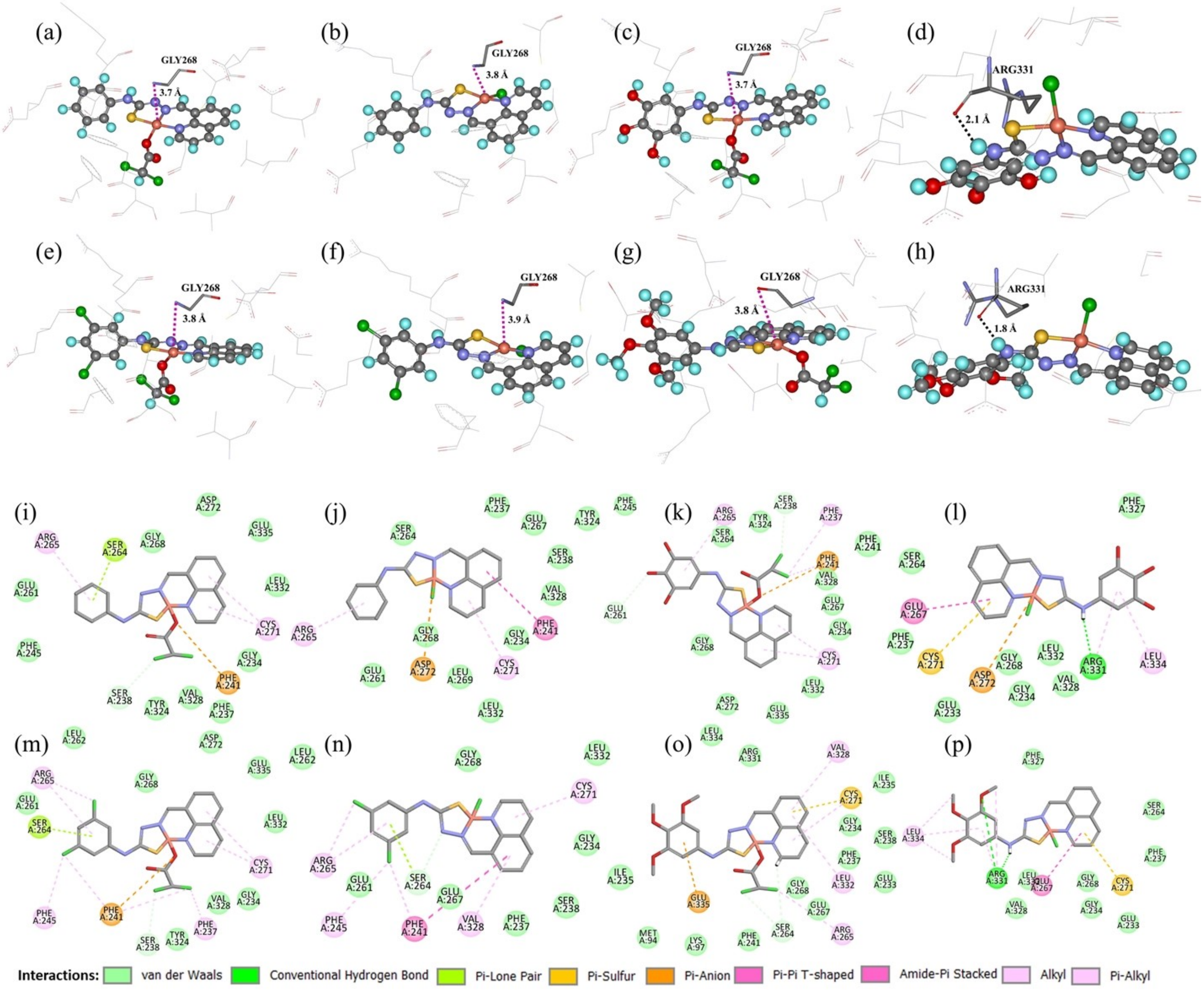

Figure S10. Predicted bindings for ((a) **Cu(L^{H})DCA**, (b) **Cu(L^{H})Cl**, (c) **Cu(L^{OH})DCA**, (d) **Cu(L^{OH})Cl**, (e) **Cu(L^{Cl,H})DCA**, (f) **Cu(L^{Cl,H})]Cl**, (g) **1**, and (h) **2** (ball-and stick plot) with amino acids (capped sticks plot) in the protein pocket. Potential contacts between GLY268 located within 5 Å and the central ion are represented as magenta dashed lines, H-bonding as black dashed line, while amino acids that are not involved in a possible binding with Cu(II) are displayed in wire frame style. The 2D representation of all the intermolecular interactions between R2 protein and (i) **Cu(L^{H})DCA**, (j) **Cu(L^{H})Cl**, (k) **Cu(L^{OH})DCA**, (l) **Cu(L^{OH})Cl**, (m) **Cu(L^{Cl,H})DCA**, (n) **Cu(L^{Cl,H})]Cl**, (o) **1**, and (p) **2**. Green dashed lines represent hydrogen bonds. All the other dashed lines represent various types of π interactions specified on the bottom of the lower panel of the figure. Light green colored circles indicate the residues involved in van der Waals interactions.

Molecular modeling calculations. All the compounds shown in Chart S1 were docked into the binding sites of tubulin-colchicine (PDB ID: 4O2B, resolution 2.30 Å)[4] and mR2 RNR (PDB ID:1SA0, resolution 3.58 Å),[5] which were obtained from the Protein Data Bank (PDB).[6,7] The structures **HL^{H}**, **HL^{OH}**, **HL^{Cl,H}**, **HL**, **Cu(L^{H})DCA**, **Cu(L^{H})Cl**, **Cu(L^{OH})DCA**, **Cu(L^{OH})Cl**, **Cu(L^{Cl,H})DCA**, **Cu(L^{Cl,H})Cl** were built using HyperChem (8.0.7) and optimized using the MM+ force field.[8] The crystal structures of the complexes **1** and **2** were used. The GOLD (v2025.3) software suite was used to prepare the crystal structures for docking, i.e., the hydrogen atoms were added, water molecules and expedients deleted and the co-crystallised ligand identified: tubulin-colchicine – LOC (N-[(7*S*)-1,2,3,10-tetramethoxy-9-oxo-6,7-dihydro-5H-benzo[d]heptalen-7-yl]ethanamide). The docking centre for the tubulin-colchicine binding pocket was defined as the position of the co-crystallised LOC ligands and for mR2 RNR the center was defined (x = 102.276, y = 87.568, z = 80.588) close to Fe_2O and the enzymatically essential tyrosyl residue (Tyr177),[9] both with 10 Å radius. The scoring functions GoldScore (GS),[10] ChemScore(CS),[11,12] Piecewise Linear Potential (ChemPLP)[13] and Astex Statistical Potential (ASP)[14] was used in the GOLD (v2025.3) docking algorithm.

ESI mass spectra and NMR spectra

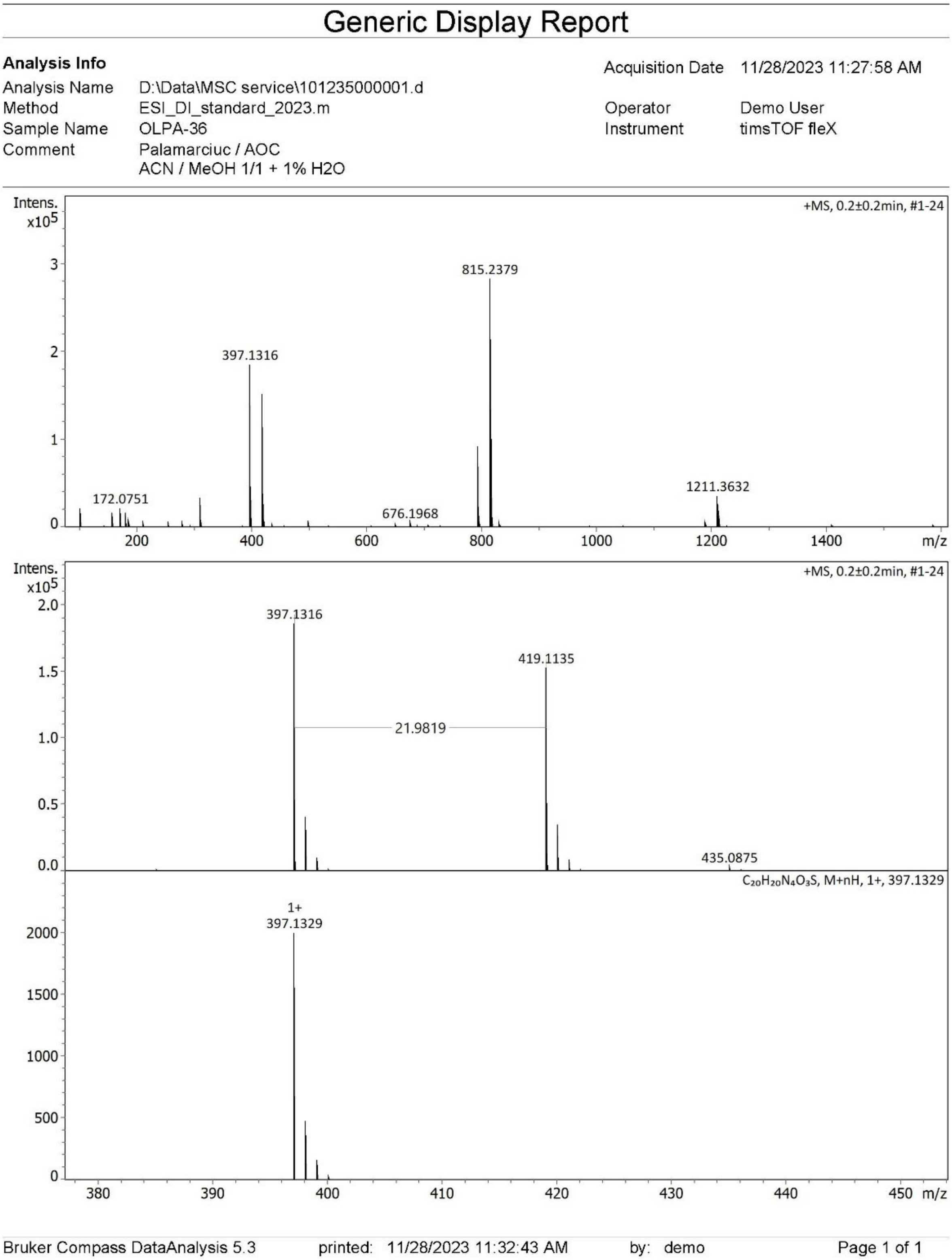

Figure S11. High resolution ESI(+) mass spectrum of **HL**.

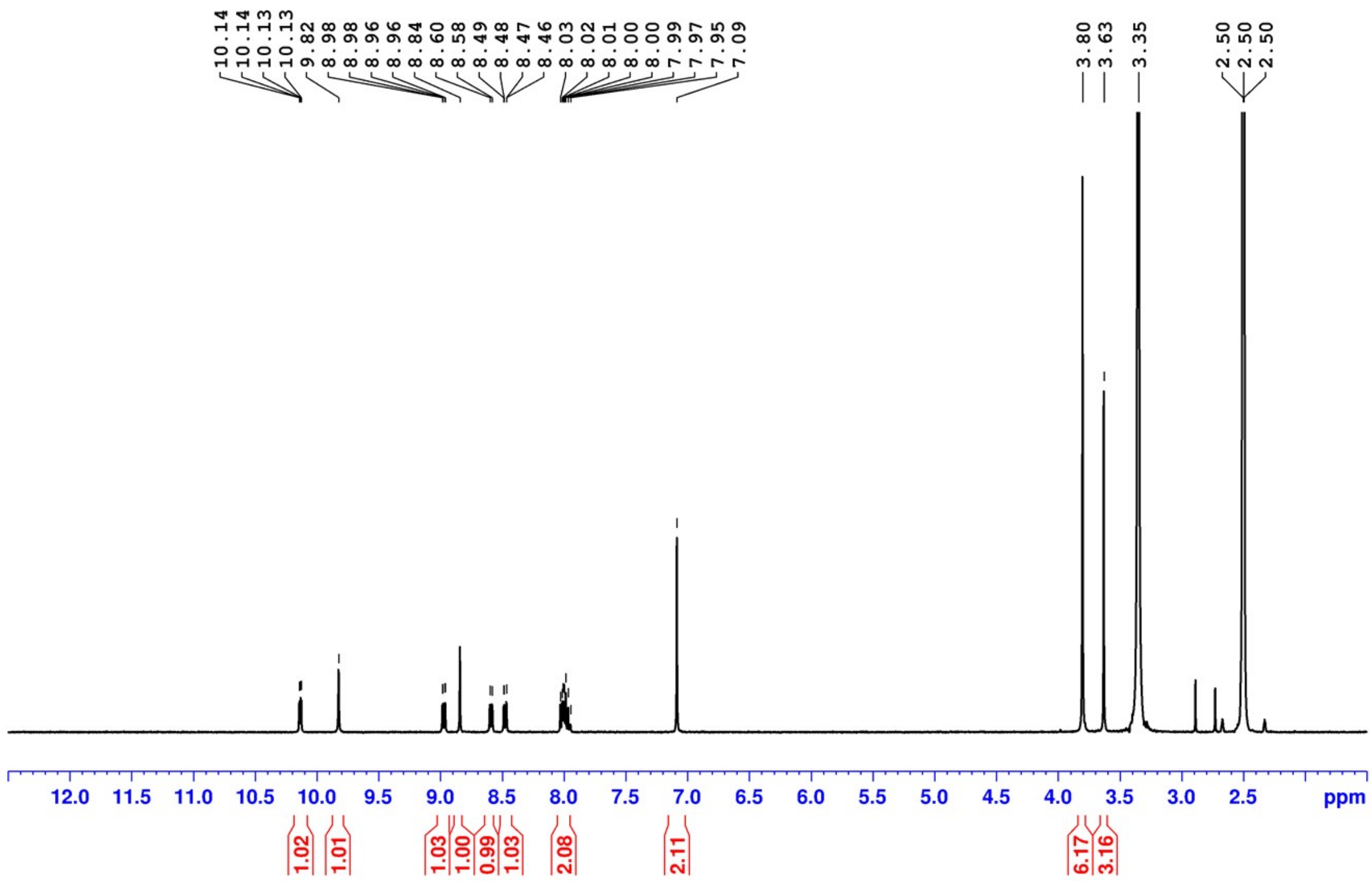

Figure S12. ^{1}H NMR spectrum of **HL**.

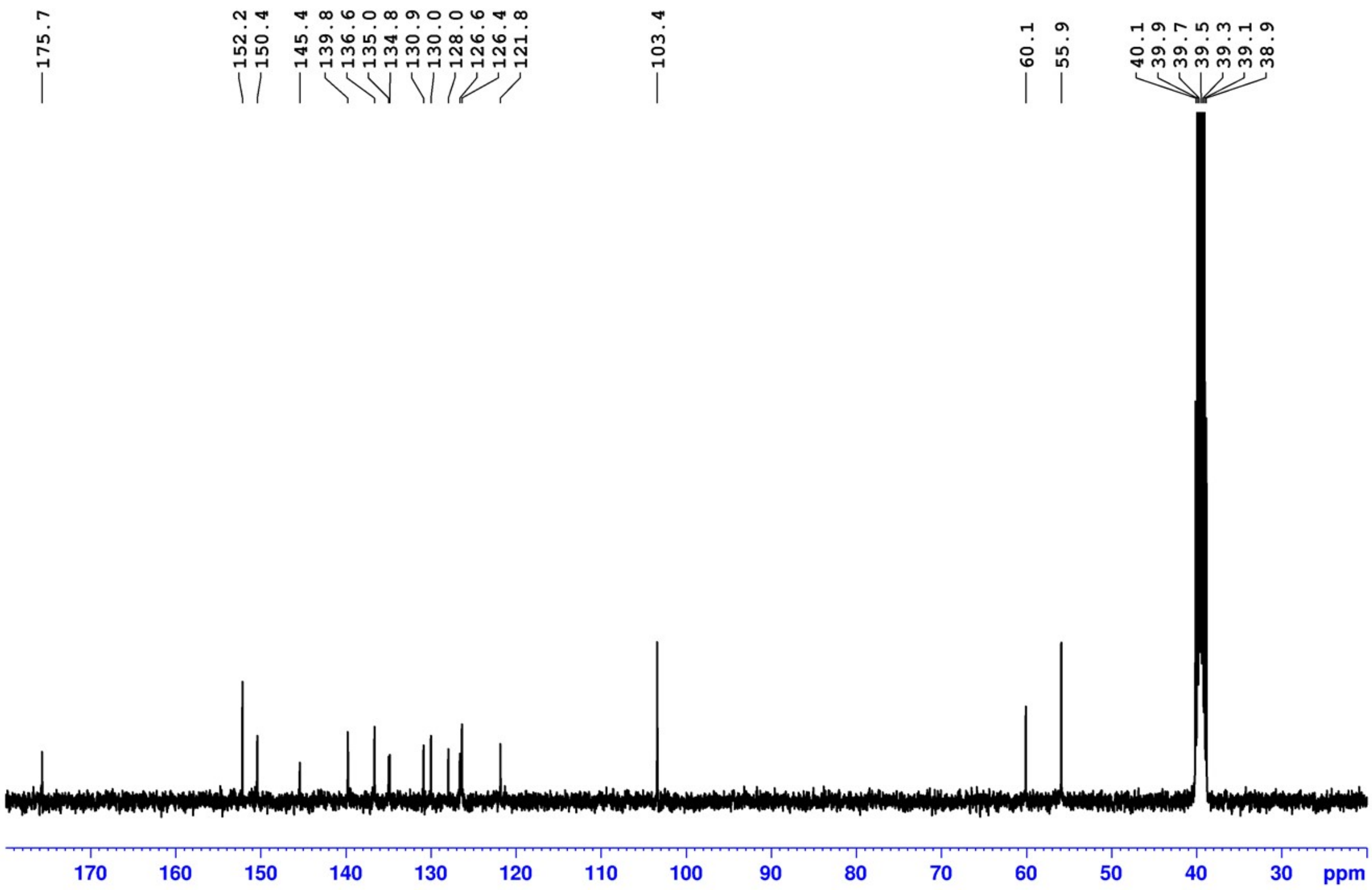

Figure S13. ^{13}C NMR spectrum of **HL**.

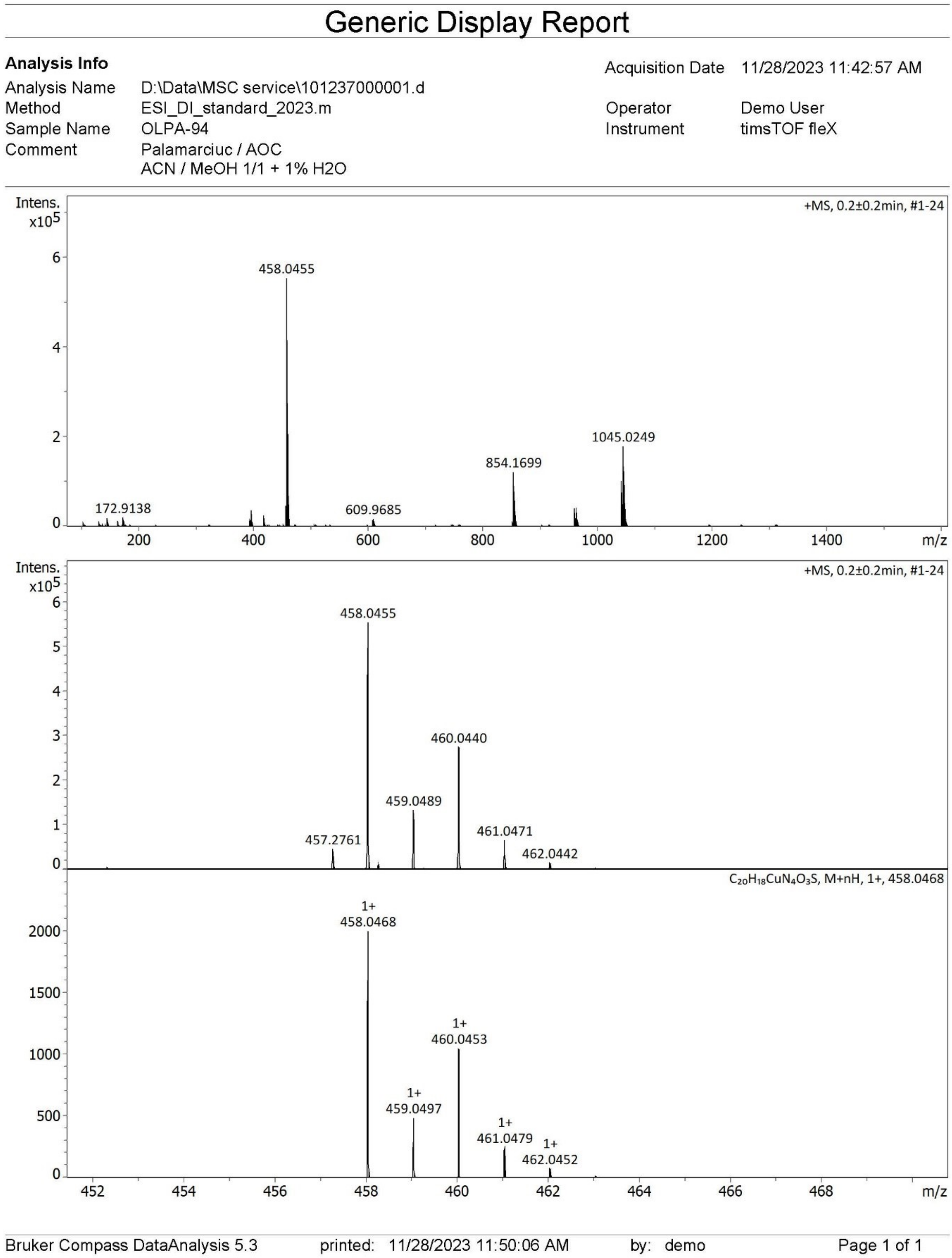

Figure S14. HR ESI(+) mass spectrum of **[Cu(HL)(Cl_2CHCOO)]Cl_2CHCOO (1′)**.

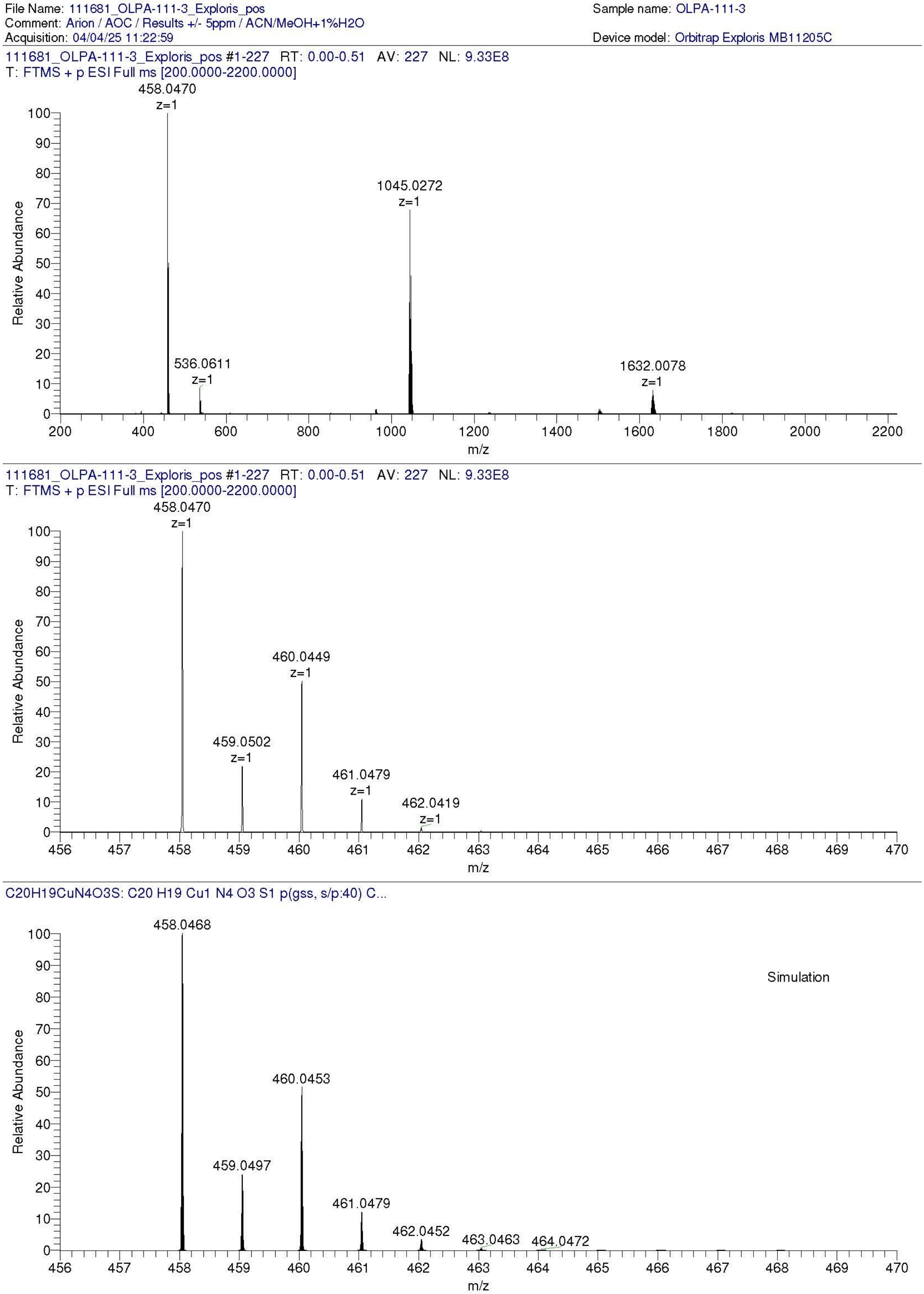

Figure S15. High resolution ESI(+) mass spectrum of **1·DMSO·H_2O**.

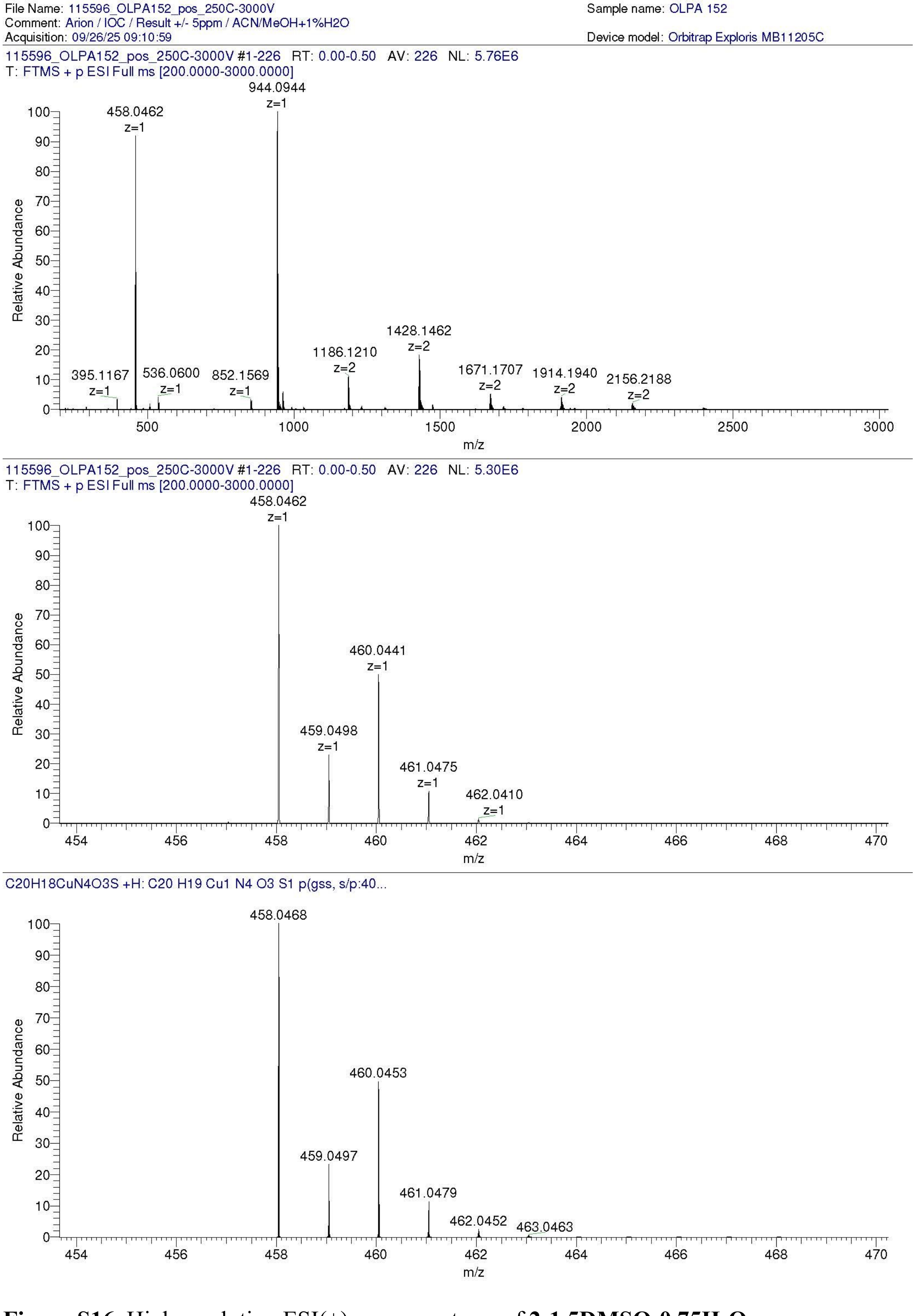

Figure S16. High resolution ESI(+) mass spectrum of **2·1.5DMSO·0.75H$_2$O**.

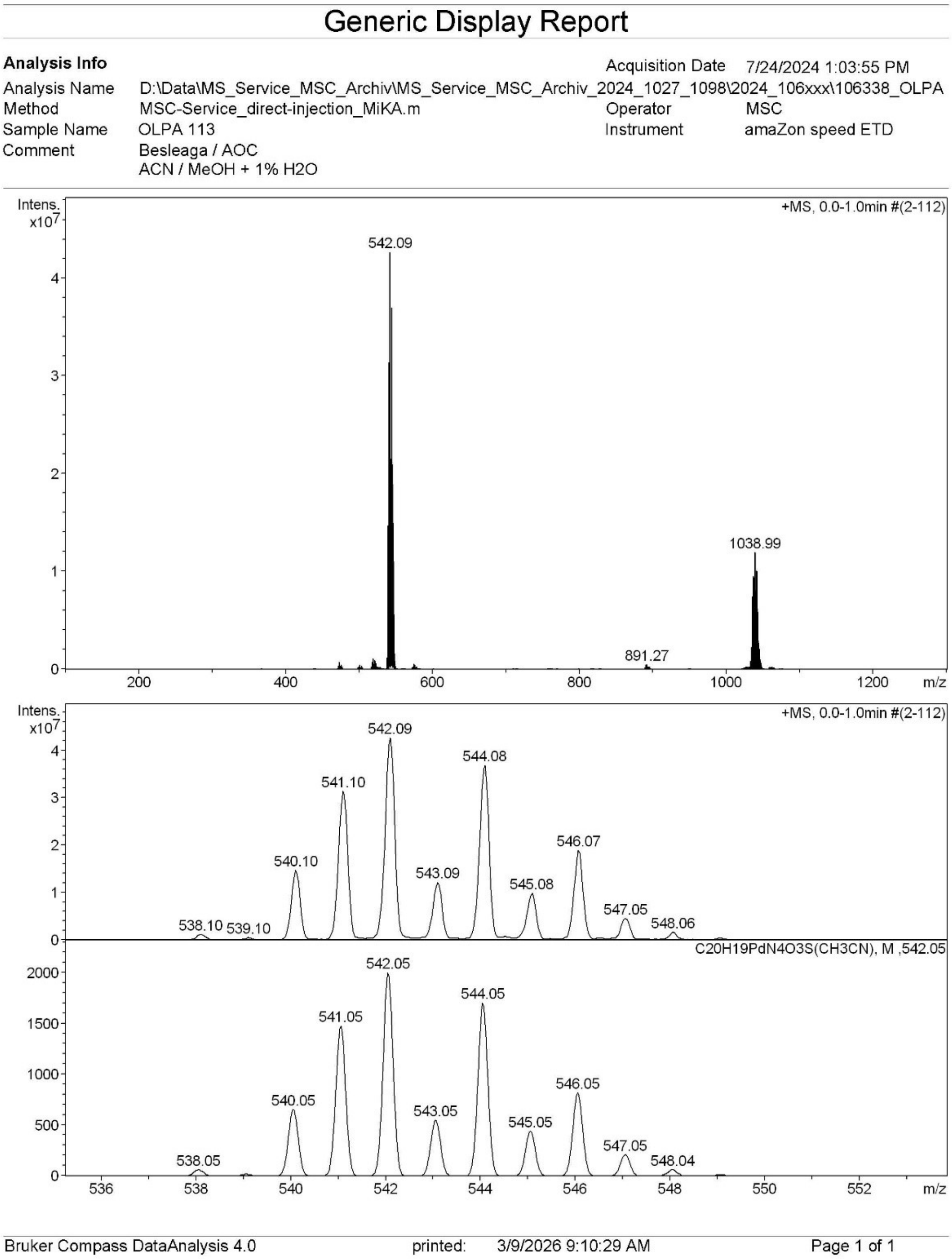

Figure S17. Low resolution ESI(+) mass spectrum of Pd(II) complex **3** with simulation of the peak with *m*/*z* 542.09.

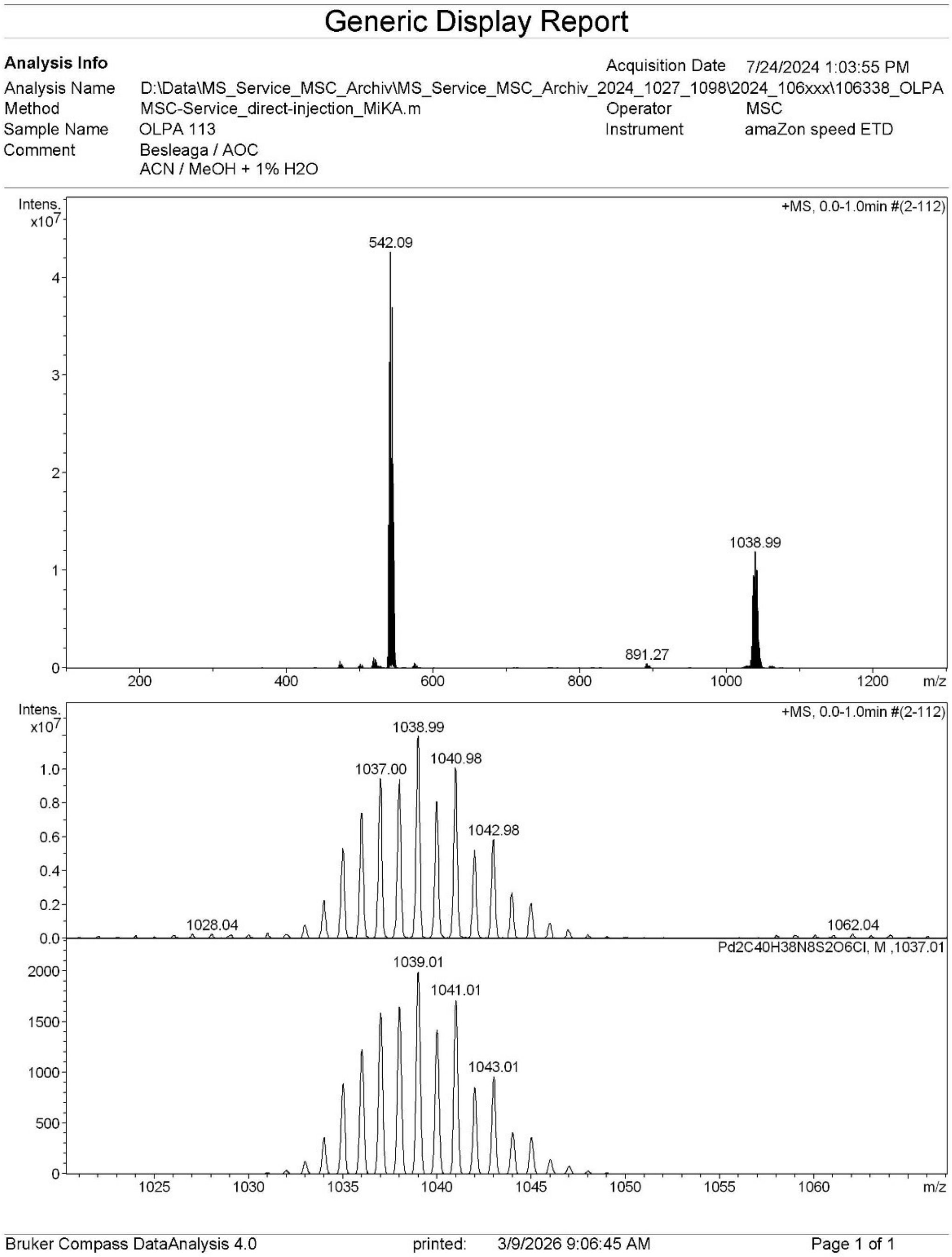

Figure S18. Low resolution ESI(+) mass spectrum of Pd(II) complex **3** with simulation of the peak with *m*/*z* 1038.99.

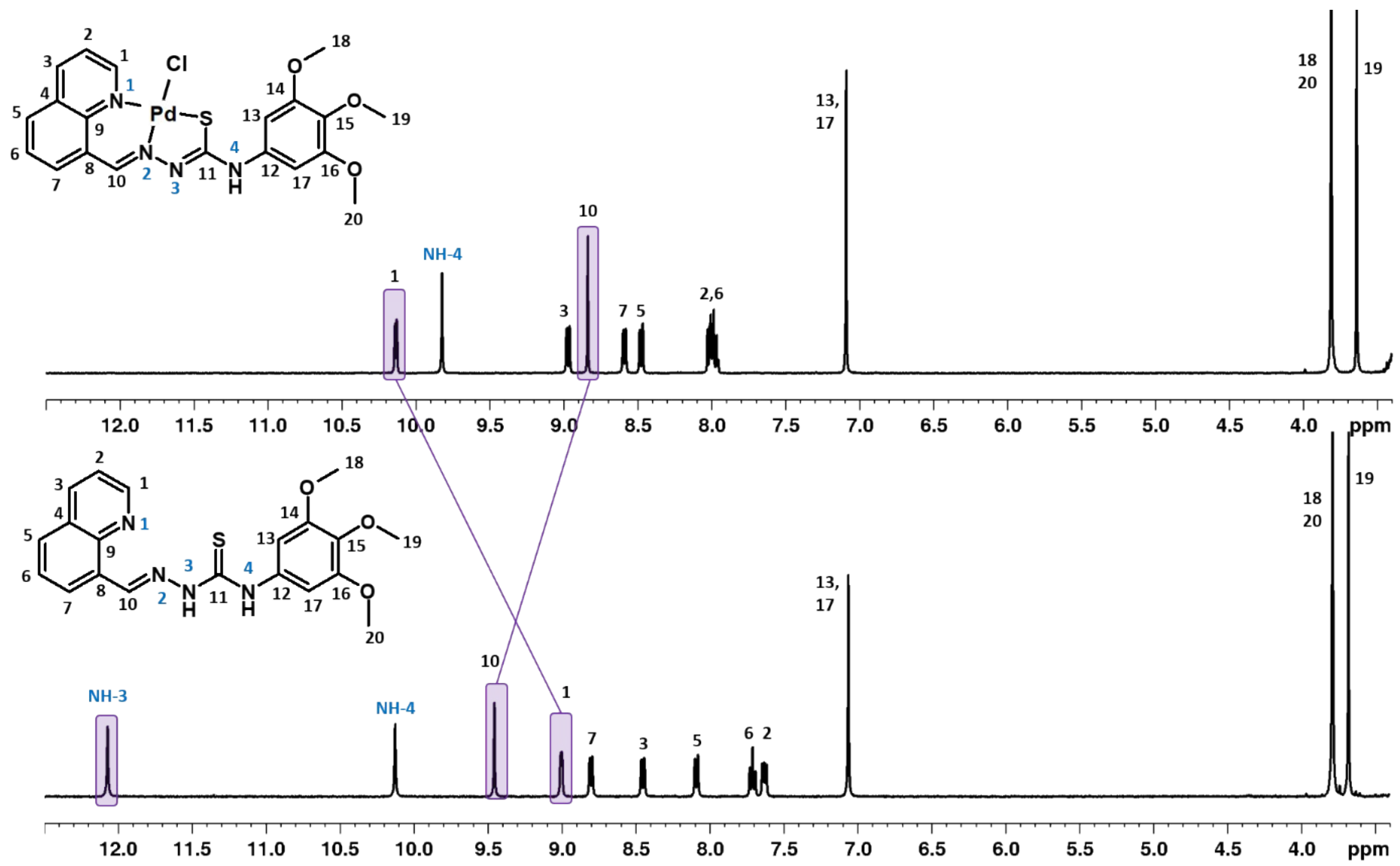

Figure S19. Comparison of the ^{1}H NMR spectra of **HL** and **PdLCl (3)**.

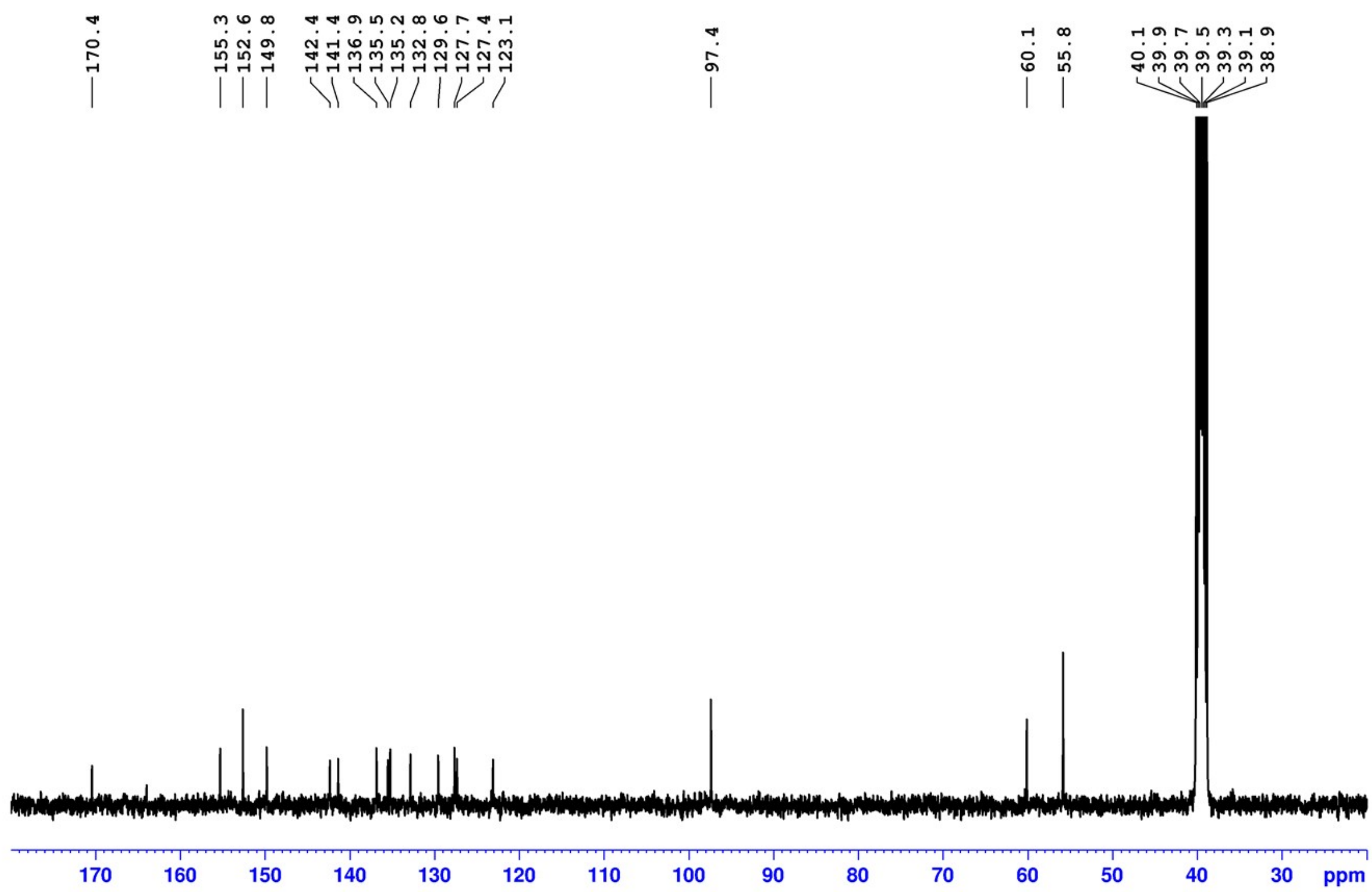

Figure S20. ^{13}C NMR spectrum of **3**.

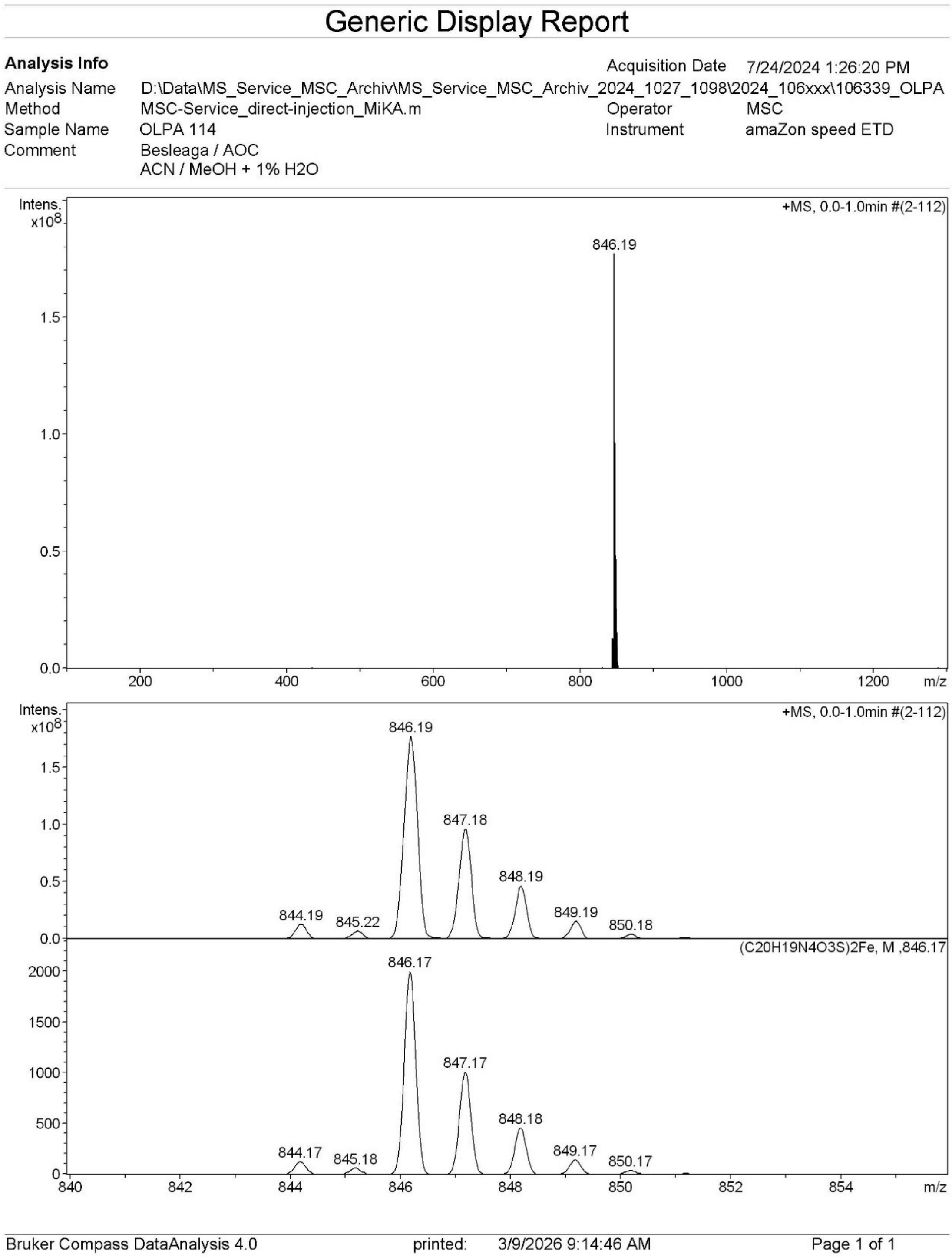

Figure S21. Low resolution ESI(+) mass spectrum of Fe(III) complex **4**.

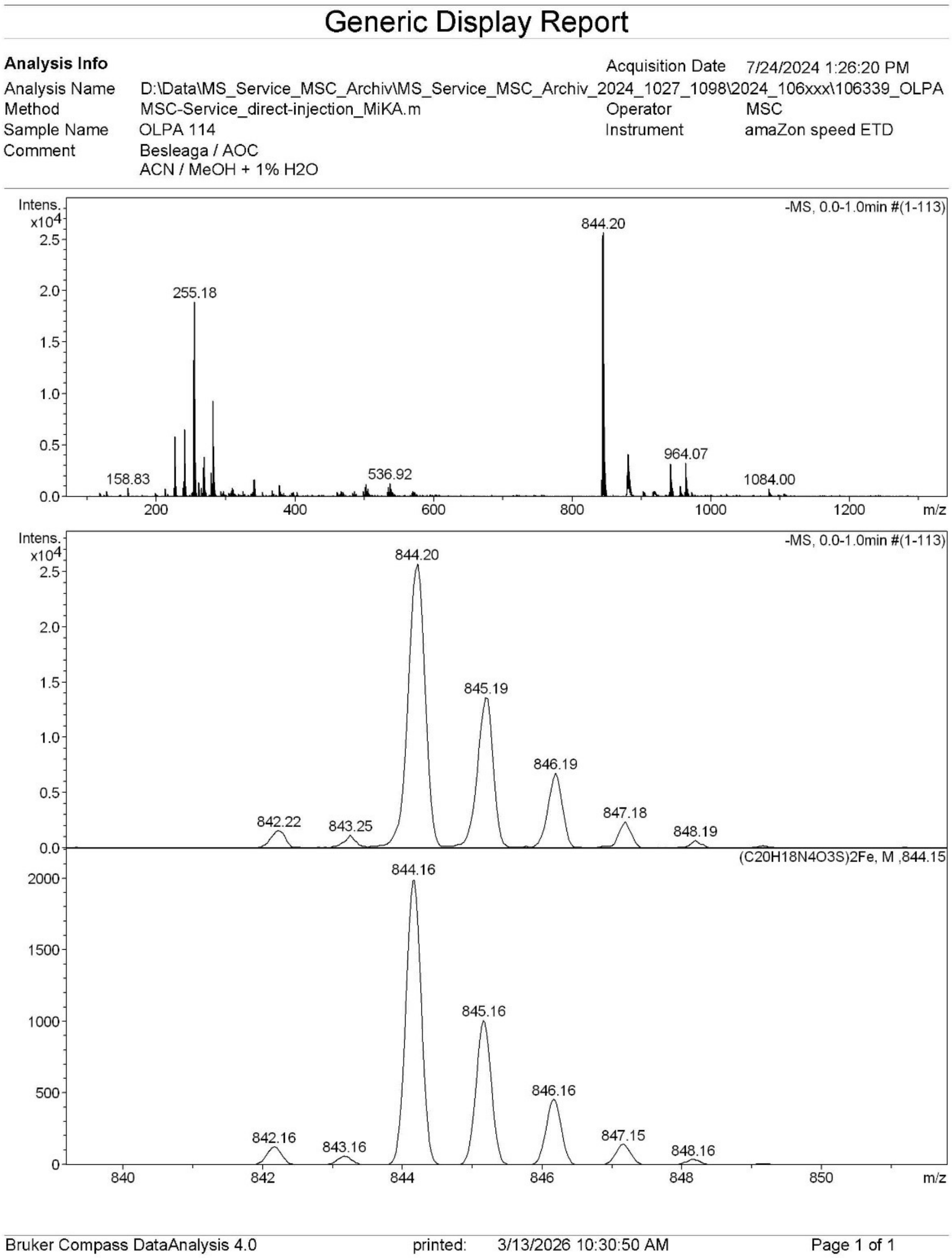

Figure S22. Low resolution ESI(−) mass spectrum of Fe(III) complex **4**.

IR spectra

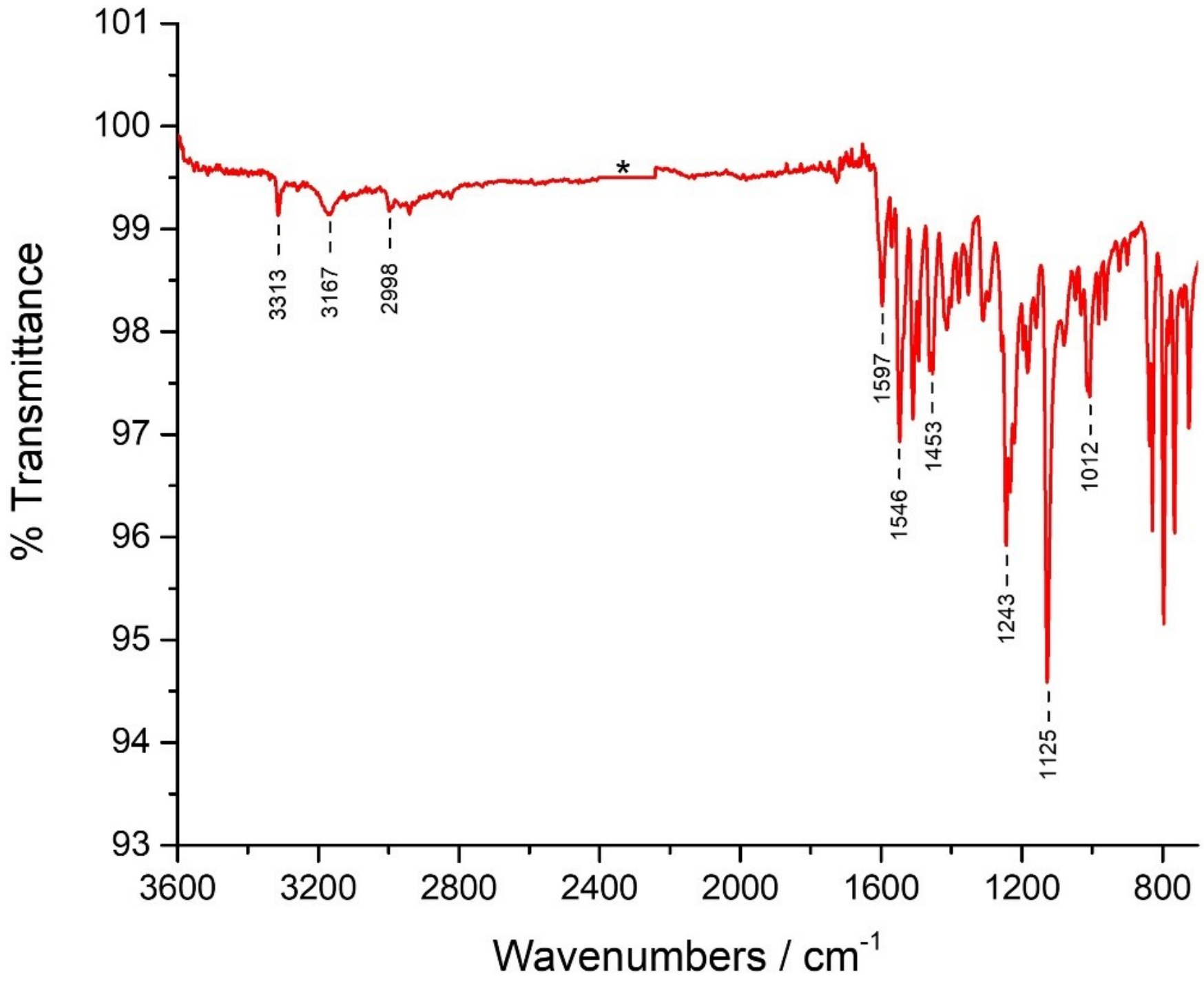

Figure S23. IR spectrum of **HL**.

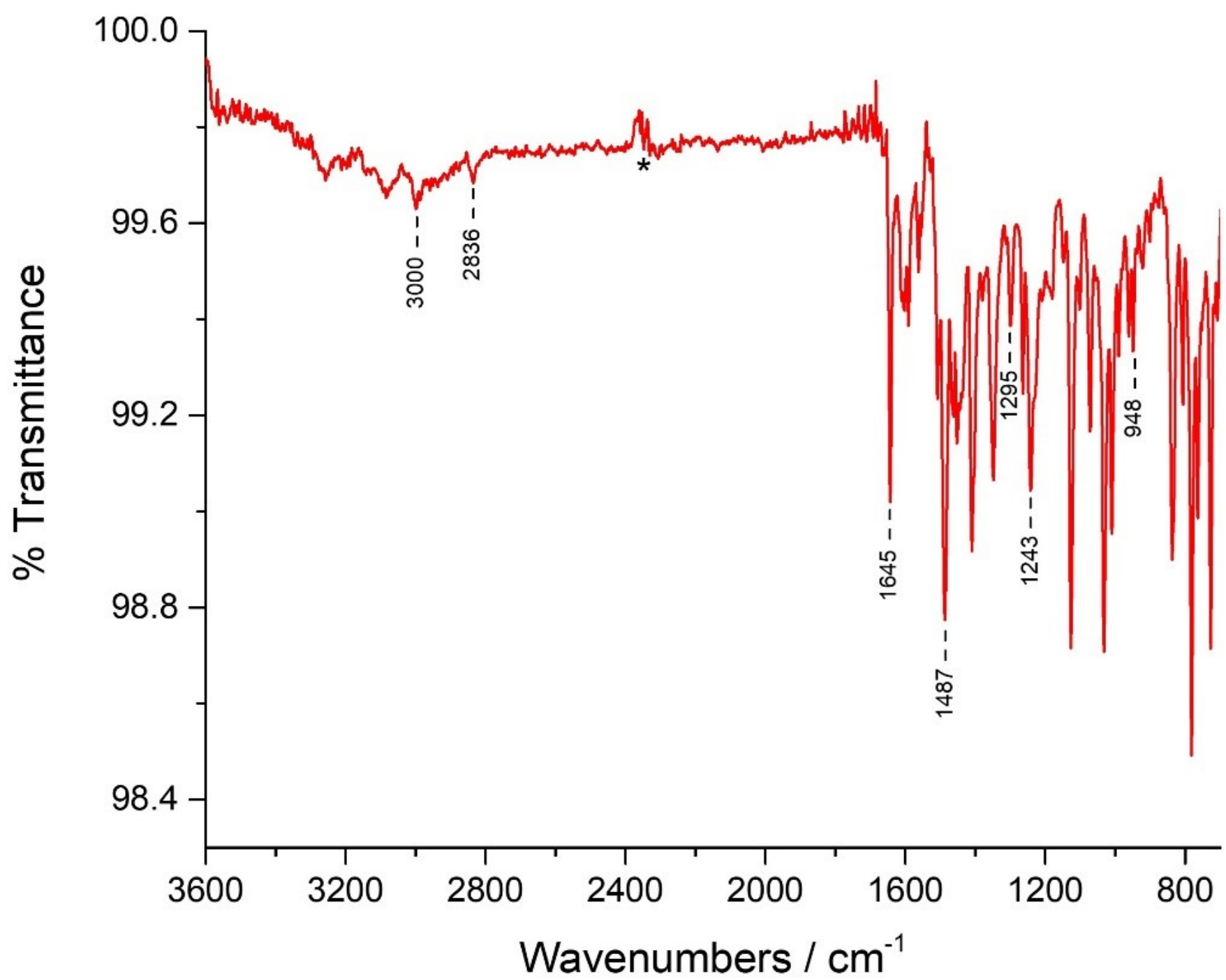

Figure S24. IR spectrum of **1**.

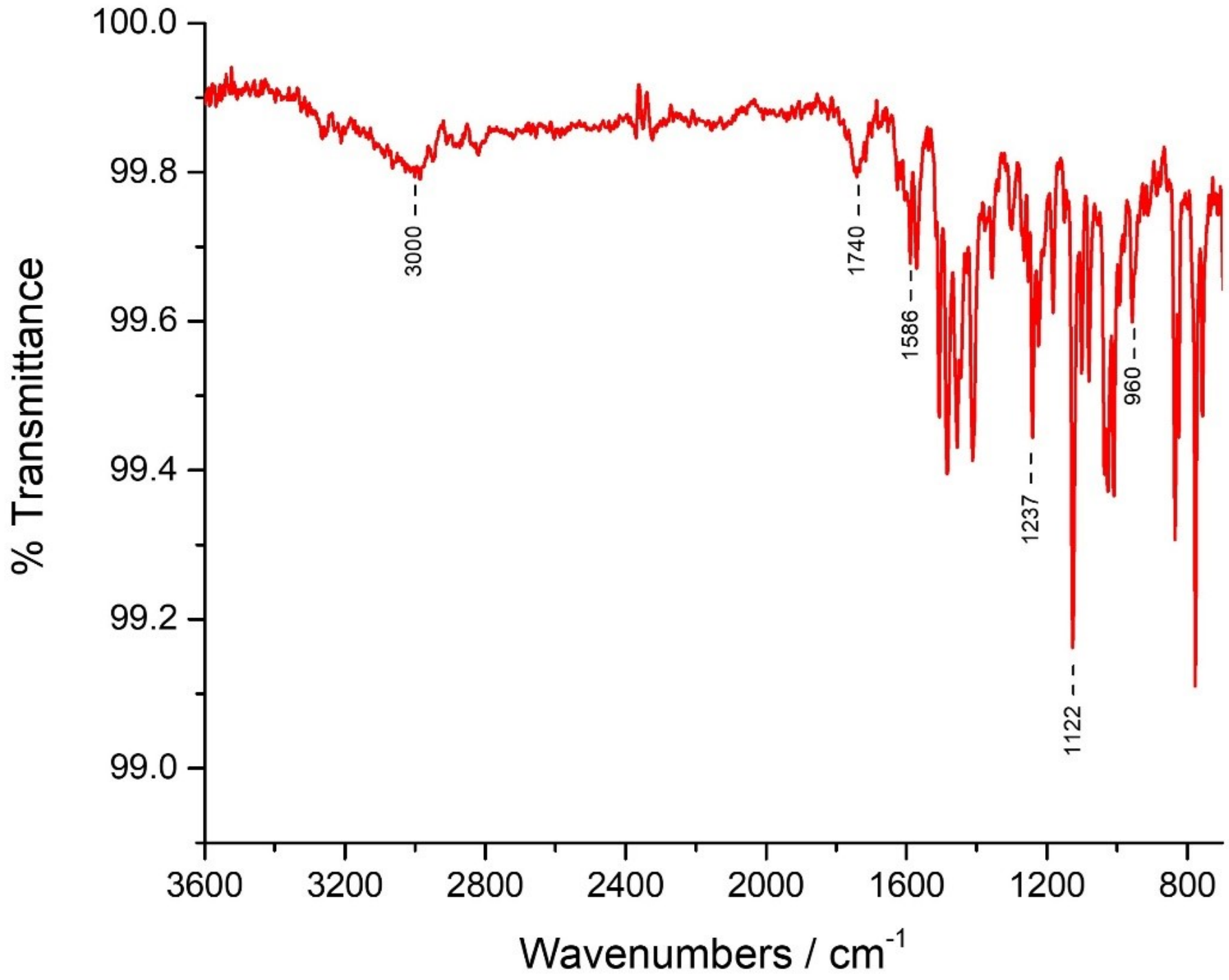

Figure S25. IR spectrum of **2**.

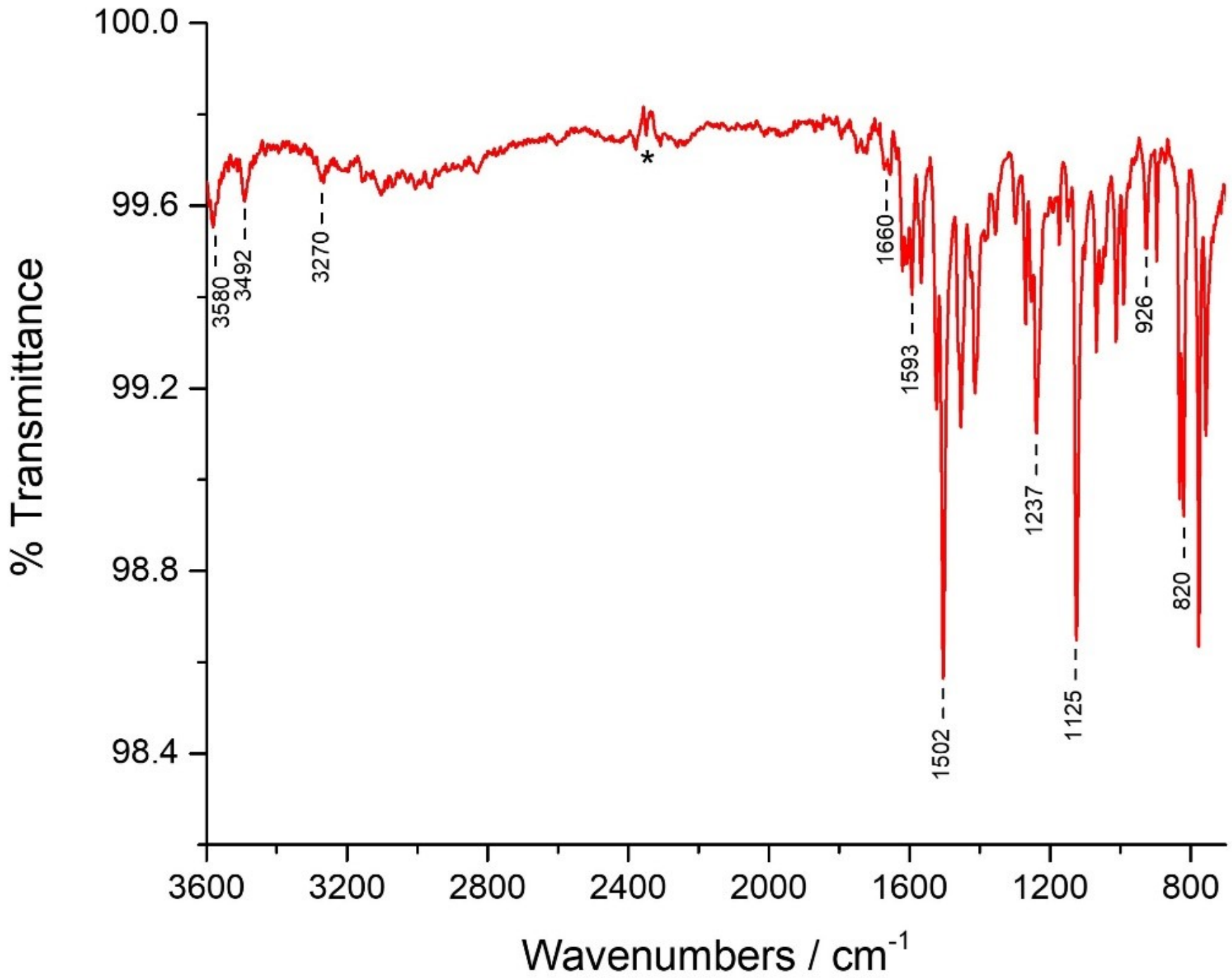

Figure S26. IR spectrum of **3**.

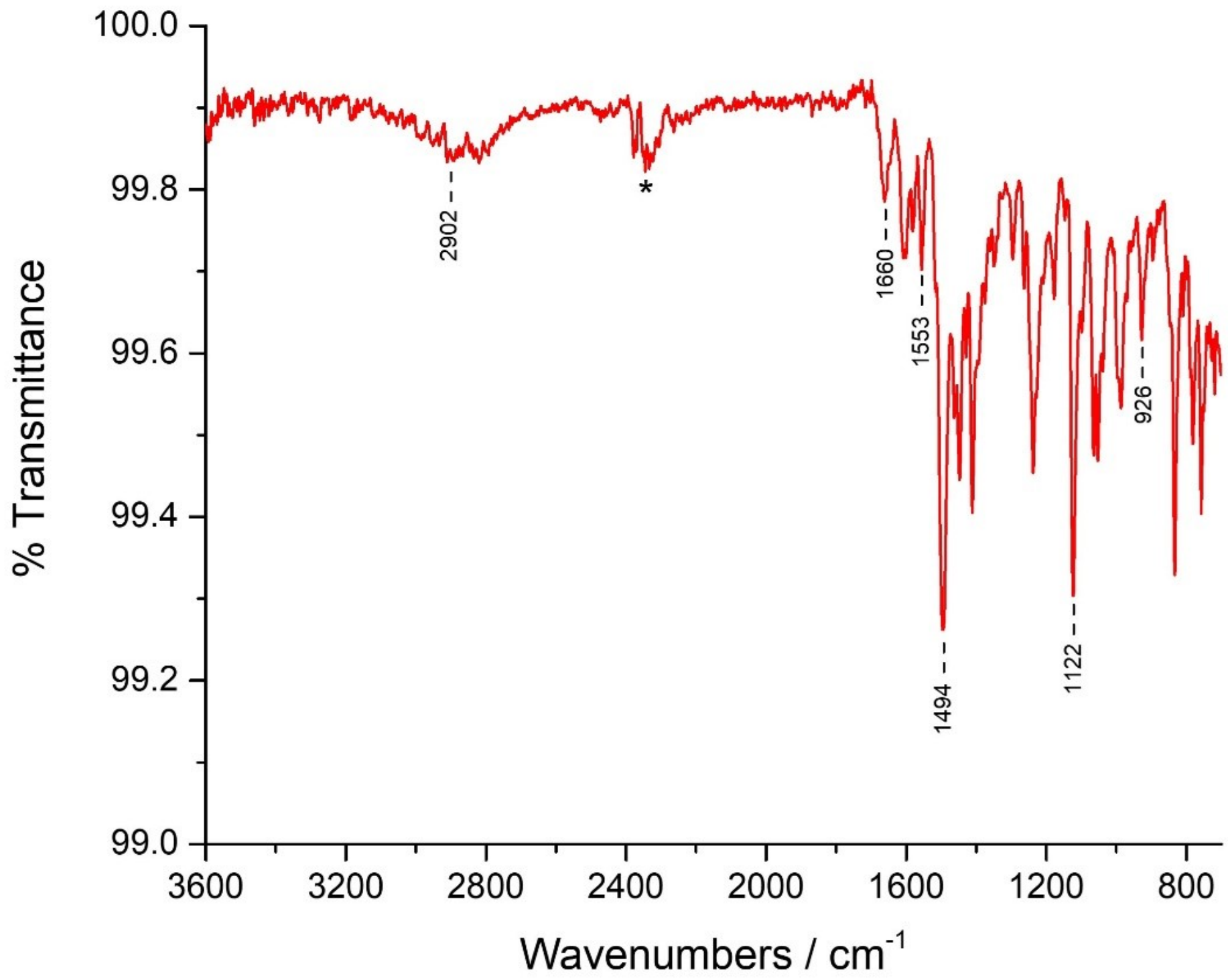

Figure S27. IR spectrum of **4**.

X-ray diffraction data

Table S3. Details of Data Collection and refinement for metal complexes studied

Compound	**1·DMSO·0.5H$_2$O**	**2·DMSO**	**3·0.5H$_2$O**	**4·1.5H$_2$O**
empirical formula	$C_{48}H_{54}Cl_4Cu_2N_8O_{13}S_4$	$C_{22}H_{25}ClCuN_4O_4S_2$	$C_{40}H_{40}Cl_2N_8O_7Pd_2S_2$	$C_{40}H_{41}ClFeN_8O_{7.5}S_2$
fw	1348.11	572.57	1092.62	909.23
T [K]	100	100	100	100
space group	*P*2$_1$/n	*P*2$_1$/*c*	*P*-1	*I*2/*a*
a [Å]	13.87419(16)	7.6769(3)	8.00548(17)	16.7360(2)
b [Å]	24.6180(3)	17.8698(6)	15.0778(2)	12.1047(2)
c [Å]	16.69315(19)	17.7731(5)	17.7365(2)	21.1161(3)
α [°]			74.0212(14)	
b [°]	101.3608(12)	96.087(3)	85.5907(15)	99.837(1)
g [°]			80.9691(16)	
V [Å^3]	5589.91(11)	2424.44(15)	2031.37(6)	4214.90(11)
Z	4	4	2	4
ρ_{calcd} [g cm^{-3}]	1.602	1.569	1.786	1.433
μ [mm^{-1}]	4.685	4.216	9.841	4.884
crystal size [mm]	0.30 × 0.03 × 0.02	0.15 × 0.02 × 0.02	0.20 × 0.03 × 0.03	0.30 × 0.10 × 0.05
$R_1{}^a$, wR_2(all data)^b	0.0459, 0.1255	0.0467, 0.1307	0.0319, 0.0861	0.0433, 0.1296
GOF^c	1.028	1.015	1.069	1.089
CCDC no.	2540743	2540744	2540745	2540746

^{a} $R_1 = \Sigma||F_o| - |F_c||/\Sigma|F_o|$. ^{b} $wR_2 = \{\Sigma[w(F_o^2 - F_c^2)^2]/\Sigma[w(F_o^2)^2]\}^{1/2}$. ^{c} GOF = $\{\Sigma[w(F_o^2 - F_c^2)^2]/(n - p)\}^{1/2}$, where n is the number of reflections and p is the total number of parameters refined

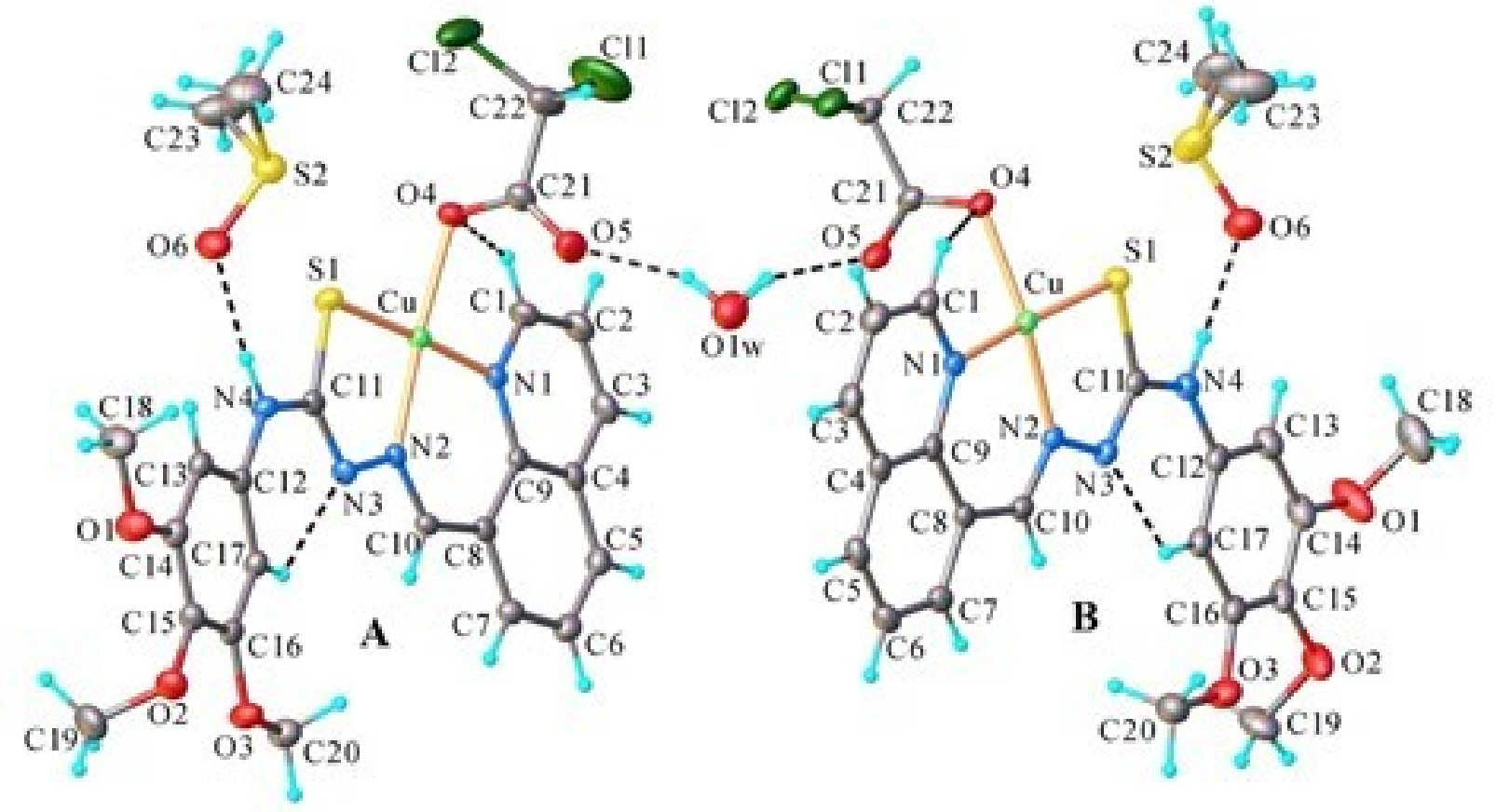

Figure S28. A fragment of the crystal structure of **1** showing two molecules of the complex bridged by one water molecule via H-bonding interactions with parameters collected in Table S3.

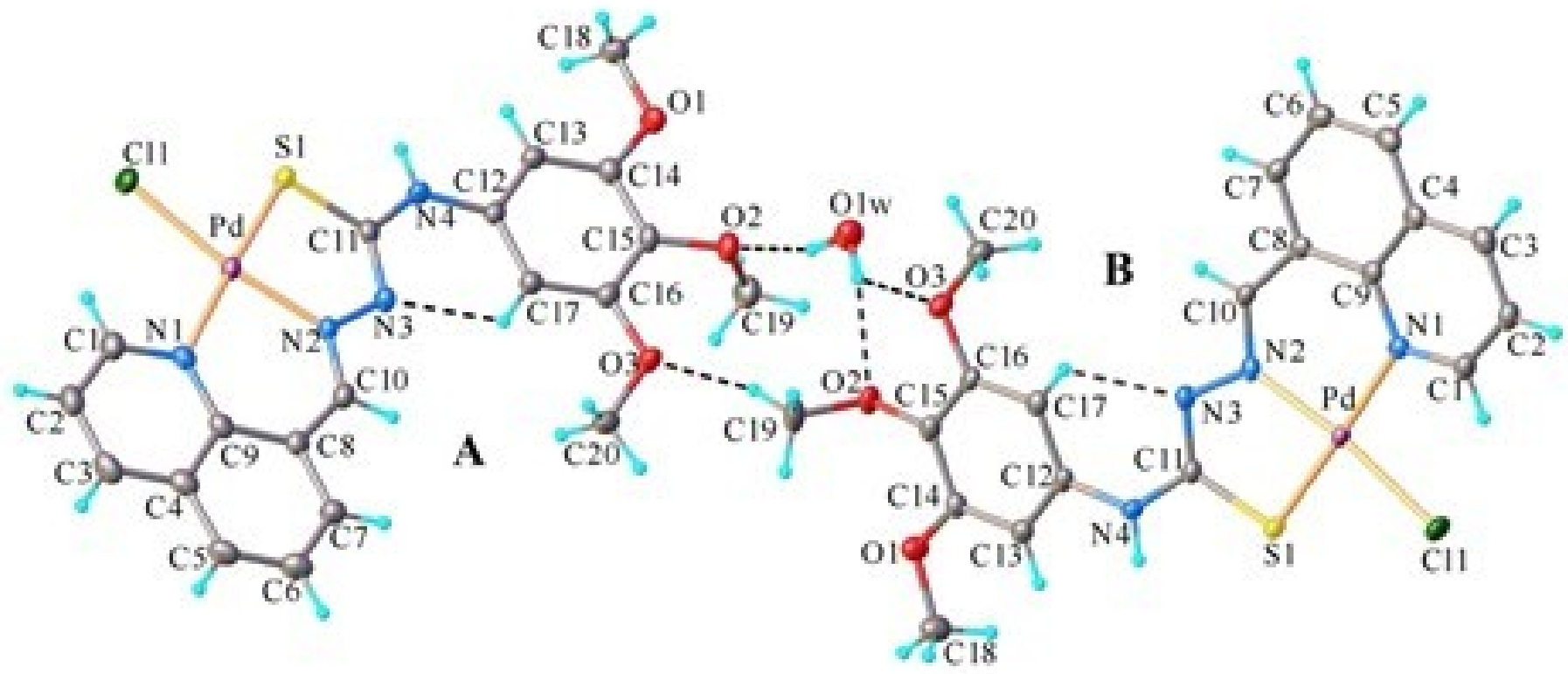

Figure S29. A fragment of the crystal structure of 3 showing hydrogen bonding interactions; the parameters are collected in Table S3.

Table S4. Hydrogen bonding parameters:

1·DMSO·0.5H$_2$O

D–H···A	$d_{D\text{-}H}$/Å	$d_{H\text{-}A}$/Å	$d_{D\text{-}A}$/Å	∠D–H···A/°	Symmetry code
O1*w*–H···O5A	0.87	1.98	2.811(3)	157	-
O1*w*–H···O5B	0.87	2.03	2.839(3)	154	-
N4A–H···O6A	0.88	2.03	2.904(3)	174	-
N4B–H···O6B	0.88	2.04	2.921(3)	177	-
C1A–H···O4A	0.95	2.24	2.883(3)	124	-
C1B–H···O4B	0.95	2.26	2.868(3)	121	-
C17A–H···N3A	0.95	2.21	2.829(3)	121	-
C17B–H···N3B	0.95	2.18	2.826(3)	124	-

2·DMSO

N4–H···O4	0.88	2.00	2.831(3)	157	-
C13–H···O4	0.95	2.53	3.303(4)	138	-
C17–H···N3	0.95	2.26	2.873(4)	121	-

3·0.5H$_2$O

O1*w*–H···O2A	0.88	1.98	2.824(3)	161	-
O1*w*–H···O2B	0.88	2.15	3.005(3)	163	-
O1*w*–H···O3B	0.88	2.66	3.292(3)	130	-
C17A–H···N3A	0.95	2.25	2.872(4)	122	-
C17B–H···N3B	0.95	2.26	2.860(3)	120	-
C19B-–H···O3A	0.98	2.45	3.407(4)	164	-

4·1.5H$_2$O

N4–H···Cl1	0.88	2.26	3.088(2)	156	-
N4–H···Cl1	0.88	2.32	3.079(2)	145	1–*x*, –*y*, 1–*z*
C17–H···N3	0.95	2.26	2.876(3)	122	-

Spectroelectrochemistry

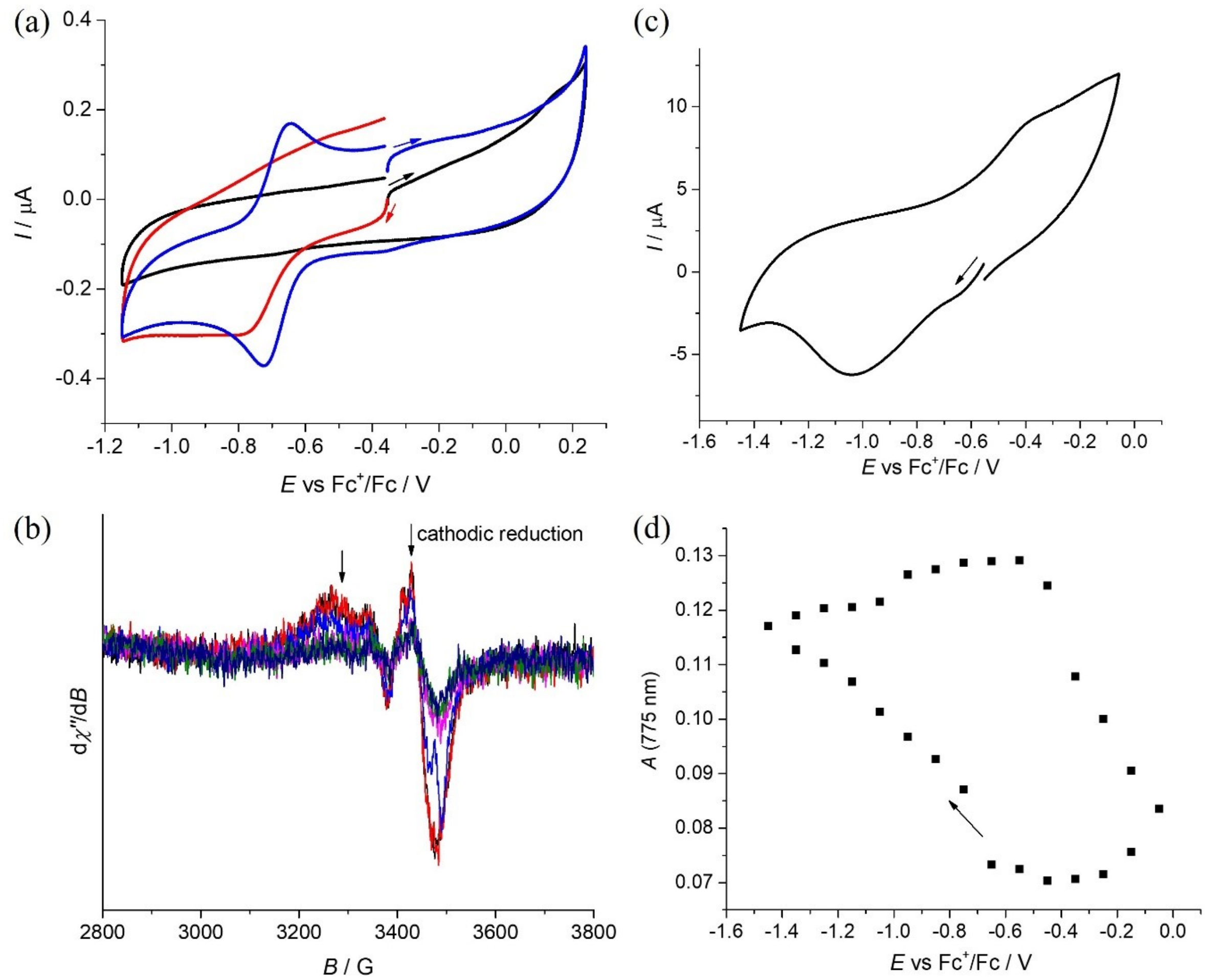

Figure S30. (a) Cyclic voltammograms of 0.5 mM **HL** (black line), **1** (blue line) in DMSO/*n*-Bu_4NPF_6 and of **1** in DMSO:H_2O (1:2)/*n*-Bu_4NPF_6 (red line) at a scan rate of 100 mV s^{-1}(b) Changes in EPR spectra of **2** in DMSO/*n*-Bu_4NPF_6 measured at the first reduction peak using Pt mesh working electrode; (c) In situ cyclic voltammogram for **4** in DMSO/*n*-Bu_4NPF_6 (scan ; rate of 10 mV s^{-1}) and (d) The corresponding changes in absorbance intensity of the optical band at 775 nm as a function of the applied potential during the cyclic voltametric scan.

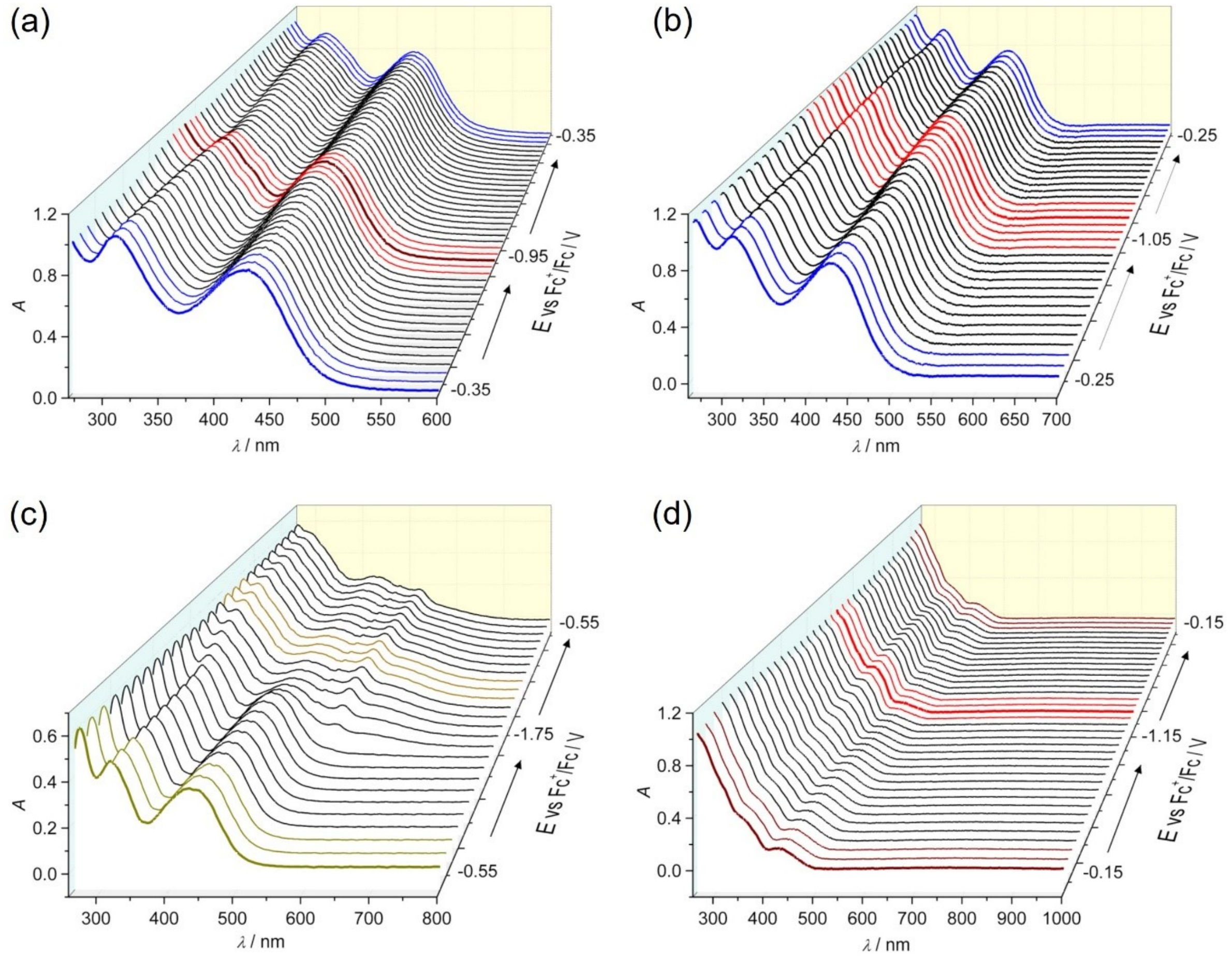

Figure S31. UV–vis spectra measured during cyclic voltammetry of (a) **1**, (b) **2**, (c) **3** and (d) **4** in DMSO/n-Bu_4NPF_6 measured at the first reduction peak (scan rate 5 mV s^{-1}).

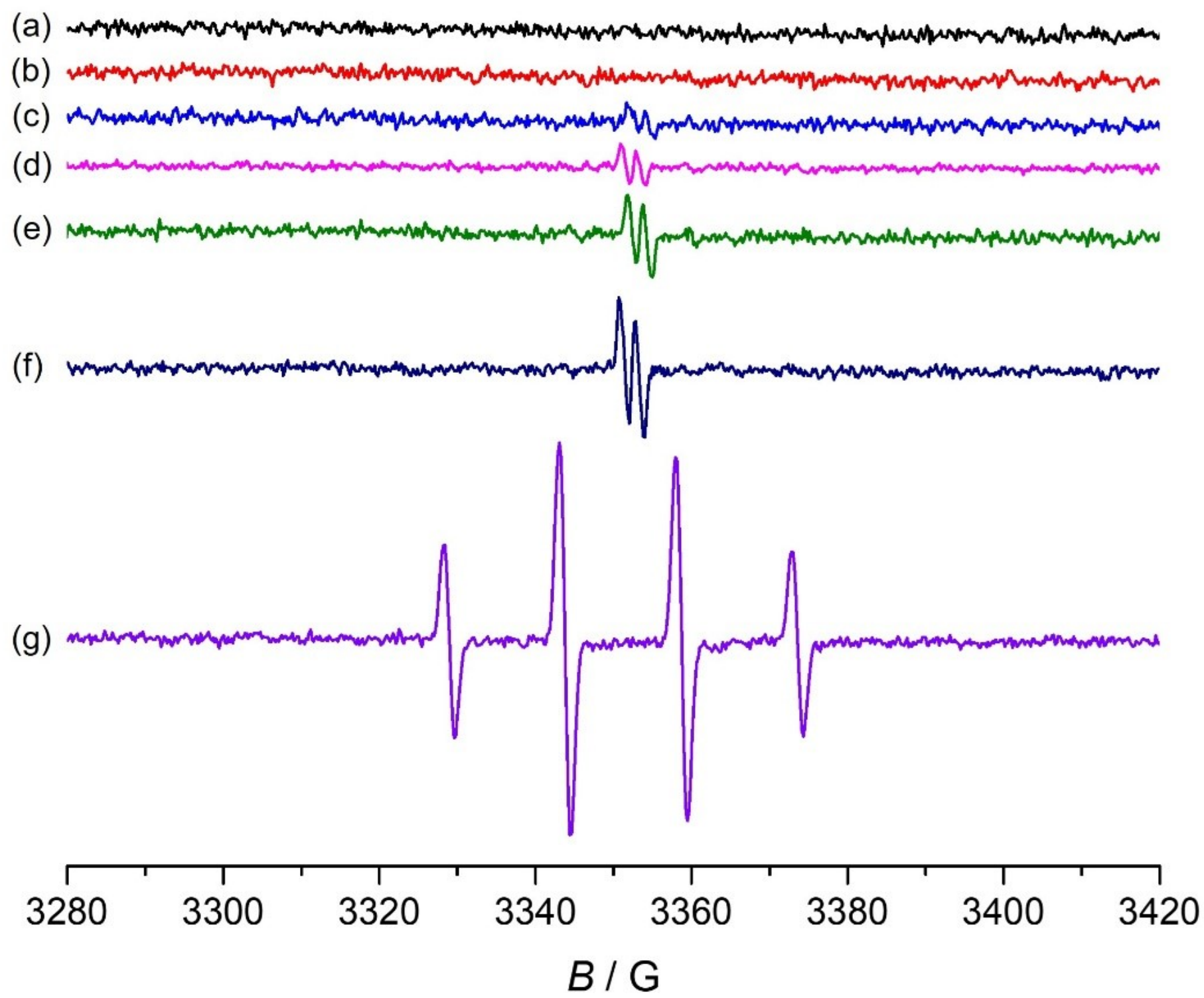

Figure S32. Experimental EPR spectra measured for the systems in air in water solutions: (a) H_2O/DMPO (black trace), (b) H_2O/DMPO/H_2O_2 (red trace), (c) H_2O/DMPO/Asc (blue trace), (d) H_2O/DMPO/Asc/H_2O_2 (magenta trace), (e) HEPES/DMPO/Asc/H_2O_2 (green trace), (f) **2** in H_2O/DMPO/Asc (dark blue trace), and (g) for **2** in H_2O/DMPO/Asc/H_2O_2 (violet trace). Initial concentrations: *c*(**2**) = 0.08 mM, *c*(DMPO) = 0.04 M, *c*(Asc) = 0.4 mM, *c*(H_2O_2) = 0.1 M.

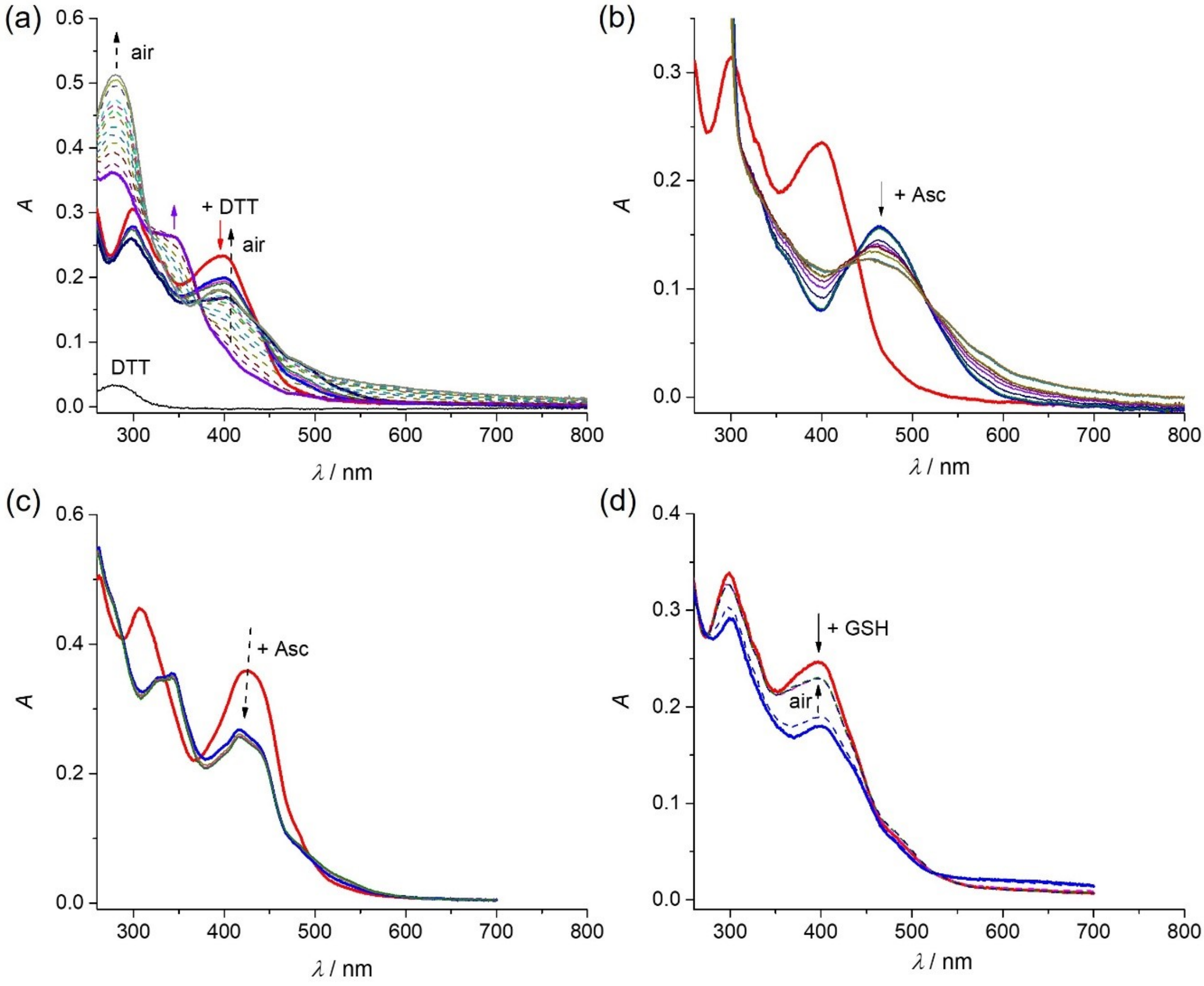

Figure S33. Time-dependent UV−vis absorption spectra of complex **2** in the presence of (a) DTT (1.3% (v/v) DMSO/H_2O mixture), (b) Asc (1.3% (v/v) DMSO/H_2O mixture), (c) Asc (DMSO) and (d) GSH (1.3% (v/v) DMSO/H_2O mixture) before (red lines) and after mixing the solutions (blue lines). Dashed lines: the absorbance changes in air in time.

Solution studies

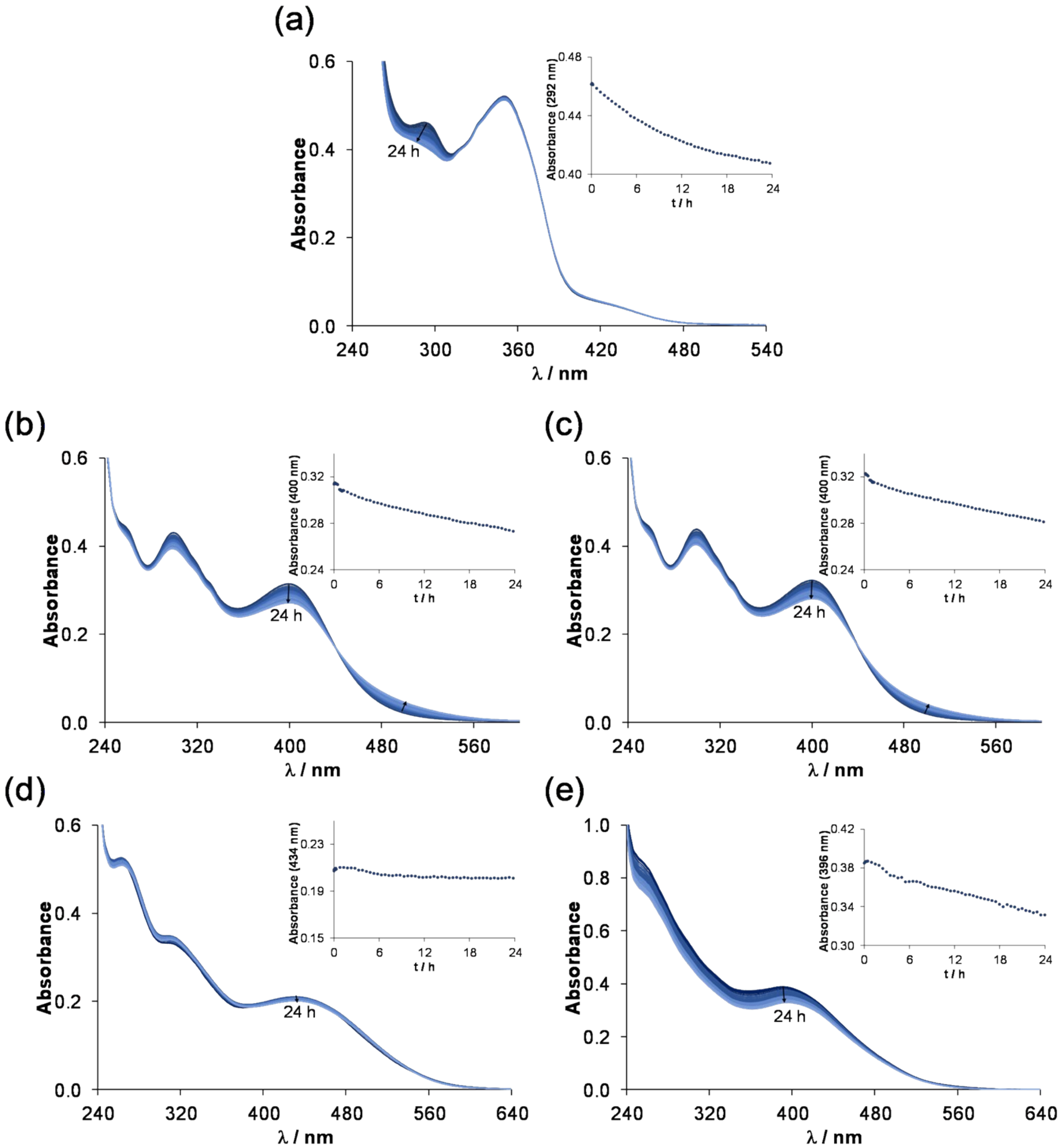

Figure S34. Time-dependence of the UV–vis spectra of (a) **HL** in 50% (*v*/*v*) DMSO/H_2O and (b–e) complexes **1–4** in 2% (*v*/*v*) DMSO/H_2O; {c = 20 μM; T = 25 °C; pH = 7.4 (20 mM HEPES)}.

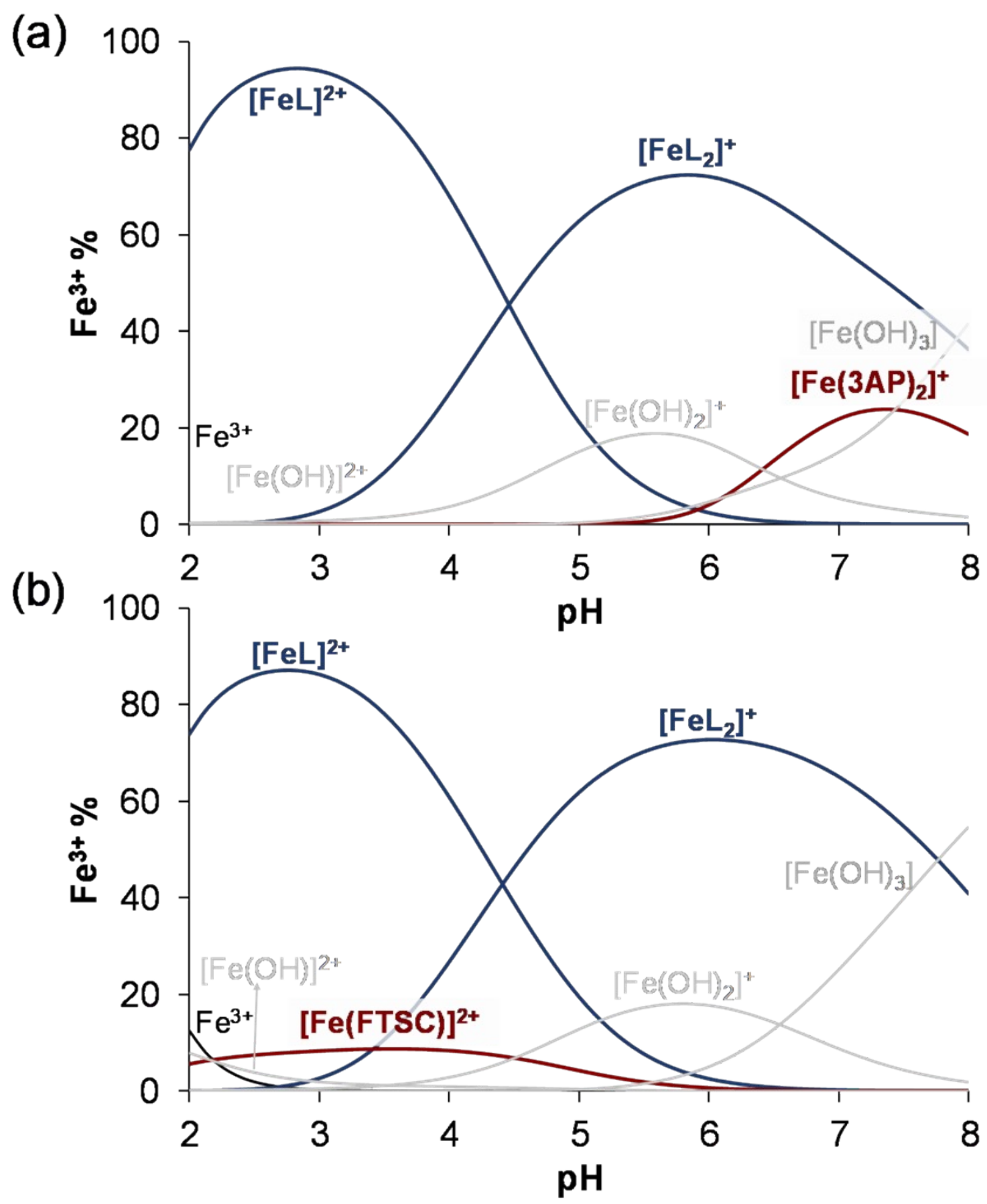

Figure S35. Evaluation of the relative affinity of **HL** and (a) Triapine (3AP) or (b) FTSC towards Fe(III) ions through predominance diagrams; {$c_{\mathbf{HL}}$ = 30 µM; $c_{3AP/FTSC}$ = 30 µM; $c_{Fe(III)}$ = 15 µM}.

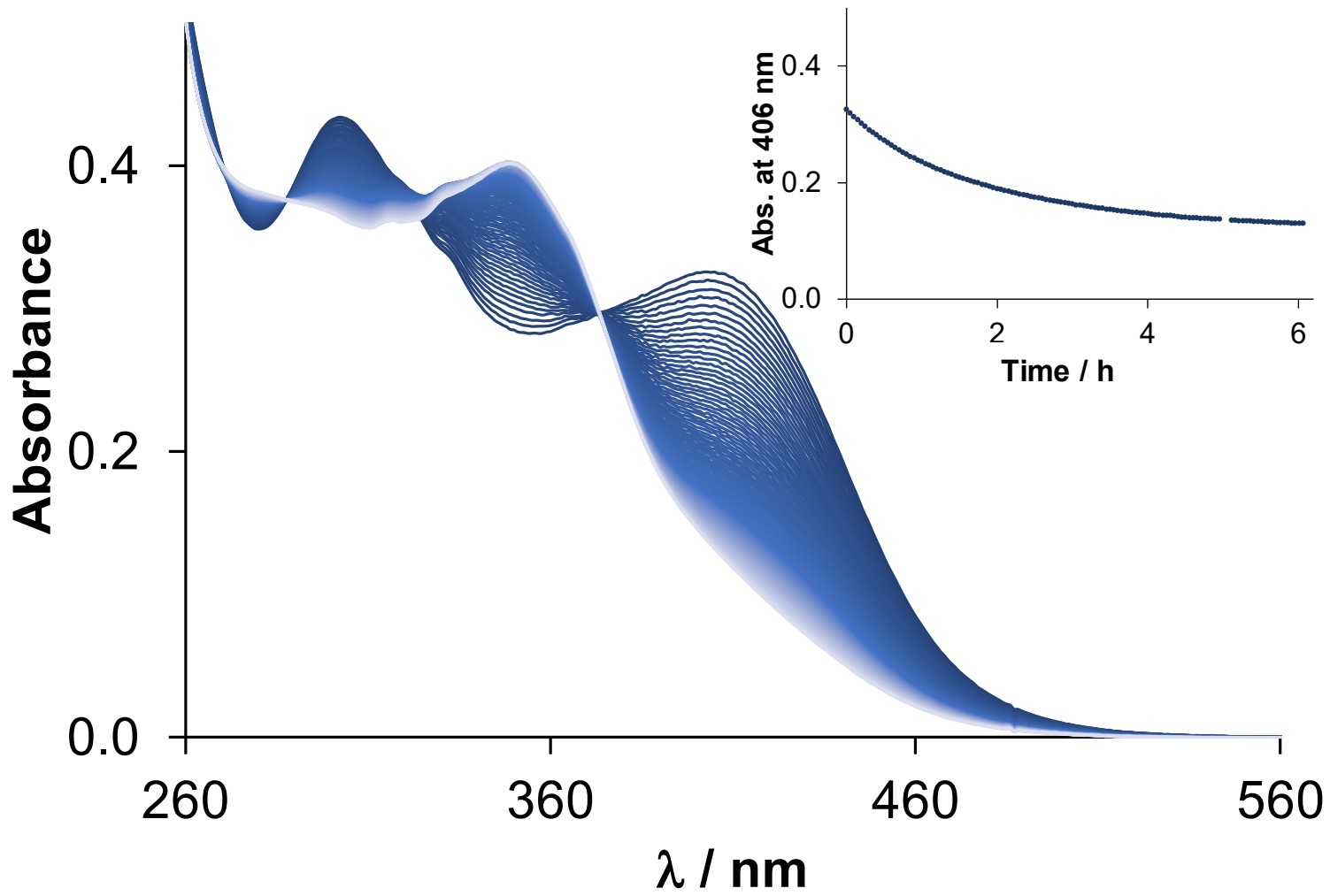

Figure S36. Time-dependent UV–vis absorption spectra of the complex **1** – EDTA (1:1.2) system during the competition reaction. Inserted figure illustrates the absorbance changes at 406 nm as a function of time, indicating that chemical equilibrium was reached within 6 h. (Equilibration was also monitored at an EDTA excess of 18.3-fold, where the required time was found to be 1 h); {$c_{\mathbf{1}}$ = 22 µM; c_{EDTA} = 402 µM; pH = 5.90 (50 mM MES); 30% (v/v) DMSO/H_2O; I = 0.10 M (KCl); l = 1 cm; T = 25.0 °C}.

R2 RNR inhibition

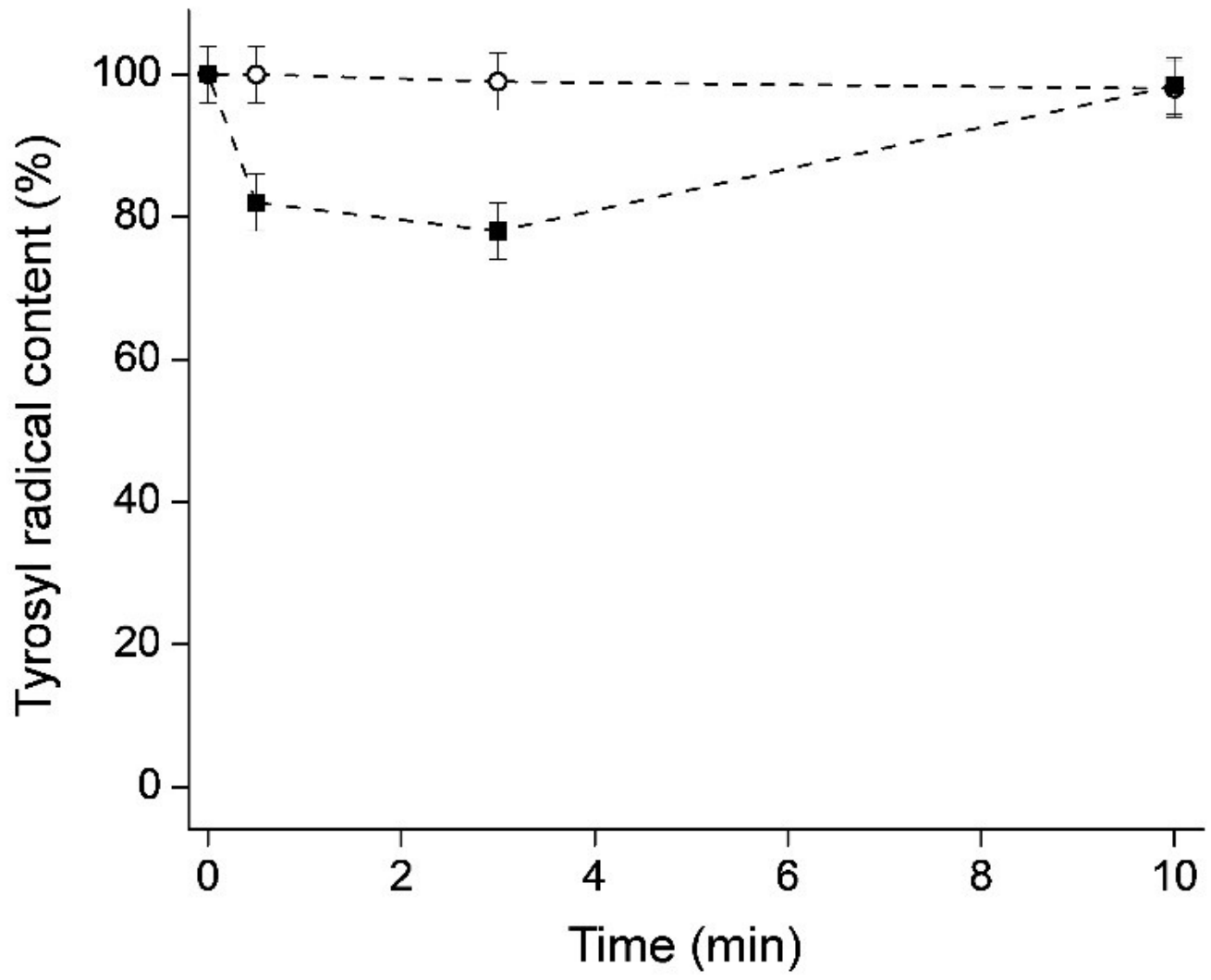

Figure S37. The natural Y$^{\bullet}$ decay in control samples in absence (○) and presence (■) of DTT. The samples contained 20 µM mR2 RNR protein and 2 mM DTT (only ■).

Biological data

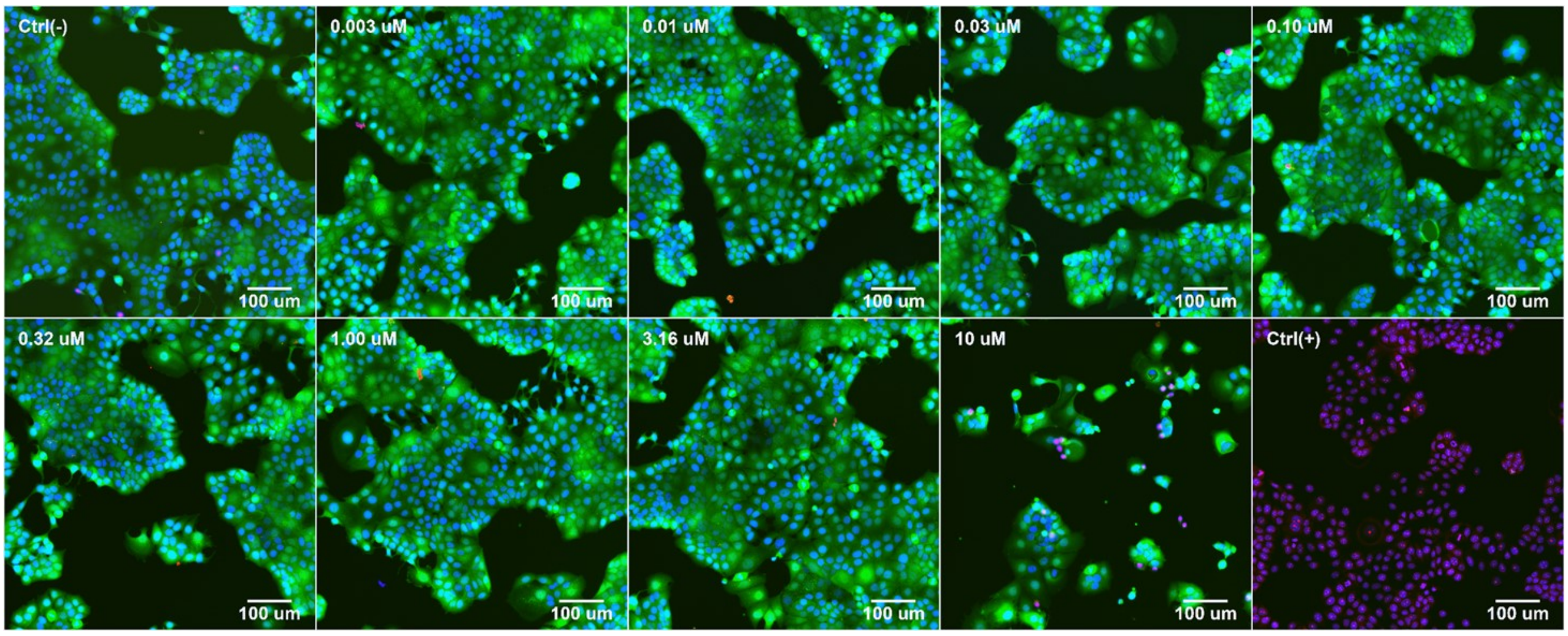

Figure S38. Concentration-dependent effects of **HL** on MCF-7 cell viability assessed by fluorescence live/dead staining. Representative fluorescence overlay images acquired on the ImageXpress® Confocal HT.ai high-content screening system (Molecular Devices) showing MCF-7 cells stained with Hoechst 33342 (nuclei, blue), Calcein-AM (viable cells, green), and propidium iodide (PI; dead cells, magenta) following 48 h of treatment with **HL** across a concentration range of 0.003–10 µM. The untreated negative control [Ctrl(-)] and the Triton X-100-treated positive control [Ctrl(+)] are shown at the left and right ends of the panel, respectively. Scale bars: 100 µm.

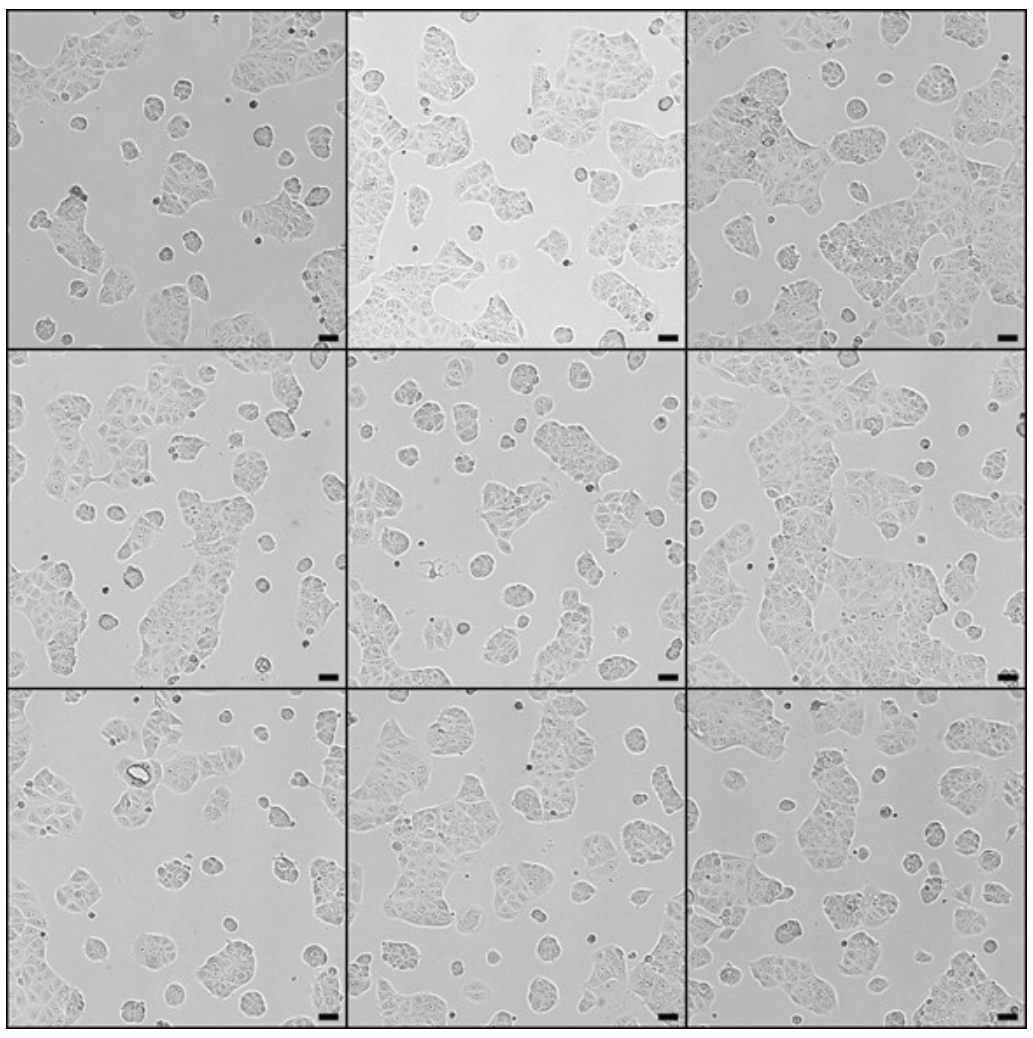

Figure S39. Bright-field morphology of MCF-7 cells following 24 h of treatment with compound **HL** at increasing concentrations. Representative bright-field images acquired on the ImageXpress® Confocal HT.ai high-content screening system (Molecular Devices) showing MCF-7 cell morphology after 24 h of exposure to nocodazole. Images are arranged from left to right and top to bottom in order of increasing concentration: untreated control [Ctrl(-)] (upper left), followed by 0.003, 0.01, 0.03, 0.10, 0.32, 1.00, 3.16, and 10 µM. Scale bars: 50 µm.

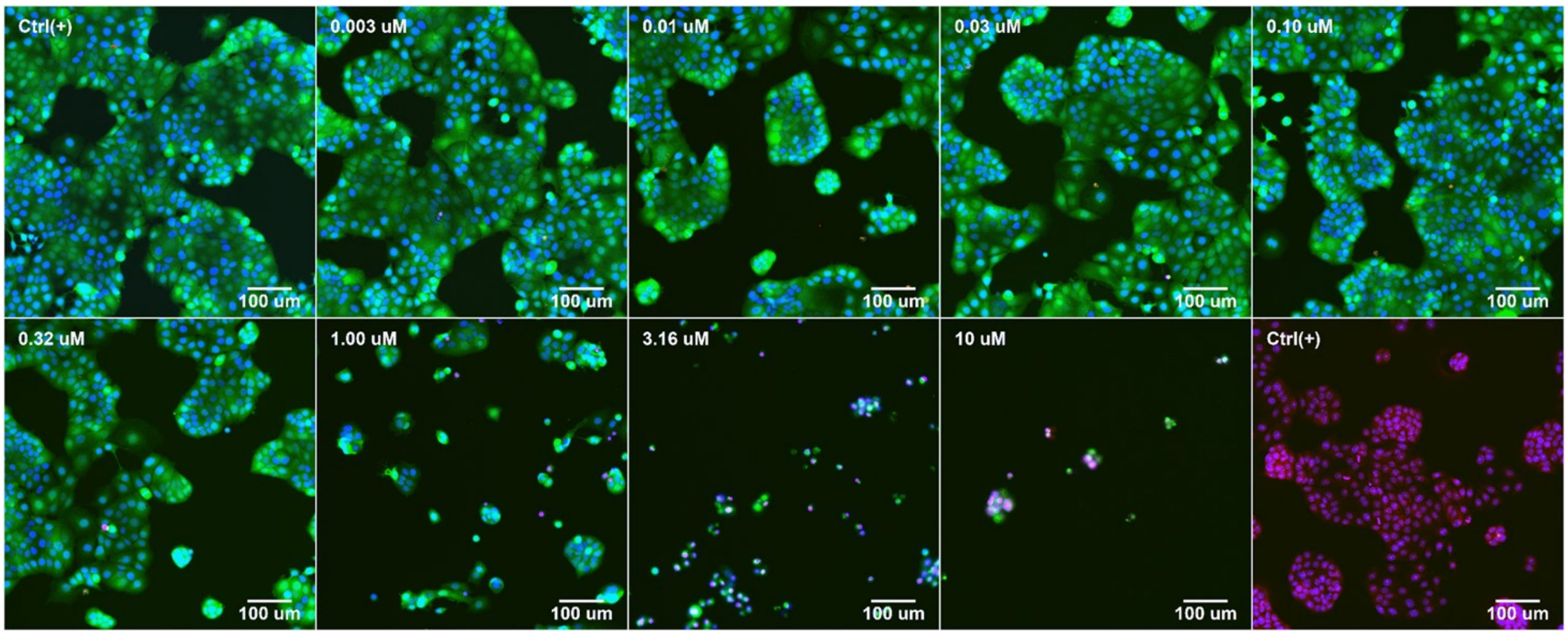

Figure S40. Concentration-dependent effects of compound **1** on MCF-7 cell viability assessed by fluorescence live/dead staining. Representative fluorescence overlay images acquired on the ImageXpress® Confocal HT.ai high-content screening system (Molecular Devices) showing MCF-7 cells stained with Hoechst 33342 (nuclei, blue), Calcein-AM (viable cells, green), and propidium iodide (PI; dead cells, magenta) following 48 h of treatment with **1** across a concentration range of 0.003–10 µM. The untreated negative control [Ctrl(-)] and the Triton X-100-treated positive control [Ctrl(+)] are shown at the left and right ends of the panel, respectively. Scale bars: 100 µm.

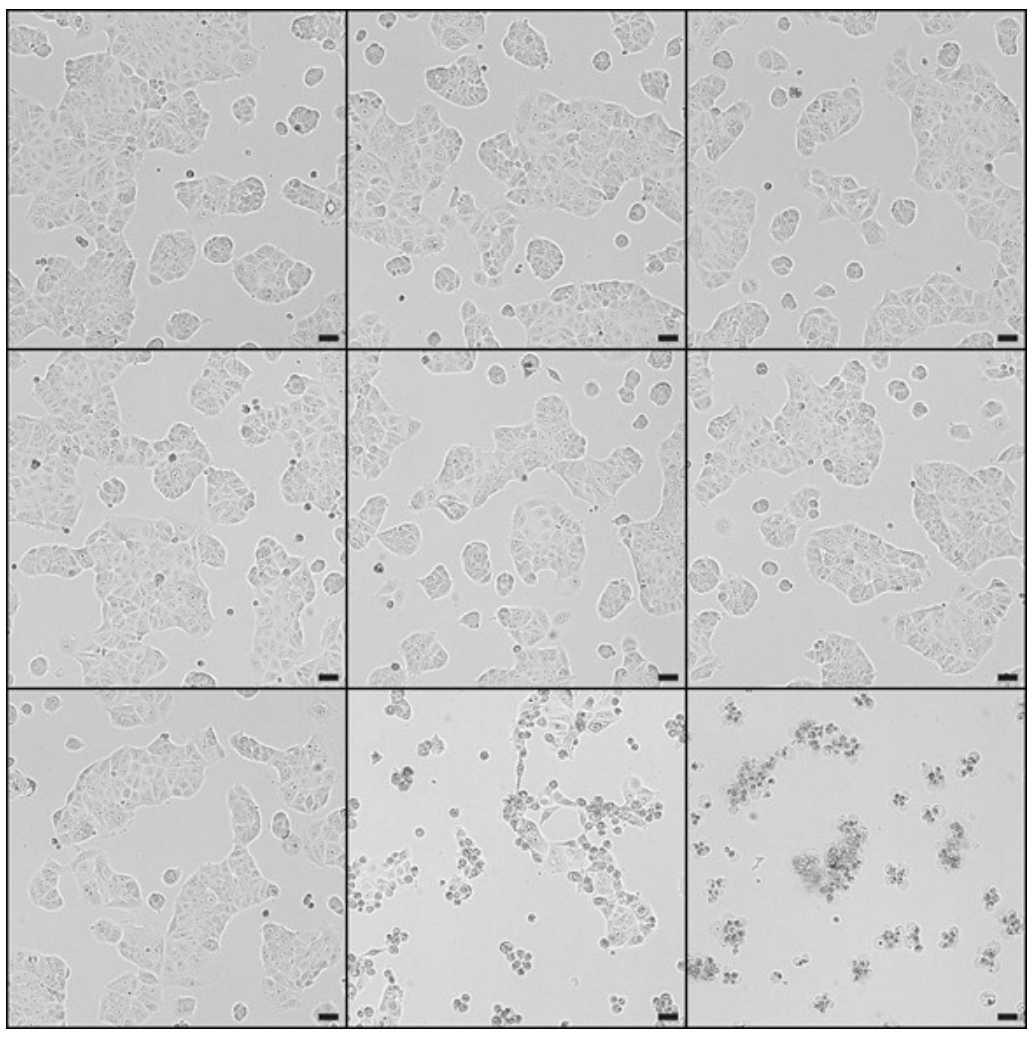

Figure S41. Bright-field morphology of MCF-7 cells following 24 h of treatment with compound **1** at increasing concentrations. Representative bright-field images acquired on the ImageXpress® Confocal HT.ai high-content screening system (Molecular Devices) showing MCF-7 cell morphology after 24 h of exposure to compound **1**. Images are arranged from left to right and top to bottom in order of increasing concentration: untreated control [Ctrl(-)] (upper left), followed by 0.003, 0.01, 0.03, 0.10, 0.32, 1.00, 3.16, and 10 µM. Scale bars: 50 µm.

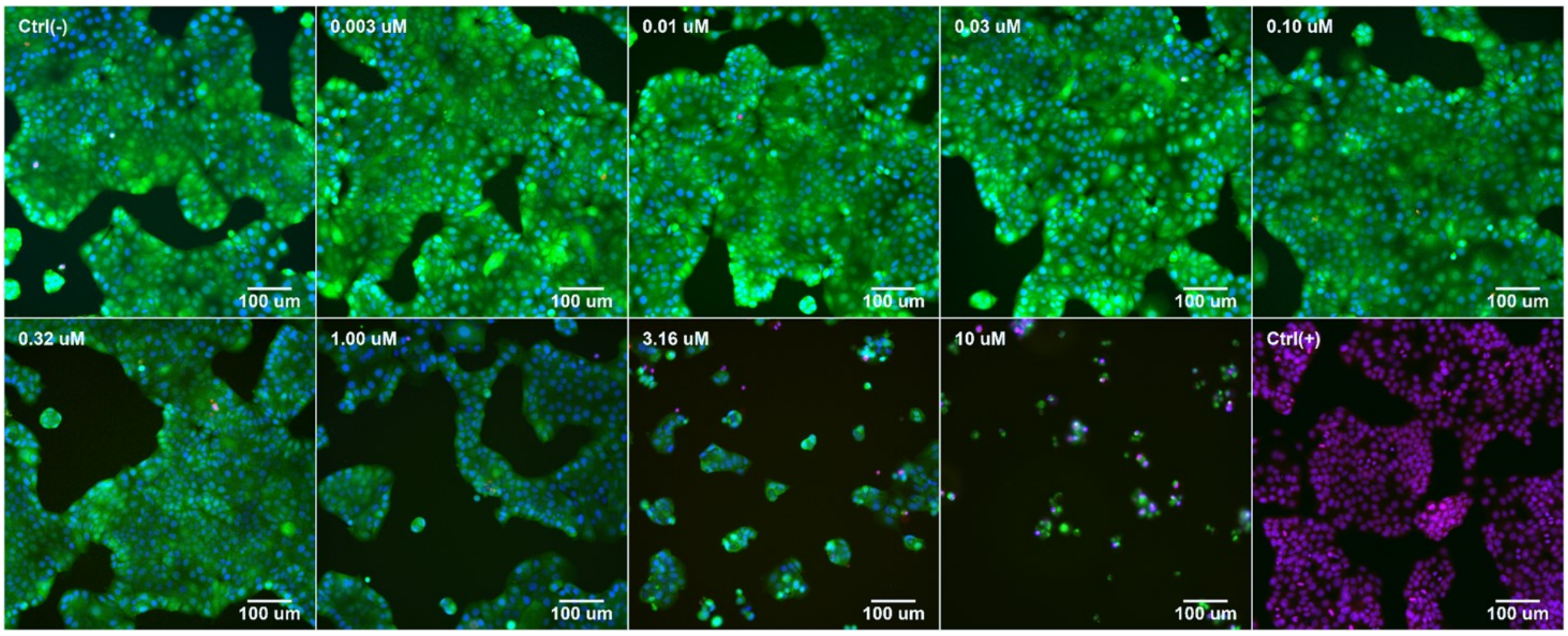

Figure S42. Concentration-dependent effects of compound **2** on MCF-7 cell viability assessed by fluorescence live/dead staining. Representative fluorescence overlay images acquired on the ImageXpress® Confocal HT.ai high-content screening system (Molecular Devices) showing MCF-7 cells stained with Hoechst 33342 (nuclei, blue), Calcein-AM (viable cells, green), and propidium iodide (PI; dead cells, magenta) following 48 h of treatment with **2** across a concentration range of 0.003–10 µM. The untreated negative control [Ctrl(-)] and the Triton X-100-treated positive control [Ctrl(+)] are shown at the left and right ends of the panel, respectively. Scale bars: 100 µm.

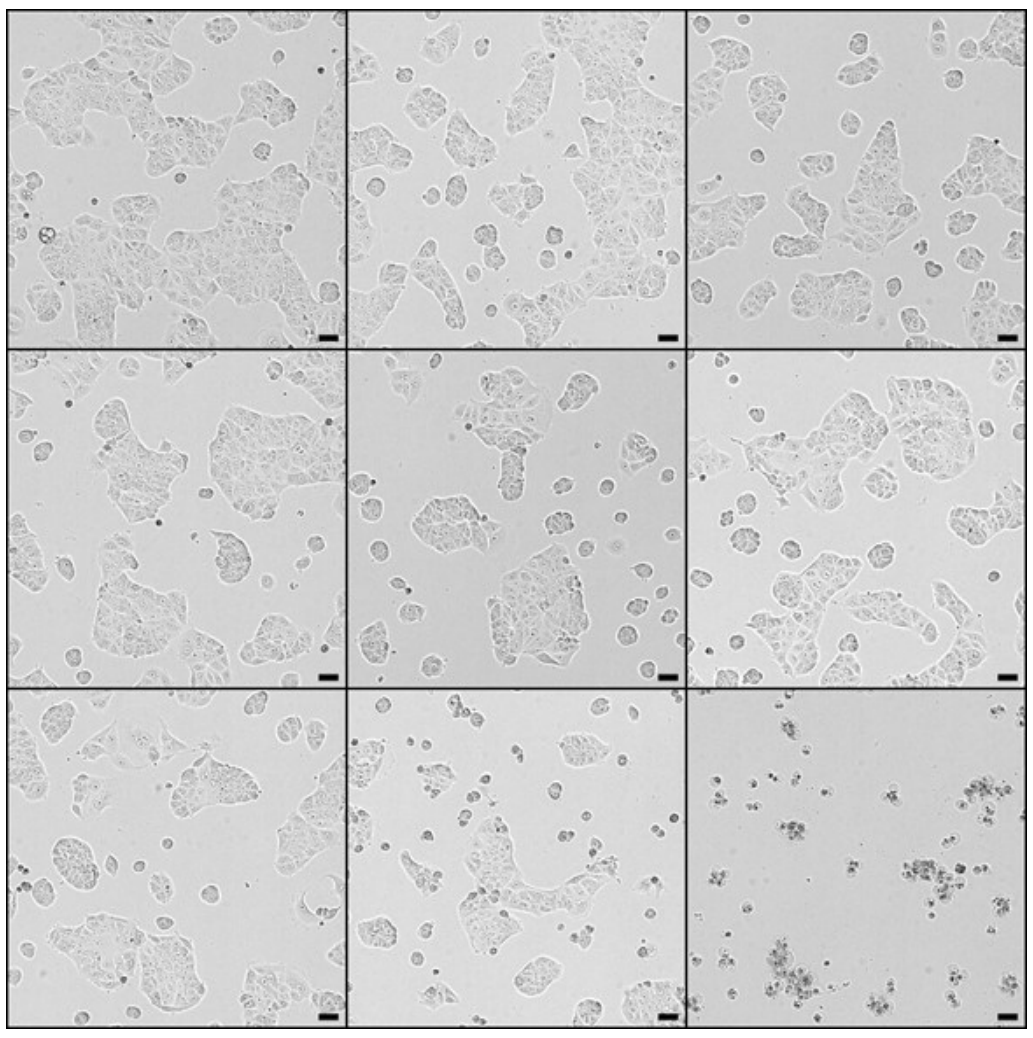

Figure S43. Bright-field morphology of MCF-7 cells following 24 h of treatment with compound **2** at increasing concentrations. Representative bright-field images acquired on the ImageXpress® Confocal HT.ai high-content screening system (Molecular Devices) showing MCF-7 cell morphology after 24 h of exposure to compound **2**. Images are arranged from left to right and top to bottom in order of increasing concentration: untreated control [Ctrl(-)] (upper left), followed by 0.003, 0.01, 0.03, 0.10, 0.32, 1.00, 3.16, and 10 µM. Scale bars: 50 µm.

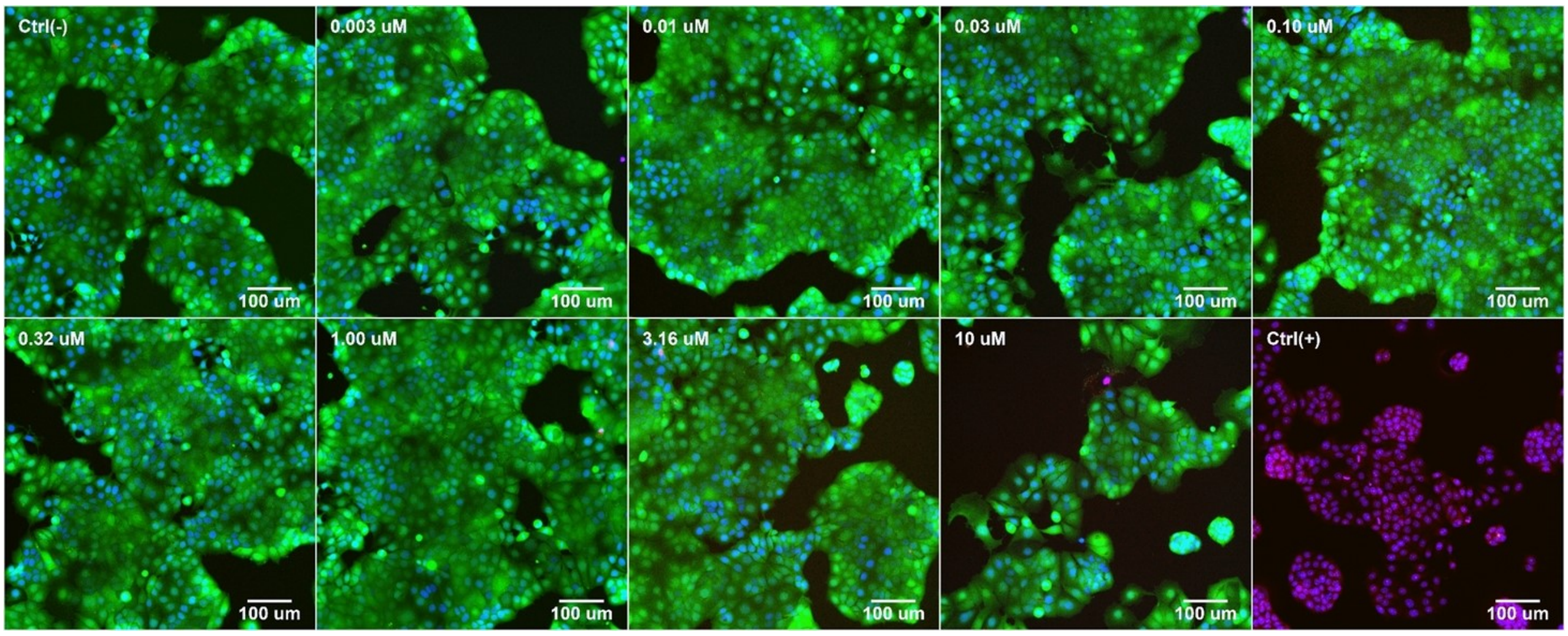

Figure S44. Concentration-dependent effects of compound **3** on MCF-7 cell viability assessed by fluorescence live/dead staining. Representative fluorescence overlay images acquired on the ImageXpress® Confocal HT.ai high-content screening system (Molecular Devices) showing MCF-7 cells stained with Hoechst 33342 (nuclei, blue), Calcein-AM (viable cells, green), and propidium iodide (PI; dead cells, magenta) following 48 h of treatment with compound (3) across a concentration range of 0.003–10 µM. The untreated negative control [Ctrl(-)] and the Triton X-100-treated positive control [Ctrl(+)] are shown at the left and right ends of the panel, respectively. Scale bars: 100 µm.

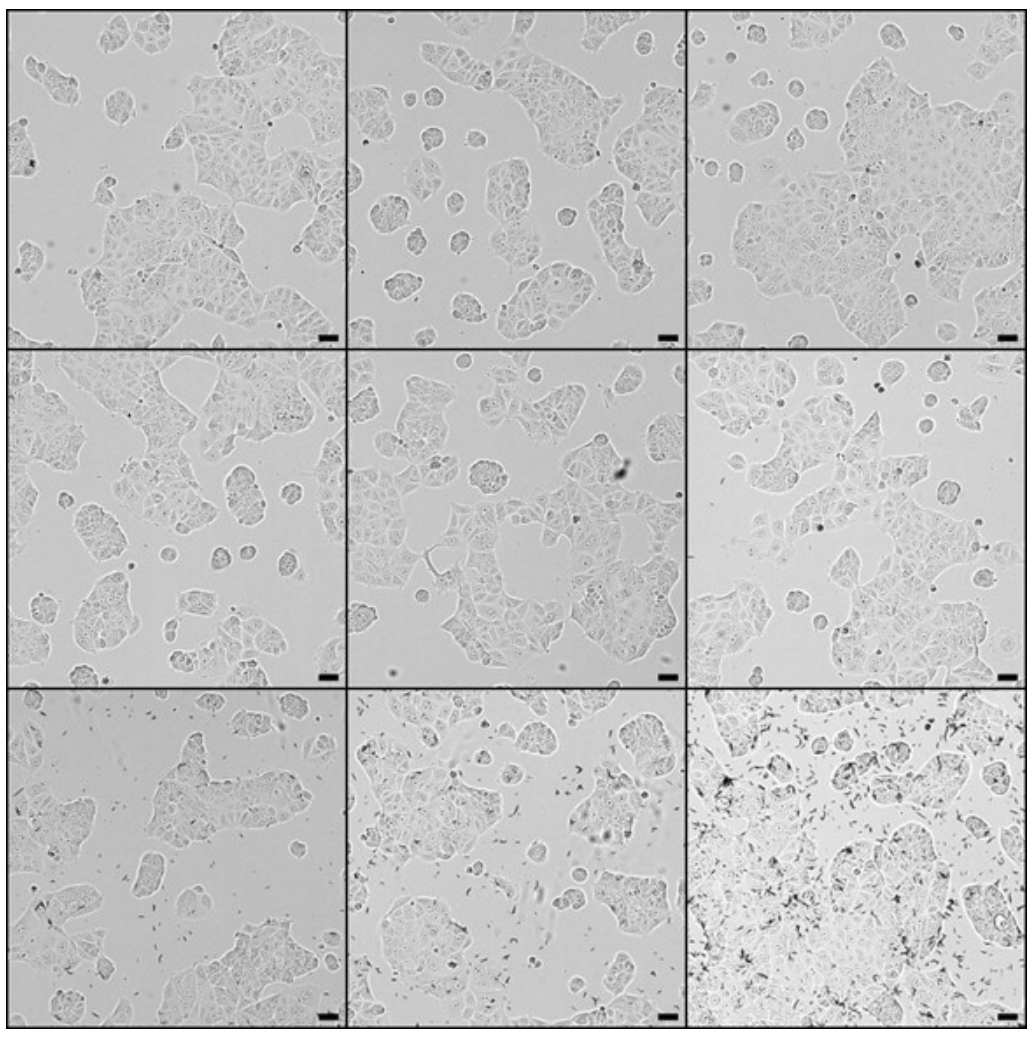

Figure S45. Bright-field morphology of MCF-7 cells following 24 h of treatment with compound **3** at increasing concentrations. Representative bright-field images acquired on the ImageXpress® Confocal HT.ai high-content screening system (Molecular Devices) showing MCF-7 cell morphology after 24 h of exposure to **3**. Images are arranged from left to right and top to bottom in order of increasing concentration: untreated control [Ctrl(-)] (upper left), followed by 0.003, 0.01, 0.03, 0.10, 0.32, 1.00, 3.16, and 10 µM. Scale bars: 50 µm.

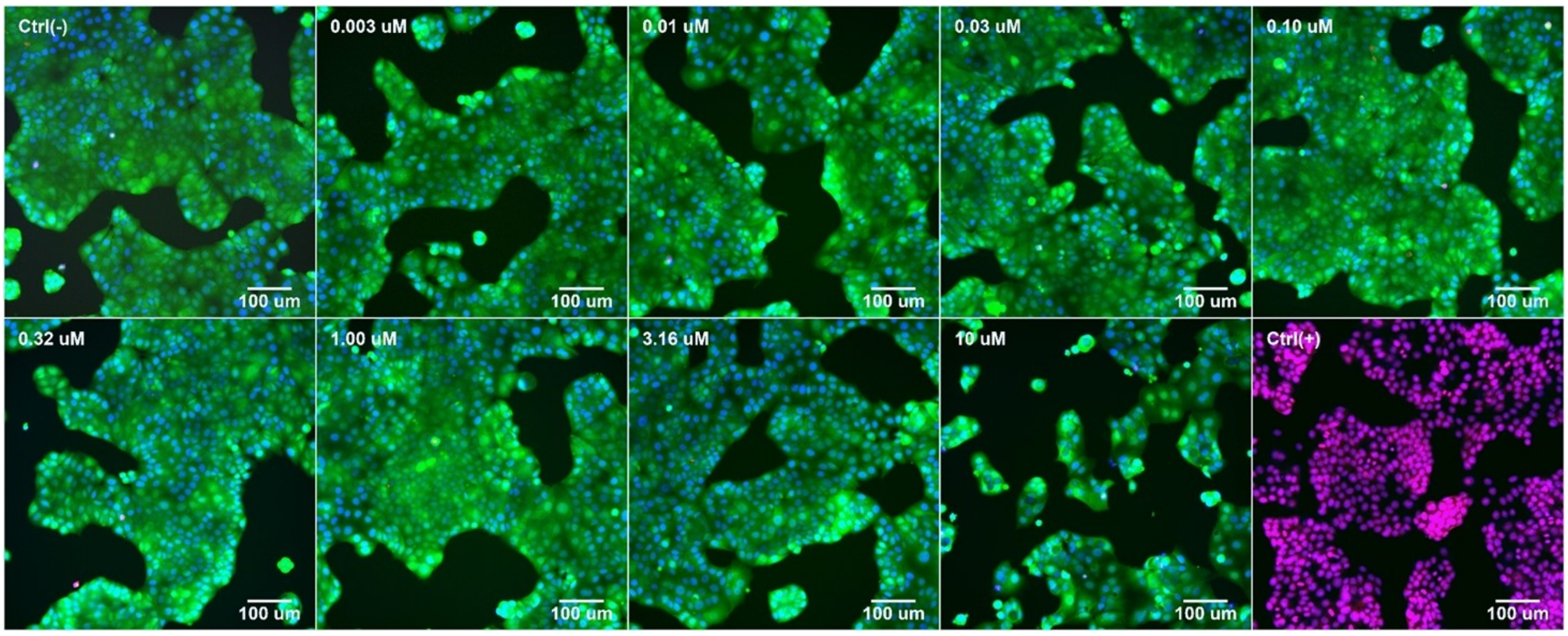

Figure S46. Concentration-dependent effects of compound **4** on MCF-7 cell viability assessed by fluorescence live/dead staining. Representative fluorescence overlay images acquired on the ImageXpress® Confocal HT.ai high-content screening system (Molecular Devices) showing MCF-7 cells stained with Hoechst 33342 (nuclei, blue), Calcein-AM (viable cells, green), and propidium iodide (PI; dead cells, magenta) following 48 h of treatment with **4** across a concentration range of 0.003–10 µM. The untreated negative control [Ctrl(-)] and the Triton X-100-treated positive control [Ctrl(+)] are shown at the left and right ends of the panel, respectively. Scale bars: 100 µm.

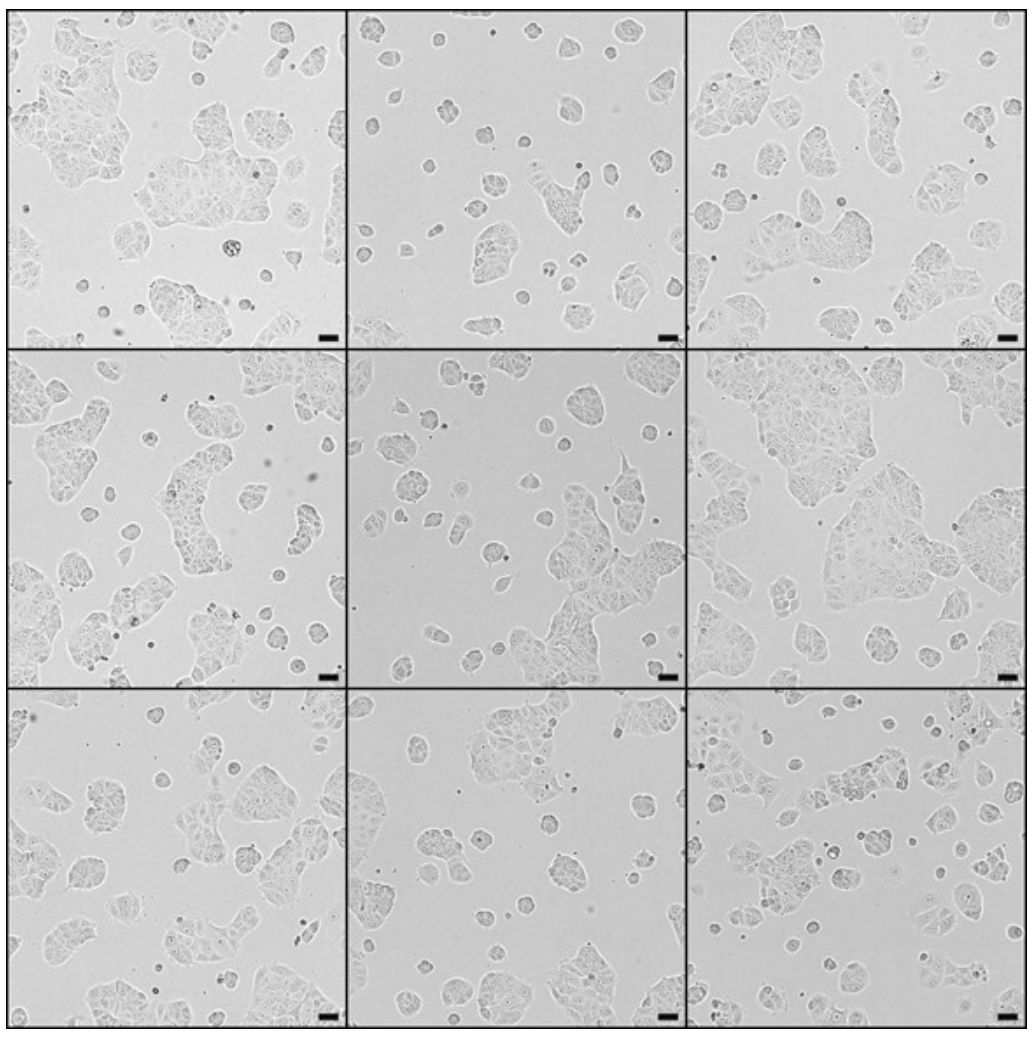

Figure S47. Bright-field morphology of MCF-7 cells following 24 h of treatment with compound **4** at increasing concentrations. Representative bright-field images acquired on the ImageXpress® Confocal HT.ai high-content screening system (Molecular Devices) showing MCF-7 cell morphology after 24 h of exposure to **4**. Images are arranged from left to right and top to bottom in order of increasing concentration: untreated control [Ctrl(-)] (upper left), followed by 0.003, 0.01, 0.03, 0.10, 0.32, 1.00, 3.16, and 10 µM. Scale bars: 50 µm.

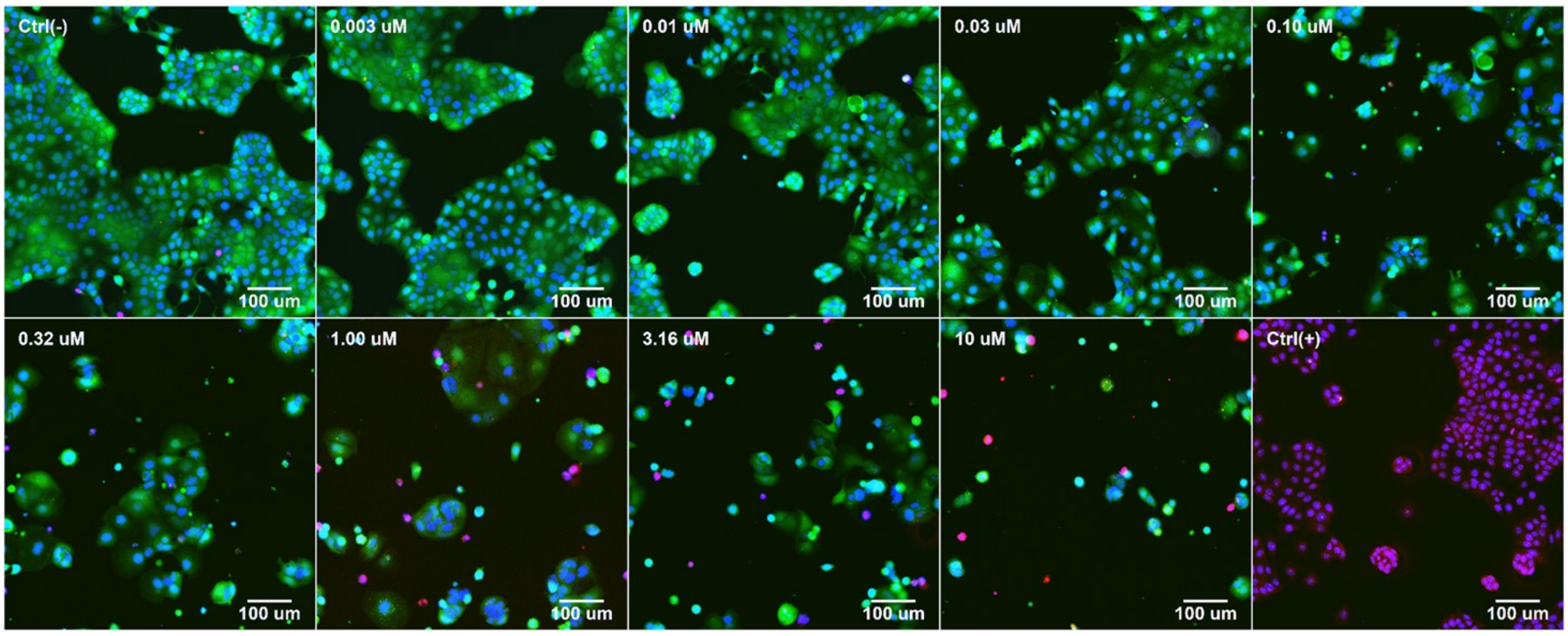

Figure S48. Concentration-dependent effects of nocodazole on MCF-7 cell viability assessed by fluorescence live/dead staining. Representative fluorescence overlay images acquired on the ImageXpress® Confocal HT.ai high-content screening system (Molecular Devices) showing MCF-7 cells stained with Hoechst 33342 (nuclei, blue), Calcein-AM (viable cells, green), and propidium iodide (PI; dead cells, magenta) following 48 h of treatment with nocodazole across a concentration range of 0.003–10 μM. The untreated negative control [Ctrl(-)] and the Triton X-100-treated positive control [Ctrl(+)] are shown at the left and right ends of the panel, respectively. Scale bars: 100 μm.

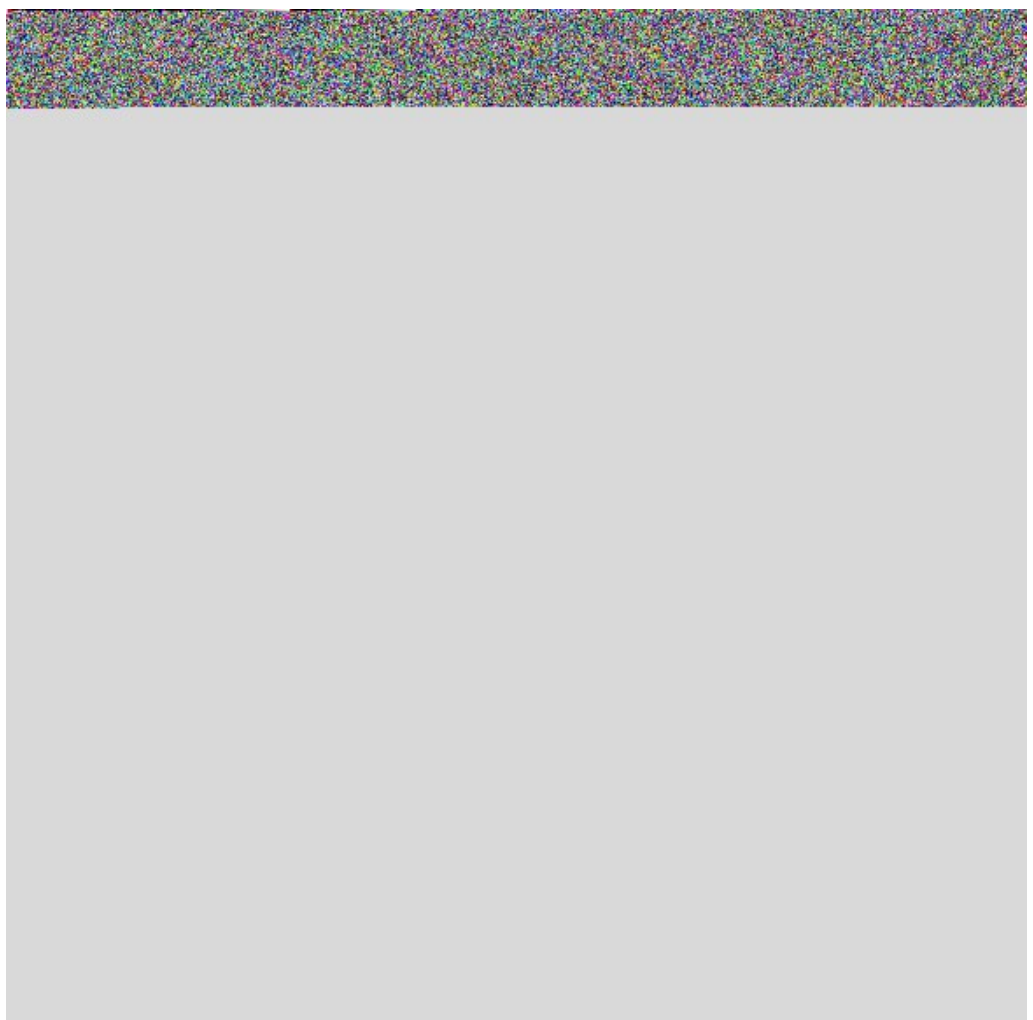

Figure S49. Bright-field morphology of MCF-7 cells following 24 h of treatment with nocodazole at increasing concentrations. Representative bright-field images acquired on the ImageXpress® Confocal HT.ai high-content screening system (Molecular Devices) showing MCF-7 cell morphology after 24 h of exposure to nocodazole. Images are arranged from left to right and top to bottom in order of increasing concentration: untreated control [Ctrl(-)] (upper left), followed by 0.003, 0.01, 0.03, 0.10, 0.32, 1.00, 3.16, and 10 μM. Scale bars: 50 μm.

Materials

All starting materials for the synthesis of **HL** and complexes **1–4** were purchased from commercial suppliers and used without further purification. The human breast adenocarcinoma cell line MCF-7 was obtained from Cytion (CLS Cell Lines Service GmbH, Eppelheim, Germany). Alpha-MEM Eagle (αMEM) culture medium and fetal bovine serum (FBS) were sourced from PAN Biotech (Aidenbach, Germany). Antibiotic-antimycotic solution was purchased from Capricorn Scientific (Ebsdorfergrund, Germany). TrypLE Select 10× was acquired from Gibco (Paisley, UK). The CellTiter-Glo® Luminescent Cell Viability Assay kit was obtained from Promega (Madison, WI, USA). White opaque 96-well microplates were supplied by PerkinElmer (Germany). Hoechst 33342, the primary antibodies anti-alpha Tubulin mouse monoclonal antibody (236-10501) and anti-Phospho-Histone H3 (Ser10) recombinant rabbit monoclonal antibody (9H12L10), as well as the secondary antibodies goat anti-mouse IgG (H+L) conjugated with DyLight™ 488 and donkey anti-rabbit IgG (H+L) cross-adsorbed secondary antibody conjugated with DyLight™ 755, were all procured from Invitrogen (USA). Calcein-AM was purchased from Biotium (Fremont, CA, USA). Propidium iodide, nocodazole, Triton X-100, and dimethyl sulfoxide (DMSO) were obtained from Sigma-Aldrich (St. Louis, MO, USA).

Synthesis of the ligand and metal complexes

Synthesis of HL

***N*1-(3,4,5-trimethoxyphenyl)-*N*2-dimethylthiourea**. A mixture of 3,4,5-trimethoxyaniline (5.49 g, 30 mmol) and tetramethylthiuram disulfide (7.2 g, 30 mmol) in DMF (50 mL) was heated at 110 °C for 2 h and allowed to cool to room temperature. The precipitate was separated by filtration, while the filtrate was poured in ice-cold water (50 mL). After cooling to room temperature a white-gray precipitate was formed. This was separated by filtration, washed with cold water and dried in vacuum for 2 h. Yield: 6.1 g (74%). Calcd for $C_{12}H_{18}N_2O_3S{\cdot}0.019S_8$ (M_r 275.22), %: C, 52.37; H, 6.59; N, 10.18; S, 13.42. Found, %: C, 52.30; H, 6.58; N, 10.31; S, 13.09.

***N*-(3,4,5-trimethoxyphenyl)isothiocyanate**. Dry hydrogen chloride was bubbled through a solution of *N*1-(3,4,5-trimethoxyphenyl)-*N*2-dimethylthiourea (6.1 g, 22.2 mmol) in dry dioxane (100 mL) at 100 °C for 1 h. The suspension was poured into cold water (70 mL). After cooling the reaction mixture to room temperature, the white precipitate formed was filtered, washed with cold water (20 mL) and dried in vacuum for 2 h. Yield: 4.05 g (78%). Calcd for

$C_{10}H_{11}NO_3S·0.4H_2O$ (M_r 232.47), %: C, 51.67; H, 5.12; N, 6.03; S, 13.79. Found, %: C, 51.18; H, 4.82; N, 6.38; S, 14.21.

***N*4-(3,4,5-trimethoxyphenyl)thiosemicarbazide**. To a clear solution of *N*-(3,4,5-trimethoxyphenyl)isothiocyanate (4.05 g, 17.4 mmol) in ethanol (30 mL) upon gentle stirring hydrazine hydrate (4 mL) was added dropwise. The white precipitate formed after ca. 15 min was filtered, washed with water (10 mL), ethanol (10 mL) and dried in vaccuo. Yield: 3.1 g (69%). Calcd for $C_{10}H_{15}N_3O_3S$ (M_r 257.31), %: C, 46.68; H, 5.88; N, 16.33; S, 12.46. Found, %: C, 46.28; H, 5.82; N, 16.04; S, 14.38.

Quinoline-8-carboxaldehyde *N*4-(3,4,5-trimethoxyphenyl)thiosemicarbazide (HL). A mixture of quinoline-8-carboxaldehyde (1.57 g, 10 mmol) and *N*4-(3,4,5-trimethoxyphenyl)thiosemicarbazide (2.57 g, 10 mmol) in ethanol (30 mL) was refluxed for 2 h. The solution was allowed to cool to room temperature and the orange crystals formed were separated by filtration, washed with cold ethanol (2 × 10 mL) and dried in vacuum for 2 h. Yield: 3.85 g (97%). Calcd for $C_{20}H_{20}N_4O_3S$ (M_r 396.46), %: C, 60.59; H, 5.08; N, 14.13; S, 8.09. Found, %; C, 60.69; H, 5.08; N, 14.06, S, 7.81. Positive ion HR ESI MS: *m*/*z* 397.1316 attributed to $[M+H^+]^+$; calcd *m*/*z* 397.1329; *m*/*z* 419.1135 attributed to $[M+Na^+]^+$; calcd *m*/*z* 419.1154; *m*/*z* 815.2379 attributed to $[2M+Na^+]^+$; calcd *m*/*z* 815.2410; *m*/*z* 1211.3632 attributed to $[3M+Na^+]^+$; calcd *m*/*z* 1211.3666. ^{1}H NMR (400 MHz, DMSO-d_6, δ, ppm): ^{1}H NMR (400 MHz, DMSO-d_6, δ, ppm): 12.07 (s, 1H, NH-3), 10.13 (s, 1H, NH-4), 9.46 (s, 1H, H10), 9.00 (dd, J = 1.7 Hz, 4.1 Hz, 1H, H1), 8.80 (d, J = 6.5 Hz, 1H, H7), 8.45 (dd, J = 1.5 Hz, 8.3 Hz, 1H, H3), 8.06 (d, J = 7.1 Hz, 1H, H5), 7.71 (t, J = 7.7 Hz, 1H, H6), 7.63 (dd, J = 4.1 Hz, 8.3 Hz, 1H, H2), 7.06 (s, 2H, H13, H17), 3.78 (s, 6H, H18,20), 3.68 (s, 3H, H19). ^{13}C NMR (100 MHz, DMSO-d_6, δ, ppm): 175.7 (C11), 152.2 (C14,16), 150.4 (C1), 145.5 (C9), 139.8 (C10), 136.6 (C3), 135.0 (C12), 134.8 (C15), 130.9 (C8), 130.0 (C5), 128.0 (C4), 126.6 (C7), 126.4 (C6), 121.8 (C2), 103.4 (C13,17), 60.1 (C19), 55.9 (C18,20).

Synthesis of metal complexes

[Cu(HL)(Cl_2CHCOO)](Cl_2CHCOO) (1′). To a mixture of **HL** (0.40 g, 1.0 mmol) in ethanol (30 mL) and 1 M dichloroacetic acid (10 mL) was added dropwise a solution obtained from $Cu_2(OH)_2CO_3$ (0.11 g, 0.5 mmol) and 1 M dichloroacetic acid (10 mL). The reaction mixture was stirred for 1 h. The green microcrystals formed were separated by filtration, washed with water (2 × 10 mL) and dried under vacuum for 3 h. Yield: 0.66 g (92%). Calcd for

$C_{24}H_{22}Cl_4CuN_4O_7S$ (M_r 715.88), %: C, 40.27; H, 3.10; N, 7.83; S, 4.48. Found, %; C, 40.22; H, 3.35; N, 7.78; S, 4.53.

[Cu(L)(Cl_2CHCOO)]·DMSO·H_2O (1·DMSO·H_2O). Re-crystallization of **[Cu(HL)(Cl_2CHCOO)](Cl_2CHCOO)** (100 mg) in DMSO (1.0 mL) produced black-brown crystals of X-ray diffraction quality, which were filtered and washed with diethyl ether (3 × 15 mL). Yield: 75 mg (16%). Calcd for $C_{24}H_{28}Cl_2CuN_4O_7S_2$ (M_r 683.09), %: C, 42.20; H, 4.13; N, 8.20; S, 9.39. Found, %; C, 42.21; H, 3.95; N, 8.20; S, 9.18. Positive ion HR ESI MS: *m*/*z* 458.0470 attributed to $[Cu^{II}L]^+$ (calcd *m*/*z* 458.0574); *m*/*z* 1045.0272 attributed to $[(Cu^{II}L)_2CHCl_2COO^-]^+$ (calcd *m*/*z* 1045.0501); *m*/*z* 1632.0078 attributed to $[(Cu^{II}L)_3(CHCl_2COO^-)_2]^+$ (calcd *m*/*z* 1632.0429).

[Cu(L)Cl]·1.5DMSO·0.75H_2O (2·1.5DMSO·0.75H_2O). To **HL** (0.40 g, 1.0 mmol) in ethanol (30 mL) was added 1 M HCl (20 mL) in one portion and then dropwise a solution obtained from $Cu_2(OH)_2CO_3$ (0.11 g, 0.5 mmol) and 1 M HCl (10 mL). The reaction mixture stirred at room temperature for 1 h produced green microcrystalls, which were separated by filtration, washed with water (2 × 10 mL) and dried under vacuum for 3 h. The product (100 mg) was re-crystallized from DMSO (1 mL) to give green crystals of X-ray diffraction quality, which were separated by filtration and washed with diethyl ether (3 × 15 mL). Yield: 48 mg (58%). Calcd for $C_{23}H_{30}ClCuN_4O_{5.5}S_{2.5}$ (M_r 629.67), %: C, 43.87; H, 4.80; N, 8.90; S, 12.73. Found, %; C, 43.72; H, 4.28; N, 8.67; S, 12.58. Positive ion ESI MS: *m*/*z* 458.10 attributed to $[Cu^{II}L]^+$; calcd *m*/*z* 458.06.

[Pd(L)Cl]·H_2O (3·H_2O). To a solution of **HL** (0.40 g, 1.0 mmol) in DMF (10 mL) was added dropwise a solution of $[PdCl_2(MeCN)_2]$ (0.26 g, 1.0 mmol) in MeCN (15 mL). The reaction mixture was stirred at 50 °C for 1 h. The precipitate formed after 24 h was separated by filtration, washed with cold ethanol (2 × 5 mL) and diethyl ether (2 × 10 mL). Yield: 0.37 g (70%). Calcd for $C_{20}H_{21}ClN_4O_4PdS$ (M_r 555.34), %: C, 43.25; H, 3.81; N, 10.09; S, 5.77. Found, %; C, 43.25; H, 3.86; N, 10.01; S, 5.56. Positive ion ESI MS: *m*/*z* 542.09 attributed to $[Pd^{II}L(CH_3CN)]^+$; calcd *m*/*z* 542.05; *m*/*z* 1038.99 attributed to $[(Pd^{II}L)_2Cl^-]^+$; calcd *m*/*z* 1039.02. ^{1}H NMR (400 MHz, DMSO-*d*$_6$, δ, ppm): 10.14 (dd, *J* = 1.6 Hz, 5.4 Hz, 1H, H3), 9.82 (s, 1H, NH-4), 8.97 (dd, *J* = 1.5 Hz, 8.2 Hz, 1H, H3), 8.84 (s, 1H, H10), 8.59 (d, *J* = 6.4 Hz, 1H, H7), 8.48 (dd, *J* = 1.2 Hz, 8.2 Hz, 1H, H5), 8.03–7.95 (m, 2H, H2,6), 7.09 (s, 2H, H13,17), 3.80 (s, 6H, H18,20), 3.63 (s, 3H, H19). ^{13}C NMR (100 MHz, DMSO-*d*$_6$, δ, ppm): 170.4 (C11), 155.3 (C1), 152.6 (C14,16), 149.8 (C10), 142.4 (C3), 141.4 (C7), 136.9 (C15), 135.5 (C9), 135.2

(C5), 132.8 (C12), 129.6 (C8), 127.7 (C6), 127.4 (C4), 123.1 (C2), 97.4 (C13,17), 60.1 (C18,20), 55.8 (C19).

$[Fe(L)_2]Cl\cdot3H_2O\cdot0.25DMF$ ($4\cdot3H_2O\cdot0.25DMF$). To a solution of **HL** (0.40 g, 1.0 mmol) in DMF (15 mL) was added dropwise a solution of $FeCl_3$ (0.14 g, 0.5 mmol) in ethanol (10 mL). The reaction mixture was stirred at room temperature for 1 h. The black-brown crystals formed after 24 h were filtered, washed with cold ethanol (2 × 5 mL) and diethyl ether (3 × 15 mL). Yield: 0.33 g (65%). Calcd for $C_{40.75}H_{45.75}ClFeN_{8.25}O_{9.25}S_2$ (M_r 954.53), %: C, 51.28; H, 4.83; N, 12.11; S, 6.72. Found, %: C, 51.51; H, 4.68; N, 11.82; S, 6.41. Positive ion ESI MS: *m*/*z* 846.19 attributed to $[Fe^{III}L_2]^+$; calcd *m/z* 846.17. Negative ion ESI MS: *m/z* 844.17 attributed to $[Fe^{III}(L-H)_2]^-$; calcd *m/z* 844.16.

Methods

NMR spectroscopy. The ^1H and ^{13}C NMR spectra were recorded on a Bruker Avance Neo 400-MHz spectrometer using the standard parameter sets as provided by Bruker. Deuterated dimethyl sulfoxide, DMSO-d_6, was used as solvent. The chemical shifts are given relative to the solvent residual peaks: 2.50 ppm for ^1H and 39.5 ppm for ^{13}C. The assignment of the signals in the NMR spectra was based on additional 2D homo- and hetero-nuclear correlation experiments ($^1H,^1H$ COSY, $^1H,^{13}C$ HSQC and $^1H,^{13}C$ HMBC).

UV−vis spectrophotometry. An Agilent Cary 8454 and an Agilent Cary 3500 diode array spectrophotometer were used to measure the UV−vis spectra. Distribution coefficient (*D*) values for the compounds were determined by the traditional shake flask method in *n*-octanol/buffered aqueous solution at pH = 7.4 (20 mM HEPES). **HL** and complex **3** were dissolved in *n*-octanol (presaturated with the aqueous buffer), and an aqueous-to-organic phase ratio of 20:1 was used for these samples. In contrast, the Cu(II) complexes **1** and **2** were dissolved in the aqueous buffer (presaturated with *n*-octanol), and for these samples, the phase ratio was 1:1. The procedure was the same as that described previously.[15] The stability of the compounds was monitored by UV−vis spectrophotometry at pH 7.4 (20 mM HEPES buffer) at 25.0 °C for 24 h. **HL** was tested in a 50% (*v*/*v*) DMSO/H_2O mixture, while complexes **1–4** were investigated in 2% (*v*/*v*) DMSO/H_2O. The concentration of the compounds was 20 μM.

The UV−vis spectrophotometric titrations were performed following the procedures and experimental setups described elsewhere.[16,17] During the titrations, the **HL** concentration was 30 μM. In the Fe(III) complex formation studies, the metal-to-ligand ratios were 1:1, 1:2, and 1:3. The measurements were carried out in the pH range of 2.0–12.0 using an optical path length of 1 cm. The conditional stability of complexes **1** and **2** was investigated using EDTA displacement. This was performed in 30% (*v*/*v*) DMSO/H_2O solvent mixture, at an ionic strength of 0.1 M and a constant pH (5.9), which was adjusted using MES buffer. In the samples, the complexes concentration was ~20 μM, and the EDTA concentration varied between 5 μM and 4 mM. The spectra were recorded after a 6 h equilibration period (following an investigation of the reaction's time dependence) at a path length of 1 cm.

X-ray crystallography. SC-XRD data were collected on XtaLAB Synergy, Dualflex, HyPix diffractometer using Cu Kα radiation. The unit cell determination and data integration were carried out using the CrysAlisPro package.[18] Multi-scan correction for absorption was applied. The structures were solved by the intrinsic phasing method with the SHELXT[19] program and refined by the full-matrix least-squares method on F^2 with SHELXL.[20] Olex2 was used as an interface to the SHELX programs.[21] Non-hydrogen atoms were refined anisotropically. Hydrogen atoms attached to carbon were inserted in idealized positions and refined using a riding model. The H atoms of NH and OH groups were localized from Fourier maps and their positions were verified in accordance with hydrogen bonding parameters. Selected crystallographic data and structure refinement details are provided in Table S3, which also quots CCDC nos; these contain the supplementary crystallographic data for this paper.

Cell-free ROS generation and redox activity. EPR measurements were performed using an X-band Bruker EMX spectrometer (Germany). The spin trapping agent 5,5-dimethyl-1-pyrroline-*N*-oxide (DMPO, Sigma-Aldrich) was distilled before use (70 Pa; 80−90 °C). Standard settings during EPR spin trapping experiments were microwave frequency ~9.448 GHz; power of the microwave radiation 2 mW; sweep width 200 G; centre field 3350 G; modulation amplitude 2 G; time constant 20.5 ms; and sweep time 42 s, series of 10 spectra with 3 scans for each measurement. Optical spectra in dimethyl sulfoxide (DMSO, SeccoSolv max. 0.025% H_2O, Merck) were measured on a Shimadzu 3600 UV−vis−NIR spectrometer (Japan). Experiments with sodium ascorbate and spectroelectrochemical studies were performed on a UV−vis−NIR Avantes spectrometer (Model AvaSpec-2048_14-USB2). The cyclic voltammograms (CV) of investigated samples in 0.1 M tetrabutylammonium

hexafluorophosphate (n-Bu_4NPF_6, puriss quality from Fluka) as a supporting electrolyte were recorded using a single compartment electrochemical cell with a working Pt-disk electrode (from Ionode, Australia), a Pt wire as counter electrode and an Ag wire as a pseudoreference electrode at scan rate of 100 mV s^{-1}. All potentials in voltammetric studies were quoted vs ferricenium/ferrocene (Fc^+/Fc) redox couple (purchased from Sigma Aldrich). A Heka PG310USB (Lambrecht, Germany) potentiostat with a PotMaster 2.73 software package served for the potential control in voltammetric studies.

Molecular modeling calculations. The two Cu(II) complexes **1** and **2** (see Chart 1) were docked into the binding site of tubulin-colchicine (PDB ID: 4O2B, resolution 2.30 Å),[4] which was obtained from the Protein Data Bank (PDB).[6,7] The GOLD (v2025.3) software suite was used to prepare the crystal structures for docking, i.e., the hydrogen atoms were added, water molecules and expedients deleted and the co-crystallized ligand identified: tubulin-colchicine – LOC (*N*-[(7*S*)-1,2,3,10-tetramethoxy-9-oxo-6,7-dihydro-5*H*-benzo[d]heptalen-7-yl]ethanamide). The docking centre for the binding pocket was defined as the position of the co-crystallized LOC ligand with 10 Å radius. The scoring functions GoldScore (GS),[10] ChemScore(CS),[11,12] Piecewise Linear Potential (ChemPLP)[13] and Astex Statistical Potential (ASP)[14] was used in the GOLD (v2025.3) docking algorithm. The crystal structures of the complexes **1** and **2** were used for the docking.

mR2 RNR inhibition. The kinetics of $Y^{\bullet}$ reduction in mR2 RNR protein by **HL** and **1–4** were monitored by X-band (9.4 GHz) EPR spectroscopy at 30 K on a Bruker ELEXSYS II E540 EPR spectrometer with an Oxford Instruments ER 4112HV helium cryostat, essentially as described previously.[22,23] Mouse R2 was cloned, expressed, and purified as described previously,[24] with some modifications. In brief, the mouse R2 gene was cloned into a pET-3a plasmid (GenScript) and transformed into competent *Escherichia coli* BL21 (DE3)-T1^R cells (Sigma-Aldrich). Cells were grown in Terrific Broth medium containing 100 μg/mL ampicillin, protein expression was induced by adding isopropyl *β*-D-thiogalactoside (IPTG) to a final concentration of 0.5 mM at $OD_{600\ nm}$ = 0.7–0.8, and the cultures were incubated at 20 °C with vigorous shaking for 16 h before cells were harvested and frozen at −20 °C. Cells were thawed and dissolved in 50 mM Tris-HCl pH 7.5, 2 mM DTT, 10 mM EDTA, 5 μg/mL DNase, cOmplete Protease Inhibitor Cocktail tablet (Roche) in a 1:4 cell wet weight to buffer ratio and lysed by sonication. R2 protein was precipitated with 0.29 g/mL $(NH_4)_2SO_4$, dissolved in 50 mM Tris-HCl pH 7.5, 2 mM DTT, 2 mM EDTA and desalted through dialysis (SnakeSkin™

dialysis tubing, 10kDa MWCO, ThermoFisher Scientific). Desalted protein was filtered through a 0.45 µm filter (Sarstedt), applied on a XK 26/20 chromatography column packed with Q Sepharose (Cytiva) and eluted with a linear 0–0.5 M KCl gradient using 50 mM Tris-HCl pH 7.5, 2 mM DTT, 2 mM EDTA, 1 M KCl. Protein was further purified on a Superdex 75 or 200 Increase 10/300 GL column (Cytiva) in 50 mM HEPES pH 7.5, 100 mM NaCl. Protein fractions were pooled, concentrated in Amicon Ultra-15 filter units (30 kDA MWCO, Merck-Millipore), flash-frozen in lN_2, and stored at −20 °C. All chromatographic steps were performed on an Äkta purifier FPLC system (GE Healthcare) and all expression and purification steps were monitored by sodium dodecyl sulfate-polyacrylamide gel electrophoresis (SDS-PAGE). Protein concentration was determined using an Agilent Cary 60 spectrophotometer and the extinction coefficient $\varepsilon_{280\,nm} = 62000\ M^{-1}cm^{-1}$.[25] R2 was reconstituted with Fe as previously described,[16,17] resulting in the formation of diferric tyrosyl radical cofactor with 0.38 tyrosyl radical/Fe per monomer, which is in agreement with literature.[22,23] The samples containing 20 µM mR2 protein, 20 µM compound, and 2 mM dithiothreitol where indicated, were incubated for 0.5, 3 and 10 min at 298 K and quickly frozen in cold isopentane. The control samples contained 20 µM mR2 protein alone, and 2 mM DTT where indicated. The same sample was used for repeated incubations and was refrozen before each EPR measurement. All measurements were performed in duplicates.

The natural $Y^{\bullet}$ decay in control samples in absence and presence of DTT is shown in Figure S37. Note that upon incubation of the mR2 protein with DTT at 298 K, an initial decrease of $Y^{\bullet}$ is followed by its full recovery after 10 min. This is the result of the differic center reduction by DTT, which firstly leads to $Y^{\bullet}$ loss, followed by the radical reconstitution process, in which the di-iron(III)-μ-oxido cluster is reassembled, generating the stable $Y^{\bullet}$.[26]

Tubulin assays. The methods used for determination of the IC_{50} values for tubulin assembly and inhibition of [^{3}H]colchicine binding were as described previously.[27]

Cell culture. MCF-7 human breast cancer cells were expanded in αMEM supplemented with 10% fetal bovine serum (FBS) and 1% antibiotic-antimycotic at 37°C in a 5% humidified atmosphere until sufficient confluence was reached for experiments. For passaging, cells were detached using Accutase enzyme solution. Briefly, the culture medium was aspirated and the cell monolayer was rinsed with phosphate-buffered saline (PBS). TrypLE (1 mL, applied in two steps) was added to the flask and incubated at 37 °C in a 5% CO_2 atmosphere for up to 10 min,

with periodic microscopic monitoring to assess detachment. The flask was gently tapped as needed to facilitate complete cell release. Enzymatic activity was quenched by addition of an equal volume of complete growth medium. Cells were then pelleted by centrifugation at 300 × g for 2–5 min, the supernatant was discarded, and the pellet was resuspended in fresh culture medium.

Cell Viability Assessment – CellTiter-Glo® Luminescent Cell Viability Assay. The metabolic activity of MCF-7 cells following drug treatment was evaluated using the CellTiter-Glo® Luminescent Cell Viability Assay (Promega, Madison, WI, USA), according to the manufacturer's instructions. Briefly, cells were seeded at a density of 5,000 cells per well in opaque-walled, white 96-well plates and allowed to adhere at 37 °C overnight in a humidified atmosphere containing 5% CO_2. The following day, cells were exposed to 8 serial dilutions (1/3.16 step dilution factor) of the test compounds – HL, 1–4 – spanning a final concentration range of 3.18 nM to 10 μM, prepared from 10 mM stock solutions in dimethyl sulfoxide (DMSO). Nocodazole was included as a reference microtubule-destabilizing agent. After 48 h of treatment, the plates were equilibrated to room temperature for 30 min, and an equal volume of CellTiter-Glo® reagent was added to each well. The contents were mixed vigorously for 2 min to induce cell lysis, followed by a 10-min stabilization period at room temperature to allow the luminescent signal to equilibrate. Luminescence was recorded using a FLUOstar® Omega microplate reader (BMG Labtech, Germany), with the resulting signal being directly proportional to the amount of ATP present and, by extension, to the number of metabolically active cells. Dose–response curves and IC_{50} values were calculated using GraphPad Prism (nonlinear regression, four-parameter logistic model).

Fluorescence-Based Live/Dead Cell Discrimination. To independently assess cytotoxic effects at a single-cell resolution, a multiparametric fluorescence live/dead staining protocol was performed in parallel. MCF-7 cells were seeded at 5,000 cells per well in optically clear-bottomed, black-walled 96-well plates (Cellvis, Mountain View, CA, USA) suitable for high-content fluorescence microscopy and treated under identical conditions as described above.

Following 48 h exposure to **HL**, **1–4** and nocodazole, the culture medium was replaced with a staining solution containing Hoechst 33342 (1.25 μg/mL; nuclear counterstain for all cells, blue, ex/em 350/461 nm), Calcein-AM (0.5 μM; cytoplasmic marker of esterase activity in viable

cells, green, ex/em 490/515 nm), and propidium iodide (PI; 1 μM; membrane-impermeant dye selective for cells with compromised plasma membrane integrity, red, ex/em 535/617 nm).

Cells were incubated with the staining mixture at 37 °C for 60 min, after which fluorescence images were acquired using ImageXpress® Confocal HT.ai high-content screening system (Molecular Devices, San Jose, CA, USA). Image analysis was performed with MetaXpress software (Molecular Devices) using the Multi-Wavelength Cell Scoring module. Viable cells were defined as those exhibiting dual positivity for Hoechst and Calcein-AM fluorescence while remaining negative for PI signal, whereas dead cells were identified as PI-positive events. Untreated cells served as the negative control, and cells exposed to Triton X-100 for 30 min prior to staining were used as a positive control for cell death. Dose–response relationships and IC_{50} values were derived from the quantified live-cell counts using GraphPad Prism version 10.6.1.

Cell Cycle Analysis by Immunofluorescence Microscopy. To investigate the effects of compounds **1** and **2** on cell cycle progression, MCF-7 cells were processed in parallel with those used for the live/dead viability assay, with the modification that all culture wells were pre-coated with fibronectin (33 μg/mL) for 1 h prior to cell seeding, to promote uniform cell adhesion and spreading on the imaging substrate.

Cells were seeded and allowed to adhere under standard culture conditions (37 °C, 5% CO_2) overnight. The following day, compounds were applied at a single fixed concentration of 3.16 μM and incubated for 21 h.

Immunofluorescence Staining. Following treatment, cells were processed for immunofluorescence according to a sequential fixation, permeabilization, and blocking protocol. Briefly, cells were fixed with 4% paraformaldehyde (PFA) at room temperature for 15 min, followed by permeabilization with 0.5% Triton X-100 for 15 min. Non-specific binding was blocked by incubation with 3% bovine serum albumin (BSA) for 60 min. Cells were then incubated at 4 °C overnight with the following primary antibodies: a mouse monoclonal anti-α-tubulin antibody (clone 236-10501, ThermoFisher Scientific, cat. no. A11126), used to visualize the microtubule network and mitotic spindle architecture, and a recombinant rabbit monoclonal antibody targeting phospho-Histone H3 (Ser10) (clone 9H12L10, ThermoFisher Scientific), a well-established marker of mitotic entry and chromosome condensation.

The following day, cells were incubated at room temperature for 1 h with species-appropriate secondary antibodies: goat anti-mouse IgG (H+L) conjugated to DyLight™ 488 (ThermoFisher Scientific, cat. no. 35502; 1:250 dilution), and donkey anti-rabbit IgG (H+L), cross-adsorbed, conjugated to DyLight™ 755 (ThermoFisher Scientific, cat. no. SA5-10043; 1:250 dilution). Nuclear counterstaining was performed with Hoechst 33342, as described above.

Image Acquisition and Quantitative Analysis. Stained cultures were examined using two complementary imaging platforms. Qualitative morphological assessment of mitotic figures, spindle organization, and nuclear morphology was conducted on a Zeiss Axio Observer 7 widefield fluorescence microscope. Quantitative high-content analysis was performed on the ImageXpress® Confocal HT.ai high-content screening system (Molecular Devices, San Jose, CA, USA).

For quantitative cell cycle profiling, image analysis was carried out in MetaXpress software v. 6.0 using the Cell Cycle application module. DNA content was assessed from the Hoechst 33342 fluorescence signal to discriminate G1, S, and G2/M cell populations, while phospho-Histone H3 (Ser10) immunoreactivity was used to specifically identify cells in mitosis. A minimum of 20 microscopic fields per condition were acquired and included in the quantitative analysis to ensure statistical robustness.

Statistical analysis. All statistical analyses were performed using **GraphPad Prism version 10.6.1** (GraphPad Software). Data are presented as **mean ± SEM**. For dose–response experiments assessing cell viability, statistical significance was evaluated by **one-way ANOVA followed by Dunnett's multiple-comparisons test**, with each treatment concentration compared with the untreated control (0 μM). For cell-cycle analysis, data were analyzed using **two-way ANOVA followed by Dunnett's multiple-comparisons test**, with each cell-cycle phase for each treatment compared with the corresponding phase in the untreated control. Three independent experiments (N = 3) were performed for both types of analysis. For cell-viability assays, three replicate measurements were obtained per experiment for CellTiter-Glo and four replicate measurements per experiment for image-based cell counting. For cell-cycle analysis, three independent experiments (N = 3) were performed, with eight replicate fields analyzed per condition in each experiment (24 fields per condition in total). Significance is indicated as *ns* (not significant), $p < 0.05$ (*), $p < 0.01$ (**), $p < 0.001$ (***), and $p < 0.0001$ (****).

References

1 C. A. Lipinski, Lead and drug-like compounds: the rule-of-five revolution. *Drug Disc. Today: Technol.,* 2004, **1**, 337–341.

2 C. A. Lipinski, F. Lombardo, B. W. Dominy, P. J. Feeney, Experimental and computational approaches to estimate solubility and permeability in drug discovery and development settings, *Adv. Drug Del. Rev.*, 2012, **64**, 4–17.

3 D. F. Veber, S. R. Johnson, H.-Y. Cheng, B. R. Smith, K. W. Ward, K. D. Kopple, Molecular properties that influence the oral bioavailability of drug candidates. *J. Med. Chem.*, 2002, **45**, 2615–2623.

4 A. E. Prota, F. Danel, F. Bachmann, K. Bargsten, R. M. Buey, J. Pohlmann, S. Reinelt, H. Lane, M. O. Steinmetz, The Novel Microtubule-Destabilizing Drug BAL27862 Binds to the Colchicine Site of Tubulin with Distinct Effects on Microtubule Organization. *J. Mol. Biol.*, 2014, **426**, 1848–1860.

5 R. B. G. Ravelli, B. Gigant, P. A. Curmi, I. Jourdain, S. Lachkar, A. Sobel, M. Knossow, Insight into tubulin regulation from a complex with colchicine and a stathmin-like domain *Nature*, 2004, **428**, 198–202.

6 H. M. Berman, The Protein Data Bank. *Nucleic Acids Res.*, 2000, **28**, 235–242.

7 H. Berman, K. Henrick, H. Nakamura, Announcing the worldwide Protein Data Bank. *Nat. Struct. Mol. Biol.*, 2003, **10**, 980.

8 Hocquet, M. Langård, An Evaluation of the MM+ Force Field. *J. Mol. Model.*, 1998, **4**, 94–112.

9 M. N. M. Milunovic, K. Ohui, I. Besleaga, T. V. Petrasheuskaya, O. Dömötör, É. A. Enyedy, D. Darvasiova, P. Rapta, Z. Barbieriková, D. Vegh, S. Tóth, J. Tóth, N. Kucsma, G. Szakács, A. Popović-Bijelić, A. Zafar, J. Reynisson, A. D. Shutalev, R. Bai, E. Hamel, V. B. Arion, Copper(II) Complexes with Isomeric Morpholine-Substituted 2-Formylpyridine Thiosemicarbazone Hybrids as Potential Anticancer Drugs Inhibiting Both Ribonucleotide Reductase and Tubulin Polymerization: The Morpholine Position Matters. *J. Med. Chem.*, 2024, **67**, 9069–9090.

10 G. Jones, P. Willett, R. C. Glen, A. R. Leach, R.; Taylor, Development and validation of a genetic algorithm for flexible docking. *J. Mol. Biol.*, 1997, **267**, 727–748.

11 M. D. Eldridge, C. W. Murray, T. R. Auton, G. V. Paolini, R. P. Mee, Empirical scoring functions: I. The development of a fast empirical scoring function to estimate the binding affinity of ligands in receptor complexes. *J. Comp.-Aid. Mol. Des.*, 1997, **11**, 425–445.

12 M. L. Verdonk, J. C. Cole, M. J. Hartshorn, C. W. Murray, R. D. Taylor, Improved protein-ligand docking using GOLD.*Proteins: Struct., Funct. Bioinform.*, 2003, **52**, 609–623.

13 O. Korb, T. Stützle, T. E. Exner, Empirical scoring functions for advanced protein-ligand docking with PLANTS. *J. Chem. Inf. Model.*, 2009, **49**, 84–96.

14 W. T. M. Mooij, M. L. Verdonk, General and targeted statistical potentials for protein-ligand Interactions. *Proteins: Struct., Funct. Bioinform.*, 2005, **61**, 272–287.

15 É. A. Enyedy, É. Zsigó, N. V. Nagy, C. R. Kowol, A. Roller, B. K. Keppler and T. Kiss, Complex-Formation Ability of Salicylaldehyde Thiosemicarbazone towards Zn^{II}, Cu^{II}, Fe^{II}, Fe^{III} and Ga^{III} Ions. *Eur. J. Inorg. Chem.*, 2012, 4036–4047.

16 V. Pósa, B. Hajdu, G. Tóth, O. Dömötör, C. R. Kowol, B. K. Keppler, G. Spengler, B. Gyurcsik and É. A. Enyedy, The coordination modes of (thio)semicarbazone copper(II) complexes strongly modulate the solution chemical properties and mechanism of anticancer activity. *J. Inorg. Biochem.*, 2022, **231**, 111786.

17 S. Hager, V. F. S. Pape, V. Pósa, B. Montsch, L. Uhlik, G. Szakács, S. Tóth, N. Jabronka, B. K. Keppler, C. R. Kowol, É. A. Enyedy and P. Heffeter, High Copper Complex Stability and Slow Reduction Kinetics as Key Parameters for Improved

Activity, Paraptosis Induction, and Impact on Drug-Resistant Cells of Anticancer Thiosemicarbazones. *Antioxid. Redox Signal.*, 2020, **33**, 395–414.
18 Rigaku Oxford Diffraction. CrysAllis Pro Software System. Rigaku Corporation, Oxford, UK 2015.
19 G. M. Sheldrick, SHELXT– Integrated space-group and crystal-structure determination. *Acta Crystallogr. Sect. Found. Adv.*, 2015, **71**, 3–8.
20 G. M. Sheldrick, Crystal structure refinement with SHELXL. *Acta Crystallogr. Sect. C Struct. Chem.*, 2015, **71**, 3–8.
21 O. V. Dolomanov, L. J. Bourhis, R. J. Gildea, J. A. K. Howard and H. Puschmann, *OLEX2*: a complete structure solution, refinement and analysis program. *J. Appl. Crystallogr.*, 2009, **42**, 339–341.
22 A. Popović-Bijelić, C. R. Kowol, M. E. S. Lind, J. Luo, F. Himo, É. A. Enyedy, V. B. Arion and A. Gräslund, Ribonucleotide reductase inhibition by metal complexes of Triapine (3-aminopyridine-2-carboxaldehyde thiosemicarbazone): A combined experimental and theoretical study. *J. Inorg. Biochem.*, 2011, **105**, 1422–1431.
23 K. Ohui, E. Afanasenko, F. Bacher, R. L. X. Ting, A. Zafar, N. Blanco-Cabra, E. Torrents, O. Dömötör, N. V. May, D. Darvasiova, É. A. Enyedy, A. Popović-Bijelić, J. Reynisson, P. Rapta, M. V. Babak, G. Pastorin and V. B. Arion, New Water-Soluble Copper(II) Complexes with Morpholine-Thiosemicarbazone Hybrids: Insights into the Anticancer and Antibacterial Mode of Action. *J. Med. Chem.*, 2019, **62**, 512–530.
24 M. F. Zaltariov, M. Hammerstad, H. J. Arabshahi, K. Jovanović, K. W. Richter, M. Cazacu, S. Shova, M. Balan, N. H. Andersen, S. Radulović, J. Reynisson, K. K. Andersson and V. B. Arion, New Iminodiacetate – Thiosemicarbazone Hybrids and Their Copper(II) Complexes Are Potential Ribonucleotide Reductase R2 Inhibitors with High Antiproliferative Activity. *Inorg. Chem.*, 2017, **56**, 3532–3549.
25 G. J. Mann, A. Gräslund, E. I. Ochiai, R. Ingemarson, L. Thelander, Purification and Characterization of Recombinant Mouse and Herpes Simplex Virus Ribonucleotide Reductase R2 Subunit. *Biochemistry*, 1991, **30**, 1939–1947.
26 A. Gräslund, A. Ehrenberg, L. Thelander, Characterization of the free radical of mammalian ribonucleotide reductase. *J. Biol. Chem.*, 1982, **257**, 5711−5715.
27 Y. Saito, Y. Taniguchi, S. Hirazawa, Y. Miura, H. Tsurimoto, T. Nakayoshi, A. Oda, E. Hamel, K. Yamashita, M. Goto and K. Nakagawa-Goto, Effects of substituent pattern on the intracellular target of antiproliferative benzo[b]thiophenyl chromone derivatives. *Eur. J. Med. Chem.*, 2021, **222**, 113578.
