# Supplementary material for: Metal thiosemicarbazonates as dual mR2 RNR and colchicine-site tubulin inhibitors: from biochemical inhibition to mitotic arrest in MCF-7 breast cancer cells
Source: Inorg Chem Front. 2026 Sep 3;13(22):9371–91. doi: 10.1039/d6qi01591c (PMC13592152; doi:10.1039/d6qi01591c)
Supplement: Supplementary file 4 [file QI-013-D6QI01591C-s002.pdf]

Supplementary Information (SI) for Inorganic Chemistry Frontiers.
This journal is © the Partner Organisations 2026

-----Original Message-----
From: Mikrolabor Phys-Chemie <mikrolp6@login.univie.ac.at> On Behalf Of Mikroanalytisches Laboratorium
Sent: Thursday, September 21, 2023 7:52 PM
To: Vladimir Arion <vladimir.arion@univie.ac.at>
Cc: Oleg Palamarciuc <Oóleg.palamarciuc@univie.ac.at>
Subject: Analysenergebnisse

Universitaet Wien
Institut fuer Anorganische Chemie
Univ-Prof.Dr.Vladimir Arion
Waehringer Str.42

A-1090 WIEN

Analysenschein 0923/1238 Am 21.09.2023 erreichte uns Ihr Auftrag vom 20.09.2023. Die gewuenschten Unter- suchungen wurden durchgefuehrt und wir koennen Ihnen die folgenden Ergebnisse uebermitteln.

Probenbez.	Anal-Nr	w-% C	w-% H	w-% N	w-% S	
OLPA 75	230921/332	52,21	6,53	10,33	13,02	0,865 mg
	230921/333	52,40	6,63	10,29	13,16	1,350 mg
OLPA 75.1	230921/328	51,12	4,83	6,41	14,07	0,941 mg
	230921/329	51,23	4,80	6,35	14,35	0,986 mg
OLPA 75.2	230921/330	46,14	5,75	16,02	12,35	0,854 mg
	230921/331	46,41	5,89	16,06	12,41	1,424 mg

Mit freundlichen Gruessen

Wien, 21. September 2023

Mag.Johannes THEINER
